# Taxonomy, phylogeny, and evolutionary diversification of spider-pathogenic fungi from China (*Hypocreales*, *Ascomycota*)

**DOI:** 10.3897/imafungus.17.171548

**Published:** 2026-04-06

**Authors:** Xiao-Yun Chang, Yan-Jia Qi, Ting Wang, Zeng-Zhi Li, Guang-Shuo Li, Bo Huang, Ming-Jun Chen

**Affiliations:** 1 Anhui Province Key Laboratory of Green Control for Major Forestry Pests, Anhui Agricultural University, Hefei 230036, China Anhui Agricultural University Hefei China; 2 College of Forestry and Grassland, Nanjing Forestry University, Nanjing 210037, China Key Laboratory of Biology and Sustainable Management of Plant Diseases and Pests of Anhui Higher Education Institutes, Anhui Agricultural University Hefei China; 3 Zhejiang BioAsia Pharmaceutical Institute, Pinghu, Zhejiang, China Nanjing Forestry University Nanjing China; 4 Key Laboratory of Biology and Sustainable Management of Plant Diseases and Pests of Anhui Higher Education Institutes, Anhui Agricultural University, Hefei 230036, China Zhejiang BioAsia Pharmaceutical Institute Pinghu China

**Keywords:** Evolution, molecular clock, morphology, new taxa, phylogeny

## Abstract

Spider-pathogenic fungi serve as critical regulators of spider populations in natural systems, playing irreplaceable roles in maintaining ecological balance and also serving as reservoirs of bioactive compounds. Despite recent taxonomic refinements at the generic level, their broader phylogenetic diversity remains significantly underrepresented compared to entomopathogenic fungi. In this study, we collected several novel spider-pathogenic fungi from various provinces in China and conducted comprehensive taxonomic and phylogenetic analyses. Based on integrated morphological characterization and multigene phylogenetic analyses of five loci (ITS, nrLSU, *TEF1*, *RPB1*, and *RPB2*), eight novel species are described and illustrated: *Arachnidicola* (1), *Gamszarella* (1), *Gibellula* (2), *Hevansia* (1), and *Liangia* (1) within *Cordycipitaceae*; *Husseyia* (1) within *Clavicipitaceae*; and *Purpureocillium* (1) within *Ophiocordycipitaceae*. Additionally, six new combinations are proposed, one Chinese new record is reported, the type specimen of one known species is revised, and five potential cryptic species are identified. Our phylogenetic analyses provide robust evidence for the taxonomic placement of *Chlorocillium* and *Husseyia* within *Clavicipitaceae*. Molecular clock analysis, utilizing a dataset of five loci from 648 fungal strains, estimated the stem and crown ages of *Hypocreales* and indicated that spider-pathogenic fungi likely emerged during the mid-Cretaceous and subsequently diversified. Multiple lineages displayed marked trends toward host specialization, suggesting that these fungi developed highly efficient parasitic strategies to exploit constrained ecological niches. This research substantially expands the documented diversity of araneopathogenic fungi, providing a robust phylogenetic framework for elucidating their evolutionary origins and diversification patterns, while offering valuable biological resources for future biotechnological and ecological applications.

## Introduction

Spiders (*Arachnida*, *Araneae*) represent one of the most diverse groups of terrestrial arthropods, with over 53,000 described species (World Spider Catalog 2026) occupying a vast array of ecosystems globally (Dimitrov and Hormiga 2021; Lüddecke et al. 2022). Entomopathogenic fungi serve as critical natural mortality factors for arthropods. While more than 700 species of entomopathogenic fungi have been documented from insect hosts and studied extensively (Consolo et al. 2003), fungi that specifically target spiders have historically received significantly less attention in general arachnological literature, where they are frequently conflated with insect-pathogenic fungi (Bradley 2012; Nyffeler and Hywel-Jones 2024). Recognizing their ecological uniqueness, Evans and Samson (1987) formally designated these fungi as a distinct ecological guild, introducing the terms “araneogenous fungi” or “araneopathogenic fungi” to describe species that specifically infect and kill spiders.

The investigation of spider-pathogenic fungi traces its origins to the first documented report from Germany in 1817, which described a fungal infection of the spider *Phoneutria nigriventer*, This fungus was initially named *Isaria arachnophila* (Gray 1858), but was subsequently identified as *Akanthomyces aranearum* (the anamorph of *Cordyceps thaxteri*) (Evans and Samson 1987). Additionally, the first sexual morph documented from a spider was *C. caloceroides*, described in Cuba by Berkeley and Curtis (1869). As research progressed, the recognized diversity of spider-pathogenic fungi expanded substantially, with species concentrated primarily within four families of the order *Hypocreales*: *Cordycipitaceae*, *Ophiocordycipitaceae*, *Clavicipitaceae* and *Nectriaceae*.

*Cordycipitaceae* currently harbors the majority of described spider-pathogenic fungi, representing approximately three-quarters of all known taxa. Obligate spider pathogens in genera such as *Gibellula* (including its sexual form, *Torrubiella*) and *Hevansia* demonstrate particular abundant and widespread distribution, predominantly parasitizing spiders inhabiting leaf undersurfaces (Kepler et al. 2017; Kuephadungphan et al. 2022; Mongkolsamrit et al. 2022). Recent taxonomic advances have led to the establishment of novel genera including *Jenniferia*, *Polystromomyces*, *Bhushaniella*, and *Arachnidicola* to accommodate newly discovered species, thereby substantially enriching the phylogenetic diversity of spider-pathogenic fungi (Mongkolsamrit et al. 2022; Mongkolsamrit et al. 2023; Khonsanit et al. 2024). While certain generalist entomopathogens within *Cordyceps* and *Beauveria* have demonstrated infectivity toward spider hosts (Shrestha et al. 2014; Chen et al. 2017), additional genera including *Engyodontium*, *Gamszarea*, *Gamszarella*, *Lecanicillium*, *Simplicillium*, *Leptobacillium*, *Parengyodontium*, and *Pseudogibellula* encompass species with confirmed or suspected spider pathogenicity (Sukarno et al. 2009; Leplat et al. 2020; Chen et al. 2025b; Lu et al. 2025).

Within *Ophiocordycipitaceae*, *Purpureocillium atypicola* (sexual morph: *Cordyceps cylindrica*) emerges as a particularly widespread spider-pathogenic fungus, documented from at least 17 spider species across 10 families (Greenstone et al. 1987; Spatafora et al. 2015). In contrast, other genera within this family, including *Ophiocordyceps*, *Hirsutella*, and *Tolypocladium*, are primarily associated with a diverse range of arthropod hosts, with only a limited number of species documented from spiders (Evans and Samson 1982; Montalva et al. 2019; Shrestha et al. 2019). The family *Clavicipitaceae* has historically been regarded as primarily consisting of plant-pathogenic fungi, with some lineages having evolved insect pathogenicity. However, the first confirmed spider-associated genera, *Neoaraneomyces* and *Pseudometarhizium*, were not reported until 2022; the latter was subsequently transferred to *Chlorocillium*, marking the initial definitive documentation of spider-pathogenic fungi within this family (Chen et al. 2022b). Notably, *Chlorocillium* was originally established by Zare and Gams (2016) to accommodate *C. griseum*, a confirmed spider-pathogenic species, although its familial placement remained uncertain initially. Subsequent molecular investigations confirmed the genus within *Clavicipitaceae* and facilitated the discovery of several additional spider-associated species (Chen et al. 2024a; Ding et al. 2024). Currently, ten species have been described within *Chlorocillium*, six of which demonstrate confirmed spider parasitism, establishing it as a representative spider-pathogenic genus within the family (Chen et al. 2024a; Tan and Shivas 2024a, 2025b). Furthermore, Lin et al. (2025) reported *Conoideocrella gongyashanensis*, a new spider-pathogenic species within the *Clavicipitaceae* genus *Conoideocrella*, further expanding the family’s recognized diversity.

Additionally, two species of spider-pathogenic fungi have been identified in the nectriaceous genus *Clonostachys* (Chen et al. 2016; Wang et al. 2023). Genera of uncertain taxonomic placement, including *Husseyia* and *Clathroconium*, have also been reported to contain spider-pathogenic representatives (Mercado Sierra et al. 1988; Tan and Shivas 2023c). Furthermore, certain fungi outside *Hypocreales*, including *Aspergillus*, *Mucor* and *Penicillium*, have been isolated from spider cadavers. However, their pathogenicity remains questionable, as they likely represent opportunistic colonizers or saprophytic organisms rather than true pathogens (Rong and Grobbelaar 1998; Evans 2013; Nyffeler and Hywel-Jones 2024).

To date, more than 110 spider-pathogenic fungal species have been described from over 20 genera within *Hypocreales* (Chen et al. 2025b). These fungi are widely distributed, occurring in tropical regions such as Brazil, China, Ghana, Indonesia, and Thailand, as well as in temperate forests of North America and even Arctic environments (Bristowe 1948; Kuephadungphan et al. 2019; Mongkolsamrit et al. 2022). In addition to regulating spider populations and indirectly shaping arthropod community dynamics (Haupt 2002; Yang et al. 2024), spider-pathogenic fungi represent an important reservoir of bioactive secondary metabolites with antimicrobial or pharmaceutical potential (Isaka et al. 2013; Isaka et al. 2014; Kuephadungphan et al. 2014; Kuephadungphan et al. 2019). Despite growing interest in this broadly distributed group, available evidence indicates that their documented diversity remains incomplete, particularly in underexplored regions (Nyffeler and Hywel-Jones 2024; Wang et al. 2024a). Moreover, phylogenetic relationships among several spider-pathogenic lineages remain unresolved, underscoring the need for further species discovery to clarify their hidden diversity, evolutionary relationships, and evolutionary history.

Molecular clock analyses, which estimate divergence times based on genetic distances calibrated with paleontological evidence, have significantly advanced our understanding of fungal evolution history (Kumar 2005; Hyde et al. 2021). Within *Hypocreales*, well-preserved fossils such as *Paleoophiocordyceps coccophagus* in amber have provided reliable temporal calibration points for inferring divergence events (Sung et al. 2008). Previous studies have estimated the crown age of *Hypocreales* between 106 and 200 Mya (Sung et al. 2008; Samarakoon et al. 2016; Wei et al. 2022), although considerable uncertainty remains due to differences in calibration strategies and taxon sampling. Moreover, reconstructions of ancestral host associations within *Hypocreales* remain incongruent among studies. For example, Sung et al. (2008) inferred a plant-associated ancestor for *Clavicipitaceae*, whereas subsequent analyses suggested animal- or arthropod-associated ancestral states for several entomopathogenic lineages (Wei et al. 2022). In contrast, Kobmoo et al. (2023) proposed that the ancestor of *Cordycipitaceae* was more likely an environmental or saprotrophic fungus rather than directly host-associated. Overall, these findings present partly conflicting interpretations, which may require further investigation to be clarified. Despite these advances, the divergence times of spider-pathogenic fungal lineages, including both stem- and crown-group node ages, remain largely unexplored. Targeted molecular clock analyses are therefore needed to reconstruct the evolutionary origins and diversification patterns of spider-pathogenic fungi and to better understand their host-association histories.

This study aims to integrate comprehensive morphological and multi-locus sequence data to elucidate the taxonomic relationships and evolutionary history of spider-pathogenic fungi within *Hypocreales*. Through molecular clock analyses, we estimate divergence times and reconstruct the evolutionary trajectories of major spider-pathogenic fungi lineages. Our findings will provide new insights into the evolutionary origin and diversification patterns of these ecologically and economically significant fungi, establishing a robust foundational framework for future research, conservation, and biotechnological exploitation.

## Materials and methods

### Sample collection, isolation and morphological observations

Between 2021 and 2025, 166 specimens were collected from various Chinese forest regions, primarily during field surveys conducted from June to September each year. This period is characterized by abundant rainfall and a warm, humid climate in mountainous areas, providing favorable conditions for the proliferation of spider-pathogenic fungi. Collections were made by inspecting typical spider microhabitats in forests, including tree bark, decaying wood, the upper and lower surfaces of leaves, moist rock faces, stones and riverside walls, to identify spiders infected by fungi. A map of the sampling sites and associated meteorological data are provided in Suppl. material 1: table SS1, fig. S1. Collected specimens were immediately labeled, placed individually in specimen boxes with appropriate moisture retention treatment, and then transported to the laboratory for further isolation and identification. Observations were conducted under a stereomicroscope (Olympus SZX16).

Fungal isolates were obtained by transferring fresh conidia or mycelium from the infected spider cadavers onto potato dextrose agar (PDA) supplemented with 0.1 g/L streptomycin and 0.1 g/L potassium penicillin (Wang et al. 2024b). After fungal colonies emerged from the plated samples, a piece of mycelium from the colony margin was subcultured onto fresh PDA plates and incubated at 25 °C for 14 days under a 12 h light/dark cycle. Holotype specimens and ex-type cultures were deposited at the Anhui Province Key Laboratory of Green Control for Major Forestry Pests, Anhui Agricultural University, Hefei, China. MycoBank numbers were obtained according to procedures outlined in MycoBank (http://www.MycoBank.org) (Robert et al. 2013).

Colony characteristics were recorded from PDA cultures incubated at 25 °C for 14 days, including colony diameter, presence of octahedral crystals, and colony pigmentation (front and reverse). Additional media types were used to provide complementary data on colony morphology. The Methuen Handbook of Colour (Kornerup and Wanscher 1978) was used as the color guide for the description of colony pigmentation. For examination of microstructures, sexual structures were taken from fresh specimens, or a small portion of mycelium was scraped from cultures. Samples were mounted in lactophenol cotton blue or 20% lactic acid solution and examined under a compound microscope (Carl Zeiss Axioscope 5) for measurements and observations of asexual morphological features, including perithecia, asci, ascospores, conidiophores, phialides or conidiogenous cells and conidia (Mongkolsamrit et al. 2018; Chen et al. 2022a).

### DNA extraction and sequencing

Fungal DNA was extracted from dried specimens or cultured mycelia using a modified CTAB method (Mongkolsamrit et al. 2009).

Polymerase chain reaction (PCR) amplification was applied to amplify five loci, including ITS, nrLSU, *TEF1*, *RPB1*, and *RPB2*, which are commonly used in entomogenous fungi. The following primer pairs were used: ITS1/ITS4 for ITS (White et al. 1990), LROR/LR5 for nrLSU (Vilgalys and Hester 1990), *EF1*-985F/*EF1*-2218R for *TEF1* (Rehner and Buckley 2005), C*RPB1*/*RPB1*Cr for *RPB1* (Castlebury et al. 2004) and f*RPB2*-5F/f*RPB2*-7cR for *RPB2* (Liu et al. 1999). The PCR amplification system consisted of 2 min at 94 °C, followed by 35 cycles of 30 s at 94 °C, 30 s at 48 °C (ITS, nrLSU and *RPB1*), and 45 s at 72 °C and a final step of 2 min at 72 °C. Different annealing temperatures were used according to the genomic region to be amplified: 52 °C for *RPB2* and 58 °C for *TEF1*. PCR products were visualized on agarose gel after electrophoresis and then sent for sequencing to Beijing Liuhe Huada Gene Technology Company. The resulting sequences were submitted to GenBank for sequencing.

### Phylogenetic analyses

The ITS, nrLSU, *TEF1*, *RPB1* and *RPB2* sequences used for phylogenetic analyses included sequences newly generated in this study as well as reference sequences from other *Hypocreales* taxa downloaded from GenBank (see Table 1). Sequences generated in this study were examined using BioEdit v7.7.1.0 to identify ambiguous bases, and base misalignments were corrected manually when necessary (Hall et al. 2011). These sequences were then combined with the reference sequences, and multiple sequence alignments were performed using MAFFT v7.313 (Katoh and Standley 2013). Aligned sequences were manually trimmed, removing bases present in less than 75% of the taxa. The alignment lengths before and after trimming for each locus are summarized in Suppl. material 1: table SS2. These sequences were then concatenated into a single dataset using PhyloSuite v1.2.3 (Xiang et al. 2023). The maximum likelihood (ML) analyses were conducted using IQ-TREE v. 2.1.3 under partitioned models (Minh et al. 2020), with the best-fit substitution model automatically selected based on the Bayesian Information Criterion (BIC). Bayesian inference (BI) was performed using MrBayes v3.2.6 (Ronquist et al. 2012), and the best-fit substitution model for each dataset was estimated under the Akaike Information Criterion (AIC) using MrModeltest v2.3 (Nylander 2004). Posterior probabilities (PP) were calculated using Markov Chain Monte Carlo (MCMC) sampling under the estimated evolutionary model. Four independent Markov chains were run for 20 million generations, with trees sampled every 1000 generations. The run was automatically terminated when the average standard deviation of split frequencies dropped below 0.01. The first 25% of trees were discarded as burn-in, and the remaining trees were used to calculate posterior probabilities in a majority-rule consensus tree. Phylogenetic trees were visualized and edited using the Interactive Tree of Life (iTOL) online platform (Letunic and Bork 2019).

**Table 1.** GenBank accession numbers of strains used for five-locus phylogenetic analyses in this study. Sequences obtained in this study are shown in bold. “–” represents missing data. ^T^ex-type species.

Strain code	Species	GenBank accession number					References
		ITS	nrLSU	*TEF1*	*RPB1*	*RPB2*	
NTUPPMCC 20-060^T^	*Akanthomyces taiwanicus*	MT974202	MT974356	MW200213	MW200221	MW200230	Chuang et al. (2024)
BCC 72638^T^	*Akanthomyces tortricidarum*	MT356076	MT356088	MT478004	MT477997	MT477992	Kubátová et al. (2024)
BCC 41868	*Akanthomyces tortricidarum*	MT356077	MT356089	MT477985	MT477998	MT478008	Khonsanit et al. (2024)
XX21081764	*Akanthomyces xixiuensis*	OP693460	OP693480	OP838887	OP838889	OP838891	Liu et al. (2024)
HKAS125851^T^	*Akanthomyces xixiuensis*	NR_191207	NG_243156	OP838888	OP838890	OP838892	Liu et al. (2024)
GY29011^T^	*Arachnidicola araneicola*	MK942431	-	MK955950	MK955944	MK955947	Pu et al. (2025)
GZUIF DX2^T^	*Arachnidicola araneogena*	MH978179	-	MH978187	MH978182	MH978185	Chen et al. (2018)
YFCC 1811934	*Arachnidicola araneogena*	OQ509518	OQ509505	OQ506281	OQ511530	OQ511544	Wang et al. (2024d)
YFCC 2206935	*Arachnidicola araneogena*	OQ509519	OQ509506	OQ506282	OQ511531	OQ511545	Wang et al. (2024d)
CQ05621^T^	*Arachnidicola bashanensis*	OQ300412	OQ300420	OQ325024	-	OQ349684	Khonsanit et al. (2024)
CQ05622	*Arachnidicola bashanensis*	OQ300411	OQ300421	OQ325025	-	OQ349685	Khonsanit et al. (2024)
CQ05921^T^	*Arachnidicola beibeiensis*	OQ300415	OQ300424	OQ325028	-	OQ349688	Khonsanit et al. (2024)
CQ05922	*Arachnidicola beibeiensis*	OQ300416	OQ300427	OQ325029	-	OQ349689	Khonsanit et al. (2024)
MST-FP3895^T^	*Arachnidicola carrolliae*	PX368950	PX352470	PX380463	-	PX380462	Tan et al. (2025)
RCEF7681^T^	*Arachnidicola anhuiensis*	PV134497	PV134556	PV166497	PV166551	PV166595	This study
MST-FP3877^T^	*Arachnidicola hookerae*	PX368951	PX353450	PX380465	-	PX380464	Tan et al. (2025)
NHJ 6709	*Arachnidicola kanyawimiae*	JN049865	EU369042	EU369025	EU369067	EU369086	Kepler et al. (2012)
TBRC 7243	*Arachnidicola kanyawimiae*	MF140750	MF140717	MF140837	MF140783	MF140807	Wang et al. (2024c)
TBRC 7242	*Arachnidicola kanyawimiae*	MF140751	MF140718	MF140838	MF140784	MF140808	Pu et al. (2025)
YFCC 1708939	*Arachnidicola kunmingensis*	OQ509521	OQ509508	OQ506284	OQ511533	OQ511547	Wang et al. (2024c)
YFCC 1808940^T^	*Arachnidicola kunmingensis*	OQ509522	OQ509509	OQ506285	OQ511534	OQ511548	Khonsanit et al. (2024)
ZY06511^T^	*Arachnidicola sinensis*	PV082711	PV082832	PV171231	-	PV171173	Chen et al. (2025c)
ZY06512	*Arachnidicola sinensis*	PV082712	PV082833	PV171232	-	PV171174	Chen et al. (2025c)
YFCC 2107937^T^	*Arachnidicola subaraneicola*	OQ509527	OQ509514	OQ506290	OQ511539	OQ511553	Pu et al. (2025)
YFCC 2107938	*Arachnidicola subaraneicola*	OQ509528	OQ509515	OQ506291	OQ511540	OQ511554	Khonsanit et al. (2024)
TBRC 7249	*Arachnidicola sulphurea*	MF140757	MF140721	MF140842	MF140786	MF140734	Khonsanit et al. (2024)
TBRC 7248^T^	*Arachnidicola sulphurea*	MF140758	MF140722	MF140843	MF140787	MF140812	Pu et al. (2025)
YFCC 1710936	*Arachnidicola sulphurea*	OQ509529	OQ509516	OQ506292	OQ511541	OQ511555	Wang et al. (2024c)
TBRC 7245^T^	*Arachnidicola thailandica*	MF140754	-	MF140839	-	MF140809	Pu et al. (2025)
KY11571^T^	*Arachnidicola tiankengensis*	ON502848	ON502825	ON525447	-	ON525446	Pu et al. (2025)
KY11572	*Arachnidicola tiankengensis*	ON502821	ON502827	ON525449	-	ON525448	Khonsanit et al. (2024)
TBRC 7251	*Arachnidicola waltergamsii*	MF140747	MF140713	MF140833	MF140781	MF140805	Khonsanit et al. (2024)
TBRC 7252^T^	*Arachnidicola waltergamsii*	MF140748	MF140714	MF140834	MF140782	MF140806	Pu et al. (2025)
YFCC 883	*Arachnidicola waltergamsii*	OQ509530	OQ509517	OQ506293	OQ511542	OQ511556	Wang et al. (2024c)
ZY06061^T^	*Arachnidicola zunyiensis*	PV082713	PV082834	PV171233	-	PV171175	Chen et al. (2025c)
ZY06062	*Arachnidicola zunyiensis*	PV082714	PV082835	PV171234	-	PV171176	Chen et al. (2025c)
BCC48975^T^	*Ascopolyporus albus*	OL331502	OL322048	OL322035	OL322056	OL322065	Thanakitpipattana et al. (2022)
BCC 48704^T^	*Ascopolyporus galloides*	OL331509	OL322044	OL322031	OL322055	OL322062	Thanakitpipattana et al. (2022)
BCC88430^T^	*Ascopolyporus purpuratus*	OL331506	OL322045	OL322032	OL322059	OL322063	Thanakitpipattana et al. (2022)
BCC 47541^T^	*Bhushaniella rubra*	OQ892128	OQ892133	OQ914428	OQ914431	OQ914433	Mongkolsamrit et al. (2023)
BCC 47542	*Bhushaniella rubra*	OQ892129	OQ892134	OQ914429	OQ914432	OQ914434	Mongkolsamrit et al. (2023)
DY101742	*Chlorocillium araneogenum*	MW730534	MW730619	MW753038	-	MW753031	Chen et al. (2025b)
MST F29447^T^	*Chlorocillium duiganiae*	PX353451	-	PX380469	-	PX380468	Tan et al. (2025)
RCEF6632	*Chlorocillium griseum*	MW031768	MW084342	MW091327	MW091331	MW091329	Chen et al. (2025b)
CBS 387.73^T^	*Chlorocillium griseum*	KU382150	KU382218	-	-	-	Chen et al. (2025b)
BRIP 72666a^T^	*Chlorocillium gueriniae*	OR750701	OR731507	OR737801	-	OR737790	Chen et al. (2025b)
DL10171^T^	*Chlorocillium guizhouense*	MN128448	-	MN101596	MN101595	-	Chen et al. (2025b)
DL10172	*Chlorocillium guizhouense*	PQ432742	-	PQ444210	MN101597	-	Chen et al. (2025b)
SD05362	*Chlorocillium lepidopterorum*	MW730611	MW730629	MW753042	-	-	Chen et al. (2025b)
SD05361^T^	*Chlorocillium lepidopterorum*	MW730543	MW730624	MW753041	-	-	Chen et al. (2025b)
MST-F26639	*Chlorocillium mauryae*	PQ607740	-	PQ566635	-	-	Tan et al. (2024)
MST-F26633^T^	*Chlorocillium mauryae*	PQ607739	-	PQ566634	-	PQ566633	Tan et al. (2024)
BRIP 70299a^T^	*Chlorocillium montefioreae*	PP420202	PP415875	PP438400	-	PP438395	Chen et al. (2025b)
DL10302	*Chlorocillium neorongjiangense*	MN165995	-	-	MN172268	MN172272	Chen et al. (2025c)
DL10221^T^	*Chlorocillium neolepidopterorum*	MN165990	-	MN172269	MN172267	-	Chen et al. (2025c)
KY07181^T^	*Chlorocillium sinense*	PP768154	PP768156	PP766580	-	PP766578	Chen et al. (2025b)
KY07182	*Chlorocillium sinense*	PP768155	PP768157	PP766581	-	PP766579	Chen et al. (2025b)
RCEF7509	*Chlorocillium* sp. XYC-2025d	PV134470	PV134529	PV166470	PV166541	-	This study
RCEF7510	*Chlorocillium* sp. XYC-2025d	PV134471	PV134530	PV166471	-	-	This study
DY09021^T^	*Chlorocillium vallense*	PQ432743	PQ432746	PQ444211	-	-	Chen et al. (2025b)
DY09022	*Chlorocillium vallense*	PQ432744	PQ432747	PQ444212	-	-	Chen et al. (2025b)
MST F3581^T^	*Chlorocillium winlockiae*	PQ607741	PQ607748	PQ566637	-	PQ566636	Tan et al. (2024)
BRIP 75891a	*Chlorocillium winlockiae*	PQ668640	PQ668637	PQ675575	-	PQ675574	Tan et al. (2024)
CBS 134.22^T^	*Cordyceps javanica*	MH854719	MG665231	MF416504	MF416661	MF416455	Chuang et al. (2024)
ARSEF 5413	*Cordyceps pruinosa*	JN049826	AY184968	DQ522351	DQ522397	DQ522451	Spatafora et al. (2007)
ARSEF 5135	*Cordyceps tenuipes*	AY624196	JF415980	JF416020	JN049896	JF416000	Dong et al. (2022b)
CBS797.84^T^	*Corniculantispora aranearum*	-	KM283787	KM283811	KM283833	KM283853	Khonsanit et al. (2024)
CBS345.37	*Corniculantispora dimorpha*	-	KM283788	KM283812	KM283834	KM283854	Khonsanit et al. (2024)
CBS363.86^T^	*Corniculantispora dimorpha*	-	AF339559	EF468784	EF468890	-	Khonsanit et al. (2024)
BRIP 72656a	*Corniculantispora margaretspencerae*	NR_191289	NG_242150	OR514841	-	OR514849	Tan and Shivas (2023c)
RCEF7796^T^	*Corniculantispora margaretspencerae*	PV134505	PV134564	PV166504	PV166554	-	This study
RCEF7877	*Corniculantispora margaretspencerae*	PV134516	PV134577	PV166518	PV166563	-	This study
CBS101270	*Corniculantispora psalliotae*	-	AF339558	EF469066	EF469095	EF469113	Khonsanit et al. (2024)
IMI 179841^T^	*Corniculantispora saksenae*	AJ292432	-	-	-	-	Zare et al. (2000)
GFRS14	*Corniculantispora saksenae*	MT447482	-	-	-	-	Yang et al. (2018)
CBS532.81	*Corniculantispora saksenae*	JN049846	AF339560	EF469067	EF469096	EF469112	Khonsanit et al. (2024)
CGMCC3.19304^T^	*Corpulentispora magnispora*	MK329102	MK329007	MK336037	-	MK335985	Zhang et al. (2021)
LC12469	*Corpulentispora magnispora*	MK329103	MK329008	MK336038	-	MK335986	Khonsanit et al. (2024)
OSC76404	*Drechmeria gunnii*	JN049822	AF339522	AY489616	AY489650	DQ522426	Xu et al. (2025)
CBS567.95^T^	*Drechmeria sinense*	AJ292417	AF339545	DQ522343	DQ522389	DQ522443	Spatafora et al. (2007)
CBS335.8^T^	*Drechmeria zeosporum*	NR_155046	AF339540	EF469062	EF469091	EF469109	Sung et al. (2007)
GZUIFRhuhu^T^	*Engyodontium huhutii*	MN944445	-	MT006068	MT006058	MT006063	Zhou et al. (2020)
IHEM 22910^T^	*Engyodontium parvisporum*	LC092896	LC092915	LC425558	-	-	Tsang et al. (2016)
CBS309.85^T^	*Engyodontium tenuipes*	JN036556	KM283802	DQ522341	KM283844	KM283866	Tsang et al. (2016)
BRIP 70251b^T^	* Fiorinimazzantia australiana *	-	OR947081	OR964979	-	OR964975	Tan and Shivas (2023d)
BRIP 72660a^T^	*Fiorinimazzantia elisabettae*	OR750703	OR731509	OR737803	-	OR737792	Tan and Shivas (2023d)
CBS 418.81^T^	*Flavocillium acerosum*	MH861361	KM283786	KM283810	KM283832	KM283852	Wang et al. (2020)
YFCC 6101^T^	*Flavocillium bifurcatum*	MN576833	MN576781	MN576951	MN576841	MN576897	Wang et al. (2020)
ARSEF 14694	*Flavocillium bifurcatum*	OR582994	OR577040	OR602802	OR602848	OR602852	Hajek et al. (2023)
CGMCC3.18987	*Gamszarea coprophilum*	MH177615	MH177618	MH184586	MH177621	MH177623	Khonsanit et al. (2024)
CGMCC3.18986^T^	*Gamszarea coprophilum*	MH177616	MH177619	MH184587	MH177622	MH177624	Khonsanit et al. (2024)
RCEF7802	*Gamszarella araneae*	PV134510	PV134569	PV166509	PV166557	-	This study
RCEF7803^T^	*Gamszarella araneae*	PV134511	PV134570	PV166510	PV166558	PV166602	This study
RCEF7804	*Gamszarella araneae*	PV134512	PV134571	PV166511	PV166559	PV166603	This study
DY05611^T^	*Gamszarella araneicola*	PV082731	PV082848	PV171251	PV171131	PV171187	Chen et al. (2025c)
DY05612	*Gamszarella araneicola*	PV082732	PV082849	PV171252	PV171132	PV171188	Chen et al. (2025c)
CBS 150062^T^	*Gamszarella buffelskloofina*	OR680769	OR717025	OR683715	-	OR683726	Crous et al. (2023)
COAD 3264	*Gamszarella buffelskloofina*	-	PQ962838	PV362929	PV362880	PV987267	Colmán et al. (2025)
DY09591^T^	*Gamszarella formicae*	PV082733	PV082850	PV171253	-	-	Chen et al. (2025c)
DY09592	*Gamszarella formicae*	PV082734	PV082851	PV171254	-	-	Chen et al. (2025c)
WD04111^T^	*Gamszarella pupicola*	PV082735	PV082852	PV171255	-	-	Chen et al. (2025c)
WD04112	*Gamszarella pupicola*	PV082736	PV082853	PV171256	-	-	Chen et al. (2025c)
WD04081^T^	*Gamszarella sinensis*	PQ527895	PQ527899	PQ553220	-	PQ553218	Crous et al. (2023)
WD04082	*Gamszarella sinensis*	PQ527896	PQ527900	PQ553221	-	PQ553219	Crous et al. (2023)
BRIP 72678a	*Gamszarella sotirae*	PP707901	PP707920	PP712788	-	-	Tan and Shivas (2024b)
BRIP 72673a^T^	*Gamszarella sotirae*	PP707902	PP707921	PP712789	-	PP712792	Tan and Shivas (2024b)
COAD 3263^T^	*Gamszarella uredinophila*	-	PQ962837	PV362928	PV362879	-	Colmán et al. (2025)
WD04101^T^	*Gamszarella vallensis*	PQ527897	PQ527901	-	-	-	Crous et al. (2023)
WD04102	*Gamszarella vallensis*	PQ527898	PQ527902	-	-	-	Crous et al. (2023)
A29	*Gibellula agroflorestalis*	-	PP958426	PP965287	-	-	(da Rocha Alves et al. 2025)
GNJ20210711-02^T^	*Gibellula alba*	-	OM090756	OM863559	-	-	This study
GNJ20210711-03	*Gibellula alba*	-	OM090757	OM863560	-	-	This study
IMI 507230^T^	*Gibellula attenboroughii*	-	PQ036929	PQ046101	-	-	Evans et al. (2025)
26PACOTI	*Gibellula aurea*	OK329885	-	OK392622	-	OK315663	Evans et al. (2025)
1PACOTI	*Gibellula aurea*	-	-	OK392618	-	OL117022	Evans et al. (2025)
BCC57817	*Gibellula brevistipitata*	OK040729	OK040706	OK040697	OK040715	-	Evans et al. (2025)
BCC39705^T^	*Gibellula cebrennini*	MH532874	MH394673	MH521895	MH521822	MH521859	Evans et al. (2025)
BCC53605	*Gibellula cebrennini*	MT477069	MT477062	MT503328	MT503321	MT503336	Evans et al. (2025)
ARSEF1915^T^	*Gibellula clavulifera* var. alba	JN049837	DQ518777	DQ522360	DQ522408	DQ522467	Evans et al. (2025)
LS20230802-66	*Gibellula flava*	-	-	PV166528	-	PV166612	This study
WFS20190625-25^T^	*Gibellula flava*	-	MW084343	MW091325	MW384883	-	Chen et al. (2021)
BCC45076	*Gibellula fusiformispora*	MH532882	-	-	MH521823	MH521860	Evans et al. (2025)
BCC56802^T^	*Gibellula fusiformispora*	MT477070	MT477063	MT503329	MT503322	MT503337	Evans et al. (2025)
BCC27968^T^	*Gibellula gamsii*	MH152529	MH152539	MH152560	MH152547	-	Evans et al. (2025)
BCC28797	*Gibellula gamsii*	MH152531	MH152541	MH152562	MH152549	MH152557	Evans et al. (2025)
RCEF8164	*Gibellula guizhouensis*	PX735924	PX735930	PX756795	PX756783	PX756790	This study
RCEF8165^T^	*Gibellula guizhouensis*	PX735925	PX735931	PX756796	PX756784	PX756791	This study
RCEF8166	*Gibellula guizhouensis*	PX735926	PX735932	PX756797	PX756785	PX756792	This study
RCEF8168	*Gibellula guizhouensis*	-	PX735933	PX756798	PX756786	PX756793	This study
RCEF8170	*Gibellula guizhouensis*	PX735927	PX735934	PX756799	PX756787	-	This study
BCC16025	*Gibellula leiopus*	-	MF416548	MF416492	MF416649	-	Evans et al. (2025)
BCC 40861	*Gibellula longicaudata*	OK040730	NG_088295	OK040698	OK040716	OK040724	Evans et al. (2025)
GNJ20200813-16	*Gibellula longispora*	-	-	MW961414	MW980145	-	Evans et al. (2025)
NHJ 12014^T^	*Gibellula longispora*	-	-	EU369017	EU369055	EU369075	Evans et al. (2025)
GNJ20210710-02	*Gibellula longispora*	-	OL854212	OL981628	-	OL981635	Evans et al. (2025)
NHJ7859	*Gibellula longispora*	-	-	-	EU369064	EU369085	Evans et al. (2025)
BCC 48888	*Gibellula parvula*	OK040731	OK040708	OK040699	OK040717	OK040725	Evans et al. (2025)
BCC49748^T^	*Gibellula parvula*	OK040732	OK040709	OK040700	OK040718	OK040726	Evans et al. (2025)
GNJ20200814-11	*Gibellula penicillioides*	MW969669	MW969661	MW961415	MZ215998	-	Evans et al. (2025)
GNJ20200814-14^T^	*Gibellula penicillioides*	MW969670	MW969662	MW961416	MZ215999	-	Evans et al. (2025)
BCC41203^T^	*Gibellula pigmentosinum*	MT477071	-	MT503330	MT503323	-	Evans et al. (2025)
BCC39707	*Gibellula pigmentosinum*	MH532875	MH394674	MH521894	MH521801	MH521856	Kuephadungphan et al. (2020)
BCC45580	*Gibellula pilosa*	OK040733	OK040710	OK040701	OK040719	-	Evans et al. (2025)
NHJ 10808	*Gibellula pulchra*	-	EU369035	EU369018	EU369056	EU369076	Evans et al. (2025)
BRIP 72767a^T^	*Gibellula queenslandica*	OR452099	OR452103	OR459912	-	OR459907	Tan and Shivas (2023a)
BCC47530	*Gibellula scorpioides*	MT477077	MT477065	MT503334	-	MT503338	Evans et al. (2025)
BCC47976^T^	*Gibellula scorpioides*	MT477078	MT477066	MT503335	MT503325	MT503339	Evans et al. (2025)
BCC45574^T^	*Gibellula solita*	OK040736	OK040712	OK040703	OK040721	-	Evans et al. (2025)
NHJ 11679	*Gibellula* sp.	-	-	EU369016	EU369054	-	Evans et al. (2025)
EPF081	*Gibellula* sp.	JX192723	JX192754	JX192818	-	-	Joseph et al. (2024)
EPF136	*Gibellula* sp.	JX192728	JX192759	JX192824	-	-	Joseph et al. (2024)
NHJ10788	*Gibellula* sp.	-	EU369036	EU369019	EU369058	EU369078	Evans et al. (2025)
NHJ5401	*Gibellula* sp.	-	-	-	EU369059	EU369079	Evans et al. (2025)
MT20211006-01	*Gibellula* sp. TW-2025a	OM842972	OM090755	OM863558	-	-	This study
RCEF7879	*Gibellula* sp. XYC-2025e	-	-	PV166520	-	PV166609	This study
LS20230802-73	*Gibellula* sp. XYC-2025f	-	-	PV166532	-	PV166616	This study
BCC36526^T^	*Gibellula trimorpha*	OK040737	-	OK040704	OK040722	OK040728	Evans et al. (2025)
BCC45112	*Gibellula unica*	OK040738	OK040713	OK040705	OK040723	-	Evans et al. (2025)
ARSEF5472^T^	*Harposporium harposporifera*	-	AF339519	DQ118747	DQ127238	-	Chen et al. (2025a)
ARSEF5354	*Harposporium helicoides*	-	AF339527	-	-	-	Chen et al. (2025a)
NHJ 10469	*Hevansia arachnophila*	-	EU369031	EU369008	EU369047	-	Johnson et al. (2009)
NHJ2465	*Hevansia arachnophila*	MH532899	-	MH521916	ON470205	ON470207	Mongkolsamrit et al. (2022)
NHJ2633	*Hevansia arachnophila*	MH532900	GQ249978	MH521917	MH521843	MH521884	Mongkolsamrit et al. (2022)
NHJ4314	*Hevansia cf. novoguineensis*	-	-	EU369012	EU369051	EU369071	Mongkolsamrit et al. (2022)
BCC2093	*Hevansia cf. novoguineensis*	-	MF416530	MF416473	-	MF416437	Mongkolsamrit et al. (2022)
BCC23860	*Hevansia cf. websteri*	GQ250009	GQ249979	GQ250030	-	-	Mongkolsamrit et al. (2022)
BCC36541	*Hevansia cf. websteri*	MH532868	MH394669	MH521889	MH521811	MH521849	Mongkolsamrit et al. (2022)
BRIP 62571a	*Hevansia mainiae*	-	-	OQ054475	-	OQ054474	Crous et al. (2023)
BRIP 62590a	*Hevansia mainiae*	-	-	OQ054479	-	OQ054478	Crous et al. (2023)
BCC47519^T^	*Hevansia minuta*	MZ684087	MZ684002	MZ707811	MZ707826	MZ707833	Mongkolsamrit et al. (2022)
BCC47520	*Hevansia minuta*	MZ684088	MZ684003	MZ707812	MZ707827	MZ707834	Mongkolsamrit et al. (2022)
BCC41864	*Hevansia nelumboides*	JN201871	JN201873	JN201867	-	-	Mongkolsamrit et al. (2022)
BCC 2190	*Hevansia nelumboides*	-	MF416531	MF416474	-	-	Mongkolsamrit et al. (2022)
CBS610.80^T^	*Hevansia novoguineensis*	MH532831	MH394646	MH521885	-	MH521844	Mongkolsamrit et al. (2022)
TNS16306	*Hevansia pseudonelumboides*	-	-	MF416475	-	MF416438	Kepler et al. (2017)
RCEF7270	*Hevansia pseudonelumboides*	-	PV134525	PV166464	-	PV166569	This study
RCEF7275^T^	*Hevansia pseudonelumboides*	-	PV134528	PV166469	PV166540	PV166571	This study
RCEF7801	*Hevansia pseudonelumboides*	PV134509	PV134568	PV166508	-	PV166601	This study
GNJ20210710-05	*Hevansia pseudonelumboides*	-	OM976882	ON326400	-	-	This study
GNJ20210710-08	*Hevansia pseudonelumboides*	-	-	ON326401	-	-	This study
GNJ20210711-04	*Hevansia pseudonelumboides*	-	OM976884	ON326402	-	-	This study
BRIP 74319a	*Husseyia annamariae*	PQ358381	PQ358383	-	-	-	Tan and Shivas (2023c)
BRIP 72654a^T^	*Husseyia annamariae*	OR750704	OR731510	OR737804	-	OR737793	Tan and Shivas (2023c)
BRIP 72669a^T^	*Husseyia queenslandica*	OR947074	OR947082	OR964980	-	OR964976	Tan and Shivas (2023c)
RCEF8222	*Husseyia sinensis*	PX735928	PX735935	PX756800	PX756788	PX756794	This study
RCEF8284^T^	*Husseyia sinensis*	PX735929	PX735936	PX756801	PX756789	-	This study
FJS20240724-94	*Husseyia* sp. XYC-2025g	-	-	PV166512	-	PV166604	This study
LS20230802-70	*Husseyia* sp. XYC-2025g	PV134520	-	PV166531	-	PV166615	This study
BCC02191	*Jenniferia cinerea*	MH532896	GQ249971	GQ250029	-	-	Mongkolsamrit et al. (2022)
NHJ 3510^T^	*Jenniferia cinerea*	-	-	EU369009	EU369048	EU369070	Mongkolsamrit et al. (2022)
BCC 42062^T^	*Jenniferia griseocinerea*	MZ684091	MZ684006	MZ707815	MZ707828	MZ707837	Mongkolsamrit et al. (2022)
BCC 42063	*Jenniferia griseocinerea*	MZ684092	MZ684007	MZ707816	MZ707829	MZ707838	Mongkolsamrit et al. (2022)
BCC 37881^T^	*Jenniferia thomisidarum*	MZ684099	MZ684010	MZ707823	MZ707830	MZ707843	Mongkolsamrit et al. (2022)
BCC 37882	*Jenniferia thomisidarum*	MZ684100	MZ684011	MZ707824	MZ707831	MZ707844	Mongkolsamrit et al. (2022)
HMAS 246915^T^	*Kanoksria zaquensis*	MT789699	MT789697	MT797812	MT797810	-	Khonsanit et al. (2024)
HMAS 246917	*Kanoksria zaquensis*	MT789698	MT789696	MT797811	MT797809	-	Khonsanit et al. (2024)
CBS164.70^T^	*Lecanicillium fusisporum*	AJ292428	KM283793	KM283817	KM283836	KM283858	Pu et al. (2025)
RCEF7795^T^	*Liangia guizhouensis*	PV134504	PV134563	PV166503	PV166553	PV166598	This study
YFCC 3103^T^	*Liangia sinensis*	MN576831	MN576782	MN576952	MN576842	MN576898	Wang et al. (2020)
YFCC 3104	*Liangia sinensis*	MN576832	MN576783	MN576953	MN576843	MN576899	Wang et al. (2020)
CBS 350.85^T^	*Neogamszarella antillana*	NR_111097	AF339536	DQ522350	DQ522396	DQ522450	Spatafora et al. (2007)
BCC39684	*Neotorrubiella chinghridicola*	MK632038	MK632096	MK632072	MK632181	MK632148	Thanakitpipattana et al. (2022)
BCC80733^T^	*Neotorrubiella chinghridicola*	MK632039	MK632097	-	MK632176	MK632149	Thanakitpipattana et al. (2022)
BCC83025^T^	*Niveomyces albus*	-	ON103157	ON125015	ON286876	ON125027	Pu et al. (2025)
CBS 756.73^T^	*Niveomyces insectorum*	MH860798	ON103169	ON125026	ON286887	ON125038	Pu et al. (2025)
BRIP 63050a	*Niveomyces multisynnematus*	OR335770	OR304276	OR335711	-	OR335710	Pu et al. (2025)
BTCC F35	*Nuciformispora araneae*	AB378506	-	-	-	-	Sukarno et al. (2009)
NBRC 105407^T^	*Nuciformispora araneae*	NR_121208	-	-	-	-	Schoch et al. (2014)
CBS726.73a^T^	*Nuciformispora aranearum*	AJ292464	AF339537	EF468781	EF468887	EF468934	Zare et al. (2000)
XY07011^T^	*Nuciformispora araneicola*	PV082745	PV082862	PV171265	-	-	Chen et al. (2025c)
XY07012	*Nuciformispora araneicola*	PV082746	PV082863	PV171266	-	-	Chen et al. (2025c)
IRAN 3689C^T^	*Nuciformispora rasoulzarei*	OR339890	-	OR352917	-	-	Armand et al. (2024)
DY05281^T^	*Nuciformispora sinense*	PV082747	PV082864	PV171267	PV171139	-	Chen et al. (2025c)
DY05282	*Nuciformispora sinense*	PV082748	PV082865	PV171268	PV171140	-	Chen et al. (2025c)
S01	*Nuciformispora spenceae*	PP587400	-	-	-	-	Unpublished
BRIP 72646a^T^	*Nuciformispora spenceae*	NR_191329	-	OR737798	-	OR737787	Tan and Shivas (2023c)
DY09691^T^	*Nuciformispora vallise*	PV082749	PV082866	PV171269	PV171141	-	Chen et al. (2025c)
DY09692	*Nuciformispora vallise*	PV082750	PV082867	PV171270	PV171142	-	Chen et al. (2025c)
SY09221^T^	*Nuciformispora zunyiense*	PV082751	PV082868	PV171271	PV171143	PV171193	Chen et al. (2025c)
SY09222	*Nuciformispora zunyiense*	PV082751	PV082868	PV171271	PV171143	PV171193	Chen et al. (2025c)
HUA 186135^T^	*Ophiocordyceps araracuarensis*	JN943325	JN941412	AB968578	JN992463	AB968540	Ban et al. (2015b)
BCC36938^T^	*Ophiocordyceps campes*	MT783955	MT118175	MT118167	MT118183	MT118188	Tasanathai et al. (2020)
GACP FG21042850	*Ophiocordyceps fenggangensis*	OR527534	OR527541	OR526345	OR526350	OR526353	Peng et al. (2024)
EFCC 7287	*Ophiocordyceps sinensis*	JN049854	EF468827	EF468767	EF468874	EF468924	Tang et al. (2023)
MFLU 22-0260	*Orbiocrella petchii*	OQ127339	OQ127374	OQ186366	OQ186420	OQ186396	Dong et al. (2022a)
BRIP 72613a^T^	*Orbiocrella zlotorzyckae*	OR527522	OR527532	OR514847	-	OR514855	Tan and Shivas (2023b)
NBRC 108993^T^	*Paraisaria coenomyiae*	AB968396	AB968412	AB968570	-	AB968532	Tehan et al. (2023)
EFCC 10125	*Paraisaria heteropoda*	JN049852	EF468812	EF468752	EF468860	EF468914	Tehan et al. (2023)
ZY 22.006^T^	*Paraneoaraneomyces sinensis*	OQ709254	OQ709260	OQ719626	-	OQ719621	Zhang et al. (2023)
ZY 22.008	*Paraneoaraneomyces sinensis*	OQ709256	OQ709262	OQ719628	-	OQ719623	Zhang et al. (2023)
MFLU 17-1394	*Pleurocordyceps aurantiaca*	MG136918	MG136912	MG136876	MG136867	MG136872	Xiao et al. (2023)
MFLU 21-0275^T^	*Pleurocordyceps nutansis*	OQ172073	OQ172048	OQ459739	OQ459765	OQ459811	Xiao et al. (2023)
KY07231^T^	*Purpureocillium aranea*	PV082801	PV082918	PV171319	-	-	Chen et al. (2025c)
KY07232	*Purpureocillium aranea*	PV082802	PV082919	PV171320	-	-	Chen et al. (2025c)
RCEF7731^T^	*Purpureocillium araneicola*	PV134503	PV134562	PV166502	PV166552	PV166597	This study
NL20210822-14	*Purpureocillium araneicola*	-	-	OM482405	-	-	This study
GNJ20200814-05	*Purpureocillium araneicola*	OM976851	-	ON326409	ON326410	-	This study
FLOR73173^T^	*Purpureocillium atlanticum*	-	PX571243	PX548866	-	-	Araújo et al. (2025)
CBS744.73	*Purpureocillium atypicola*	-	EF468841	EF468786	EF468892	-	Sung et al. (2007)
OSC151901	*Purpureocillium atypicola*	-	KJ878880	KJ878961	KJ878994	-	Quandt et al. (2014)
RCEF7274	*Purpureocillium atypicola*	PV134469	PV134527	PV166468	-	-	Chang et al. (2024b)
KUNCC23-13355	*Purpureocillium atypicola*	OR910610	OR910613	OR920384	OR920387	-	Yang et al. (2024)
MFLUCC 24-0325^T^	*Purpureocillium hyophilae*	PQ453485	PQ453486	PQ480033	-	-	Gunarathne et al. (2025)
JX17D04	*Purpureocillium jiangxiense*	PP555636	PP555645	PP658209	-	-	Chen et al. (2024c)
FMR10376^T^	*Purpureocillium lavendulum*	FR734106	FR775489	FR775516	FR775512	FR775538	Chen et al. (2024b)
FMR10452	*Purpureocillium lavendulum*	-	FR775490	FR775517	FR775513	FR775539	Perdomo et al. (2013)
CBS284.36^T^	*Purpureocillium lilacinum*	MH855800	FR775484	EF468792	EF468898	EF468941	Vu et al. (2019)
IOM325363.1	*Purpureocillium roseum*	MT560195	MT560197	-	-	-	Calvillo‐Medina et al. (2021)
IOM325363.2	*Purpureocillium roseum*	MT560196	MT560198	-	-	-	Calvillo‐Medina et al. (2021)
TR05091^T^	*Purpureocillium sinense*	PV082803	PV082920	PV171321	-	-	Chen et al. (2025c)
TR05092	*Purpureocillium sinense*	PV082804	PV082921	PV171322	-	-	Chen et al. (2025c)
JS19C02	*Purpureocillium sodanum*	PP384995	PP381049	-	-	-	Hyde et al. (2016)
NHJ3582	*Purpureocillium takamizusanense*	-	EU369034	EU369015	-	-	Johnson et al. (2009)
BCC66194	*Purpureocillium takamizusanense*	MH532888	MH394682	MH521915	MH521842	MH521883	Kuephadungphan et al. (2014)
RCEF4811	*Purpureocillium takamizusanense*	MT568626	MW718253	MT583722	MW723137	MW723158	Lin et al. (2023)
KY07471^T^	*Purpureocillium tiankengense*	PV082805	PV082922	PV171323	-	-	Chen et al. (2025c)
KY07472	*Purpureocillium tiankengense*	PV082806	PV082923	PV171324	-	-	Chen et al. (2025c)
TK041^T^	*Purpureocillium zongqii*	PQ211280	PQ211284	PQ223681	-	-	Chen et al. (2024b)
TK042	*Purpureocillium zongqii*	PQ211281	PQ211285	PQ223682	-	-	Chen et al. (2024b)
CBS 101437	*Rotiferophthora angustispora*	OR600291	AF339535	-	DQ522402	DQ522460	Spatafora et al. (2007)
RCEF4111	*Rotiferophthora angustispora*	GU108582	-	KP324765	-	KP324768	Chen et al. (2011)
CBS 704.86^T^	*Simplicillium lanosoniveum*	AJ292396	AF339553	DQ522358	DQ522406	DQ522464	Spatafora et al. (2007)
BCRC 34536^T^	*Simplicillium salviniae*	MT974200	MT974415	MW200240	MW200244	MW200248	Chuang et al. (2024)
OSC71233	*Tolypocladium capitata*	-	AY489721	AY489615	AY489649	DQ522421	Spatafora et al. (2007)
OSC110990	*Tolypocladium fractum*	-	DQ518759	DQ522328	DQ522373	DQ522425	Spatafora et al. (2007)
OSC110991	*Tolypocladium japonica*	JN049824	DQ518761	DQ522330	DQ522375	DQ522428	Spatafora et al. (2007)
OSC106405	*Tolypocladium ophioglossoides*	-	AY489723	AY489618	AY489652	DQ522429	Spatafora et al. (2007)
GZUIFRZHJ01^T^	*Zouia cauligalbarum*	MH730663	MH730667	MH801920	MH801922	MH801924	Khonsanit et al. (2024)
GZUIFRZHJ02	*Zouia cauligalbarum*	MH730664	MH730668	MH801921	MH801923	MH801925	Khonsanit et al. (2024)

### Divergence time estimation

The dataset used for divergence time estimation was constructed using five nuclear loci (ITS, nrLSU, *TEF1*, *RPB1* and *RPB2*) following the general framework of Wei et al. (2022). One fossil calibration and one secondary calibration point were applied to constrain node ages. Specifically, the fossil *Paleoophiocordyceps coccophagus* (Sung et al. 2008) was used to calibrate the crown node of *Hirsutella*, *Ophiocordyceps* and *Paraisaria*, using an exponential prior (offset = 100 Ma, mean = 27.5), with the 95% credibility interval following estimates reported in Wei et al. (2022). The root height was secondarily calibrated based on the divergence between *Leotiomycetes* and *Sordariomycetes*, using a normal prior (mean = 258, SD = 35), as adopted from Samarakoon et al. (2016). Molecular clock analyses were performed using BEAST v1.8.4 (Drummond et al. 2012). The dating dataset was independent from the multilocus alignment used for taxonomic phylogenetic analyses and was assembled specifically for divergence time estimation (GenBank accession numbers listed in Suppl. material 2). In general, a single representative strain per species was selected, preferentially ex-type strains or strains with the most complete sequence information. For spider-pathogenic fungi and closely related taxa within *Cordycipitaceae*, *Clavicipitaceae* and *Ophiocordycipitaceae*, sampling aimed to include most described genera. For other hypocrealean lineages, representative taxa were selected based on sequence completeness and major ecological traits, in order to achieve a taxonomically comprehensive but proportionally scaled dataset suitable for time calibration. The concatenated alignment was partitioned by locus and imported into BEAUti v1.8.4 to generate the XML configuration. Substitution models were set to be unlinked, while the clock and the tree prior parameters were set as linked across partitions. An uncorrelated relaxed clock model with a lognormal distribution of rates was applied to each partition (Drummond et al. 2006). Speciation was modelled using a Yule prior, following Wei et al. (2022), acknowledging that this pure-birth model assumes constant diversification rates and no extinction. The BEAST analysis was run for 100 million generations, with parameters sampled every 1,000 generations and four independent runs were performed in parallel. Tracer v1.7.2 was used to assess convergence and evaluate the effective sample size (ESS) of each run. Posterior sampling was considered sufficient when ESS values for all parameters in the combined runs were ≥ 200. The first 20% of trees from each run were discarded as burn-in and the remaining trees were combined using LogCombiner v1.8.4. The maximum clade credibility (MCC) tree was generated using TreeAnnotator v1.8.0. FigTree v1.4.4 (http://tree.bio.ed.ac.uk/software/figtree/) was used to visualize the resulting tree and to extract the mean node ages and their 95% highest posterior density (HPD) intervals (Drummond and Rambaut 2007). Posterior probability (PP) values equal to or greater than 0.95 were considered to indicate significant support. Geological timescales were based on the International Chronostratigraphic Chart (Cohen et al. 2025).

## Results

### Phylogenetic analyses

Analysis 1: Phylogenetic relationships within *Cordycipitaceae*

**Figure 1.**
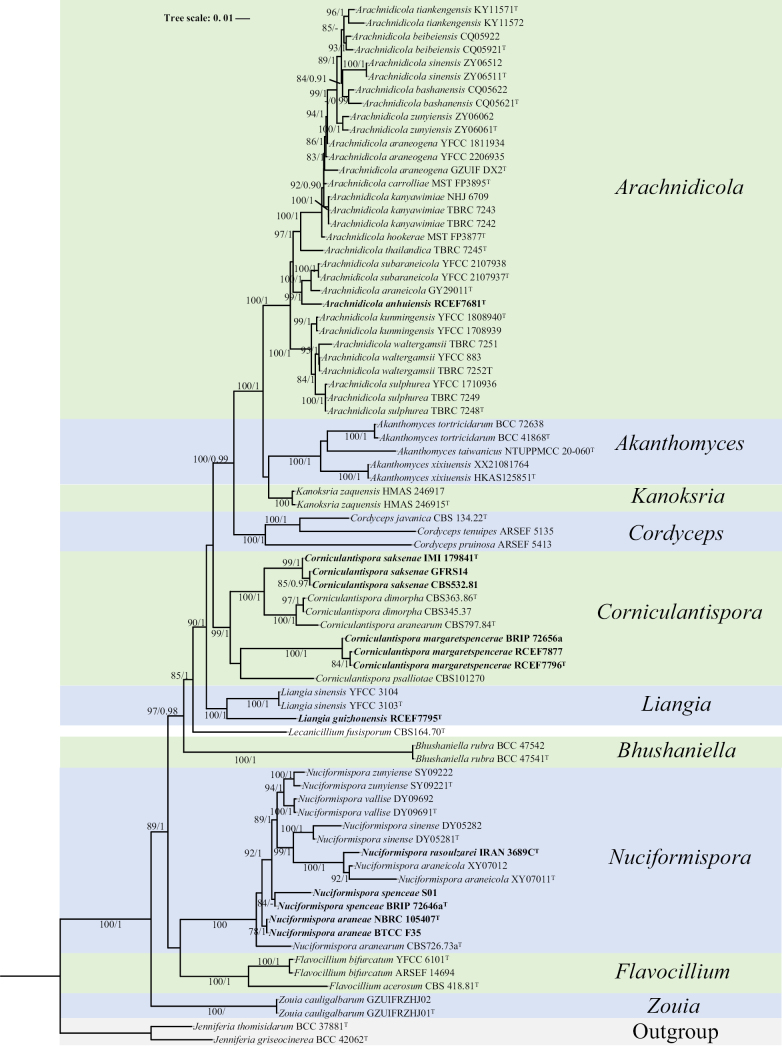
Phylogram retrieved from IQ-TREE based on the combined dataset of five loci (ITS, nrLSU, *TEF1*, *RPB1*, and *RPB2*), showing the phylogenetic relationships of *Arachnidicola*, *Liangia*, and related species in *Cordycipitaceae*. Statistical support values are provided at nodes as MLBS/BPP (only MLBS ≥ 75% and BPP ≥ 0.90 are shown). New taxa are highlighted in bold. ^T^ = ex-type culture.

Phylogenetic inference for the family *Cordycipitaceae* was performed to resolve the taxonomic placement of the newly collected isolates (Fig. 1). *Jenniferia griseocinerea* BCC 42062 and *J. thomisidarum* BCC 37881 served as the outgroup taxa in the study. The dataset included 78 taxa and consisted of 4,349 (ITS, 599, nrLSU, 894, *TEF1*, 1005, *RPB1*, 735 and *RPB2*, 1116) characters with gaps. The selected model for ML analysis was TIM+F+I+R3. The maximum likelihood (ML) and Bayesian inference (BI) methods produced highly concordant phylogenetic trees (Fig. 1), with strong statistical support for most clades. The majority of genera formed distinct monophyletic groups. The new strains were grouped into the genera *Arachnidicola*, *Corniculantispora* and *Liangia*.

#### Analysis 2: Phylogeny of *Gamszarella*, *Gibellula*, and *Hevansia*

**Figure 2.**
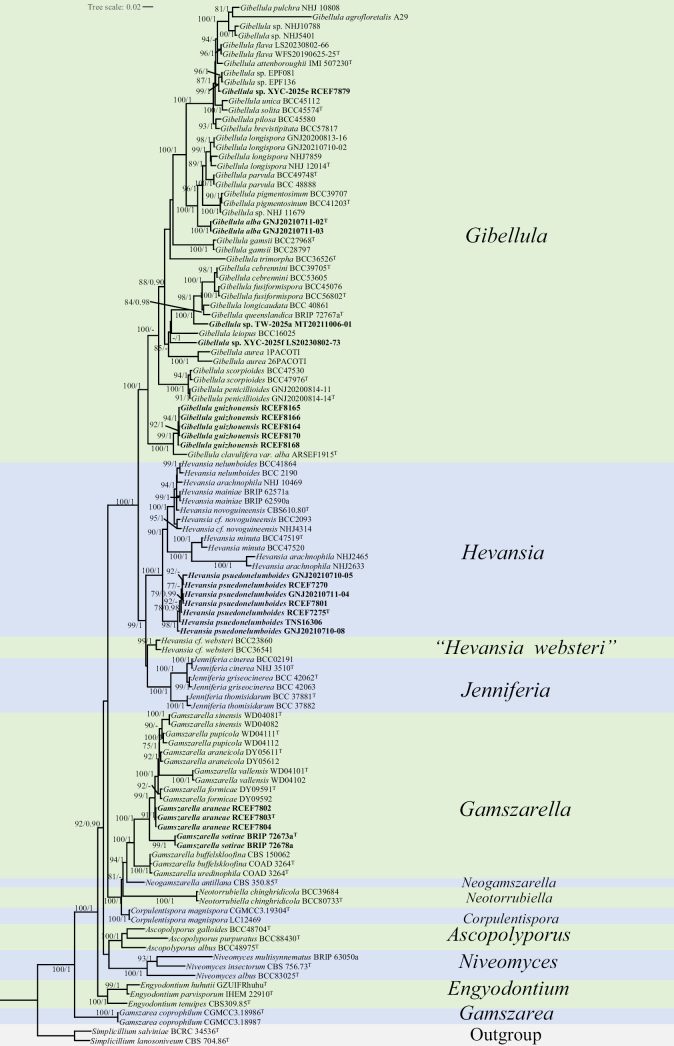
Phylogram retrieved from IQ-TREE based on the combined dataset of five loci (ITS, nrLSU, *TEF1*, *RPB1*, and *RPB2*), showing the phylogenetic relationships of *Gibellula*, *Hevansia*, *Gamszarella*, and related species in *Cordycipitaceae*. Statistical support values are provided at nodes as MLBS/BPP (only MLBS ≥ 75% and BPP ≥ 0.90 are shown). New taxa are highlighted in bold. ^T^ = ex-type culture.

The second analysis focused on the phylogenetic positions of new isolates within the genera *Gamszarella*, *Gibellula*, and *Hevansia* (Fig. 2). *Simplicillium lanosoniveum* CBS 704.86 and *S. salviniae* BCRC 34536 served as the outgroup taxa in the study. The dataset included 112 taxa and consisted of 4,316 (ITS, 613, nrLSU, 876, *TEF1*, 969, *RPB1*, 751 and *RPB2*, 1106) characters with gaps. The selected model for ML analysis was TIMe+I+G4. The maximum likelihood (ML) and Bayesian inference (BI) methods produced highly concordant phylogenetic trees (Fig. 2), with strong statistical support for most clades. The majority of genera formed distinct monophyletic groups. The new strains were grouped into the genera *Gamszarella*, *Gibellula* and *Hevansia*. In addition, it is noteworthy that, consistent with the phylogenetic results of Evans et al. (2025) and Hongsanan et al. (2025), *Hevansia cf. websteri* does not cluster with other members of *Hevansia*, but instead forms an independent lineage. The phylogenetic placement of *H. cf. websteri* therefore remains uncertain and warrants further investigation.

#### Analysis 3: Phylogeny of *Clavicipitaceae* and *Ophiocordyceps*

**Figure 3.**
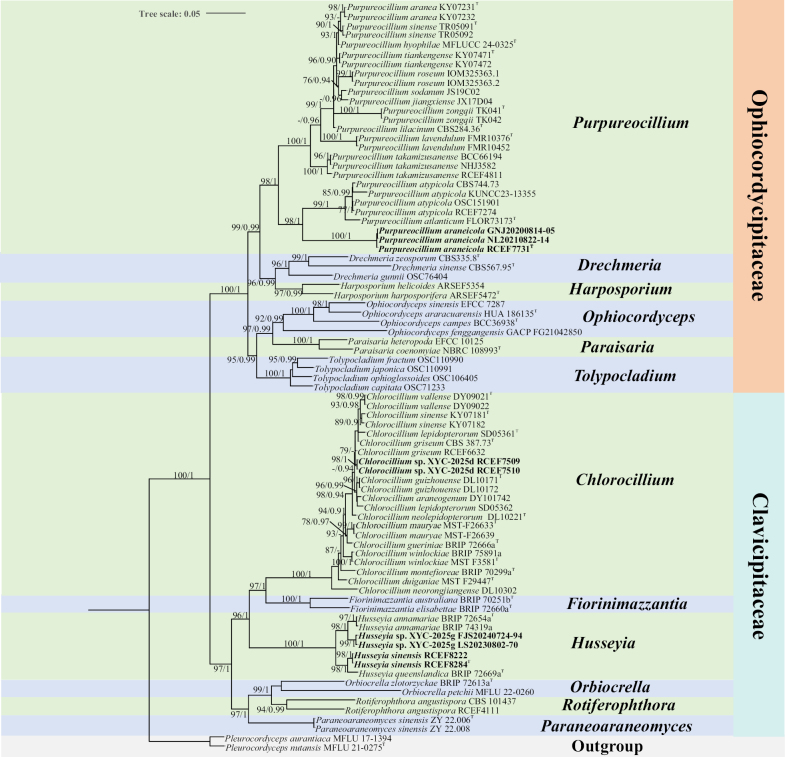
Phylogram retrieved from IQ-TREE based on the combined dataset of five loci (ITS, nrLSU, *TEF1*, *RPB1*, and *RPB2*), showing the phylogenetic relationships of *Clavicipitaceae* and *Ophiocordycipitaceae*. Statistical support values are provided at nodes as MLBS/BPP (only MLBS ≥ 75% and BPP ≥ 0.90 are shown). New taxa are highlighted in bold. ^T^ = ex-type culture.

The third analysis was conducted to establish the taxonomic status of the new species within *Clavicipitaceae* and *Ophiocordycipitaceae* (Fig. 3). *Pleurocordyceps aurantiaca* MFLU 17-1394 and *P. nutansis* MFLU 21-0275 served as the outgroup taxa in the study. The dataset included 81 taxa and consisted of 3,952 (ITS, 532, nrLSU, 852, *TEF1*, 909, *RPB1*, 681 and *RPB2*, 978) characters with gaps. The selected model for ML analysis was TIM3+F+R4. The maximum likelihood (ML) and Bayesian inference (BI) methods produced highly concordant phylogenetic trees (Fig. 3), with strong statistical support for most clades. The majority of genera formed distinct monophyletic groups. The new strains were grouped into the genera *Chlorocillium*, *Husseyia* and *Purpureocillium*.

The initial phylogenetic analyses based on five loci indicated that several isolates in this study may represent eight putative new species. To further delimit and confirm these species, we conducted additional analyses based on the principle of genealogical concordance phylogenetic species recognition (GCPSR), which requires concordance among at least three independent, unlinked loci and helps to overcome limitations inherent to single-tree phylogenetic species delimitation (Taylor et al. 2000). For the putative new species, single-locus phylogenetic trees were reconstructed separately based on rDNA (ITS+nrLSU), *TEF1*, *RPB1* and *RPB2* (Suppl. material 3). In all newly proposed species, at least three single-locus phylogenies provided concordant and independent support for their recognition as distinct lineages. When combined with diagnostic morphological evidence, these results collectively support the establishment and recognition of the new taxonomic entities described in the subsequent taxonomic treatment.

Furthermore, it is worth mentioning that strains RCEF7879, LS20230802-73 and MT20211006-01 form three well-supported monophyletic clades within *Gibellula*ML bootstrap support (MLBS) = 98%, Bayesian posterior probability (BPP) = 1.00; MLBS = 84, BPP = 0.9, each representing distinct evolutionary lineages. Strains RCEF7509 and RCEF7510 cluster as a sister group within the genus *Chlorocillium*, forming a distinct monophyletic branch (MLBS = 80, BPP = 1.0). Similarly, strains LS20230802-70 and FJS20240724-94 constitute a well-supported clade within *Husseyia* (MLBS = 99, BPP = 1.0). Based on molecular phylogenetic evidence, these strains represent putative novel species. However, owing to incomplete morphological characterization, they are provisionally designated as *Gibellula* sp. XYC-2025e, *Gibellula* sp. XYC-2025f, *Gibellula* sp. TW-2025a, *Chlorocillium* sp. XYC-2025d, and *Husseyia* sp. XYC-2025g. Detailed taxonomic treatment of these taxa is deferred pending comprehensive morphological analysis. Furthermore, the phylogenetic framework provides robust support for the placement of *Chlorocillium* and *Husseyia* within the family *Clavicipitaceae*, corroborating their taxonomic assignment in this family.

### Taxonomy

#### Arachnidicola anhuiensis

Taxon classification

Animalia

HypocrealesClavicipitaceae

X.Y. Chang & M.J. Chen sp. nov.

D58E47E5-858A-59DA-974D-F9C2C7AEE849

858516

Fig. 4

##### Etymology.

Name refers to “Anhui Province” where the holotype was collected.

**Figure 4.**
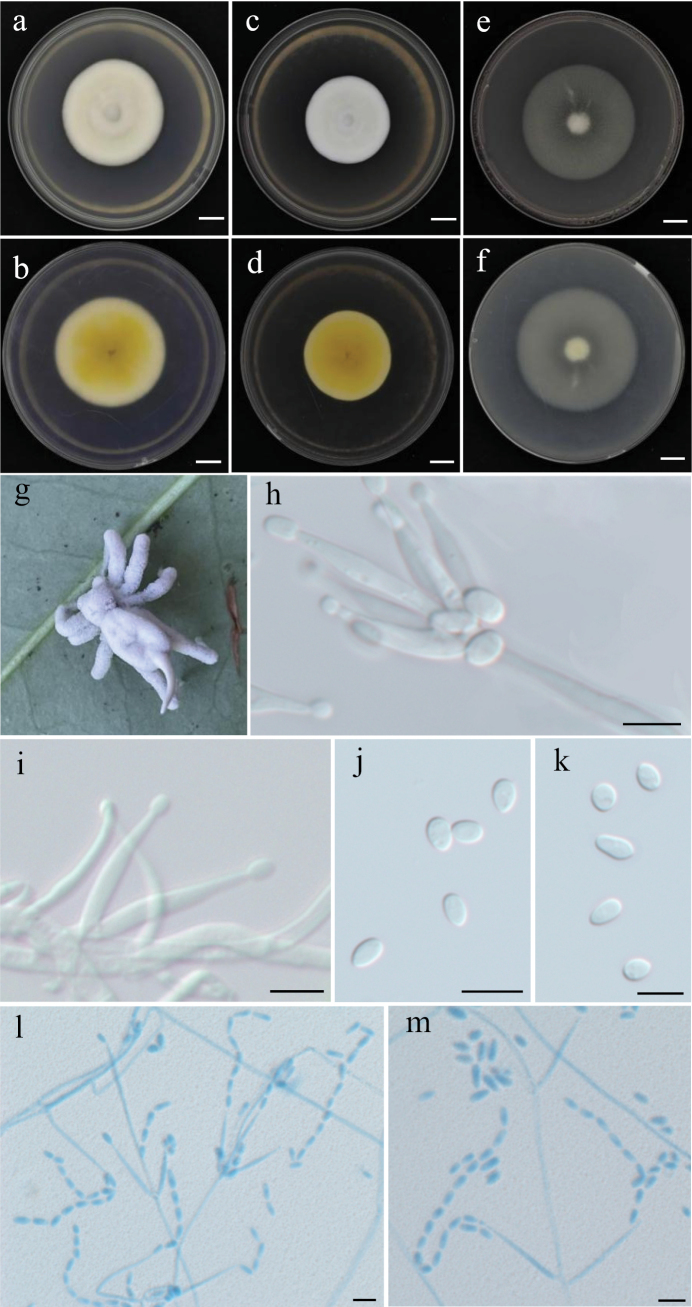
Holotype specimen and microscopic characteristics of *A. anhuiensis*. **a, b**. Colony obverse and reverse on PDA; **c, d**. Colony obverse and reverse on MEA; **e, f**. Colony obverse and reverse on OA; **g**. Infected host spider; **h, i**. Conidiogenous structures and conidia on the specimen; **j, k**. Mature conidia; **l, m**. Conidiogenous structures. Scale bars: 10 mm (**a–f**); 5 um (**h–m**).

##### Typification.

China • Anhui Province, Huangshan City, Qimen County, Guniujiang Scenic Area (30°04'N, 117°34'E), on dead spiders (*Araneae*), July 2024; Xiaoyun Chang and Mingjun Chen, holotype GNJ20240708-06, ex-type RCEF7681.

##### Description.

On the host spider, a dense mycelium ranging from white to purpleish-white completely covers the body. Hyphae septate, hyaline, smooth-walled, 1.0–2.5 μm wide. Conidiophores mononematous, hyaline, smooth-walled, with single phialide or whorls of 2–6 phialides, or penicillium-like from hyphae directly. Phialides slightly swollen at the base, cylindrical, 10.2–28.8 × 1.0–2.3 μm (x̄ = 16.7 ± 5.0 × 1.6 ± 0.4 μm), gradually tapering into a narrow neck. Conidia hyaline, smooth-walled, predominantly elliptical, ovoid or fusiform, 3.0–6.2 × 2.0–5.6 μm (x̄ = 4.0 ± 0.8 × 2.7 ± 0.6 μm), forming divergent and basipetal chains. The shape and size of phialides and conidia in culture conditions were similar to those on spiders.

##### Culture characteristics.

On PDA, colonies are white to pale yellow on the front side and pale yellow to yellowish brown on the reverse, with lighter-colored margins. Mycelium is dense and cottony, with slow growth, reaching 40–44 mm in diameter after 14 days at 25 °C. On MEA, colonies are similar to those on PDA but exhibit slower growth and deeper yellow coloration on the reverse. On OA, colonies form only a thin, white mycelial layer with weak growth.

##### Host.

spider (*Araneae*).

##### Note.

Khonsanit et al. (2024) re-evaluated the phylogenetic framework of the genus *Akanthomyces* and resolved it into four well-supported monophyletic lineages: *Akanthomyces*, *Arachnidicola*, *Lecanicillium* and *Kanoksria*, each exhibiting distinct host preferences and asexual morphologies. *Arachnidicola* is exclusively associated with spiders with most species producing *Isaria*-like synnemata. In this study, an *Isaria*-like spider-pathogenic fungus was collected from Anhui Province, China, and identified as a new species, *A. anhuiensis*, based on combined five-locus phylogeny and morphology. Phylogenetic analyses have confirmed that *A. anhuiensis* forms a distinct and well-supported clade (MLBS = 99, BPP = 1.0), indicating a close evolutionary relationship with *A. araneicola* and *A. subaraneicola*. In addition, the single-locus phylogenies based on rDNA (ITS+nrLSU), *TEF1*, *RPB1* and *RPB2* showed genealogical concordance, each supporting the monophyly of *A. anhuiensis* (Suppl. material 3: fig. S2), consistent with the criteria of genealogical concordance phylogenetic species recognition (GCPSR). A BLASTN search against the GenBank database revealed significant sequence differences between *A. anhuiensis* and the type strains of these two species. Specifically, *A. anhuiensis* showed 96.66% identity for ITS, 98.30% for *TEF1*, 97.80% for *RPB1* and 98.67% for *RPB2* compared to *A. araneicola* GY29011. In comparison to *A. subaraneicola* YFCC 2107937, *A. anhuiensis* exhibited 97.27% identity for ITS, 98.83% for nrLSU, 97.70% for *TEF1*, 97.38% for *RPB1* and 98.67% for *RPB2*. Morphologically, *A. anhuiensis* also resembles both species, especially in its white to pale yellow mycelium.

Nonetheless, significant differences were observed in conidial morphology and size: *A. anhuiensis* produces larger ellipsoidal to ovoid conidia (3.0–6.2 × 2.0–5.6 μm), compared to the smaller fusiform conidia of *A. araneicola* (2.5–5.0 × 1.3–1.9 μm) (Chen et al. 2019) and the slightly narrower ellipsoidal to elongate-ellipsoidal conidia of *A. subaraneicola* (3.0–5.4 × 1.8–3.4 μm) (Wang et al. 2024c). This represents the first newly described species of *Arachnidicola* since the genus was established, highlighting the taxonomic value of host specificity and subtle morphological characters in spider-associated fungi.

#### Gamszarella araneae

Taxon classification

Animalia

HypocrealesClavicipitaceae

X.Y. Chang & M.J. Chen sp. nov.

1C1E3E83-7FE2-5CC0-9FE2-C64637956DFD

858517

Fig. 5

##### Etymology.

referring to the ability to colonize spider.

**Figure 5.**
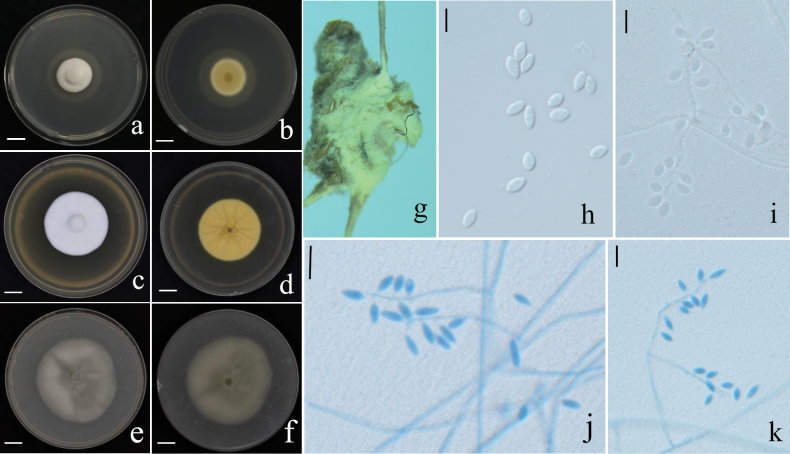
Holotype specimen and microscopic characteristics of *G. araneicola*. **a, b**. Colony obverse and reverse on PDA; **c, d**. Colony obverse and reverse on MEA; **e, f**. Colony obverse and reverse on OA; **g**. Infected host spider; **h**. Mature conidia; **i–k**. Conidiogenous structures and conidia. Scale bars: 10 mm (**a–f**); 5 um (**g–k**).

##### Typification.

China • Guizhou Province, Tongren City, Fanjingshan National Nature Reserve (27°50'N, 108°47'E), on dead spiders (*Araneae*), July 2024; Xiaoyun Chang and Mingjun Chen, holotype FJS20240724-93, ex-type RCEF7803.

##### Description.

On the host spider, the body is completely covered by a dense, pale yellow mycelium. Hyphae hyaline, smooth-walled, septate, and branched, 0.9–1.7 μm wide. Conidiophores erect, usually arising from aerial hyphae, solitary or resembling the *Lecanicillium* type. Conidiogenous cells solitary or arranged in whorls of 2–3, cylindrical at the base with multiple small denticles, gradually narrowing into a distinct neck, 11.7–31.7(–40.5) × 0.9–1.5 μm (x̄ = 22.8 ± 6.4 × 1.1 ± 0.2 μm). Conidia hyaline, elliptical to fusiform, single-celled, (2.7–)3.5–5.3(–7.0) × 1.8–3.5 μm (x̄ = 4.2 ± 0.7 × 2.7 ± 0.4 μm). The shape and size of phialides and conidia in culture conditions were similar to those on spiders.

##### Culture characteristics.

On PDA, colonies are circular with smooth margins and velvety mycelium, exhibiting a distinct concentric growth pattern. The surface is white, while the reverse is pale yellow to light yellowish brown, with lighter margins gradually fading into a translucent halo. Colony diameter reaches approximately 34 mm after 14 days at 25 °C. On MEA, colonies are white on the surface, pale yellow on the reverse, and display prominent radial cracks. On OA, colonies are white to greyish white with thin and sparse mycelium, and exhibit weak growth.

##### Host.

spider (*Araneae*).

##### Additional material examined.

China • Guizhou Province, Tongren City, Fanjingshan National Nature Reserve (27°50'N, 108°47'E), on dead spiders, July 2024; Xiaoyun Chang and Mingjun Chen, RCEF7802 and RCEF7804 (living culture).

##### Note.

Multilocus phylogenetic analyses confirmed that *Gamszarella araneae* forms a strongly supported independent clade (MLBS = 91, BPP = 1.0). Single-locus phylogenetic trees based on rDNA (ITS+nrLSU), *RPB1* and *RPB2* consistently resolved *G. araneae* as a distinct lineage from its closest relatives, in accordance with the GCPSR criterion (Suppl. material 3: fig. S3).

Morphologically, *G. araneae* shares several characters with related species of the genus, and some measurements overlap with those reported for closely related taxa (Table 2). Nevertheless, it can be differentiated from *G. araneicola* by its narrower conidiogenous cells [11.7–31.7(–40.5) × 0.9–1.5 μm vs 27.7–36.9 × 1.5–1.9 μm]. It differs from *G. formicae* by having larger conidia [elliptical to fusiform, (2.7–)3.5–5.3(–7.0) × 1.8–3.5 μm vs fusiform to ellipsoidal, 2.2–3.8 × 1.7–2.5 μm] and by its spider host. It can be distinguished from *G. pupicola* by its longer conidiogenous cells (11.7–31.7(–40.5) × 0.9–1.5 μm vs 10.0–23.4 × 0.7–1.0 μm) and host association, as *G. pupicola* parasitizes lepidopteran pupae.

**Table 2.** Morphological comparison of the new species with other *Gamszarella* species.

Species	Colony morphology (on PDA at 25 °C)	Conidiogenous cells (μm)	Conidia (μm)	Host	References
*G. araneae*	White, reverse pale yellow, reaching 34 mm diam.	Numerous denticles, 11.7–31.7(–40.5) × 0.9–1.5	Elliptical to fusiform, (2.7–)3.5–5.3(–7.0) × 1.8–3.5	Spider	This study
*G. araneicola*	White, reverse yellowish, reaching 45–47 mm diam.	Numerous denticles, 27.7–36.9 × 1.5–1.9	Fusiform to ellipsoidal, 3.5–6.0 × 2.1–2.3	Spider	Chen et al. (2025c)
*G. formicae*	White, reverse yellowish, reaching 43–44 mm diam.	Numerous denticles, 13.7–21.3 × 1.3–2.3	Fusiform to ellipsoidal, 2.2–3.8 × 1.7–2.5	Ant	Chen et al. (2025c)
*G. pupicola*	White, reverse yellowish, reaching 47–49 mm diam	Numerous denticles, 10.0–23.4 × 0.7–1.0	Fusiform to ellipsoidal, 2.5–4.2 × 1.0–2.1	Lepidoptera	Chen et al. (2025c)
*G. sinensis*	White, reverse yellowish, with radial patterns; reaching 37–38 mm diam.	Numerous denticles, 7.0–12.0 × 1.0–1.5	Ellipsoidal to fusiform, 2.4–3.9 × 1.5–2.8	Spider	Chen et al. (2025b)
*G. vallensis*	White, reverse yellowish, with radial patterns; reaching 25–36 mm diam.	Numerous denticles, 3.8–5.4 × 1.3–1.9	Ellipsoidal to fusiform, 2.3–3.0 × 1.7–1.9	Spider	Chen et al. (2025b)

Although *G. araneae*, *G. sinensis* and *G. vallensis* all parasitize spiders, *G. araneae* is characterized by markedly longer conidiogenous cells (11.7–31.7(–40.5) × 0.9–1.5 μm), compared with those of *G. sinensis* (7.0–12.0 × 1.0–1.5 μm) and *G. vallensis* (3.8–5.4 × 1.3–1.9 μm).

Taken together, although some morphological measurements overlap among species, the combination of morphological features, host association, and robust multilocus phylogenetic support justifies the recognition of *G. araneae* as a distinct species.

#### Gamszarella sotirae

Taxon classification

Animalia

HypocrealesClavicipitaceae

(Y.P. Tan, Bishop-Hurley & Marney), X.Y. Chang & M.J. Chen comb. nov.

6A44067A-70E3-5455-B209-F4B132163AEA

858511

##### Basionym.

*Lecanicillium sotirae* Y.P. Tan, Bishop-Hurley & Marney, Index Austral. Fungi 35: 7 (2024).

##### Host.

spider (*Araneae*).

**Distribution**. Australia.

##### Note.

This species was initially described as a new taxon by Tan and Shivas (2024b) due to distinctive differences in the ITS, nrLSU, *RPB2* and *TEF1* sequences. In both the multigene and single-locus phylogenetic analyses, this taxon formed a distinct and well-supported clade within *Gamszarella* (MLBS = 99, BPP = 1.00) (Fig. 2, Suppl. material 3). Consequently, we propose its reclassification into the genus *Gamszarella*.

The genus *Gamszarella* was established in 2023 based on molecular phylogenetic analyses and morphological traits, with *G. buffelskloofina* designated as the type species (Crous et al. 2023), and its circumscription has since been refined through molecular and morphological studies, leading to the transfer or segregation of several taxa into related genera such as *Corpulentispora* and *Neogamszarella* (Khonsanit et al. 2024; Chen et al. 2025b). With the inclusion of our current findings, *Gamszarella* currently comprises nine species. The fact that five species are araneopathogenic while the rest utilize insect hosts (Crous et al. 2023; Chen et al. 2025b, 2025c) highlights an intriguing evolutionary transition between host groups. Further studies are required to clarify host specificity and ecological associations within the genus *Gamszarella*.

#### Corniculantispora margaretspencerae

Taxon classification

Animalia

HypocrealesClavicipitaceae

(Y.P. Tan, Bishop-Hurley, R.G. Shivas & Marney), X.Y. Chang & M.J. Chen comb. nov.

1EF06499-F744-5EA4-8DDB-973F788E6B29

858513

Fig. 6

##### Basionym.

*Lecanicillium margaretspencerae* Y.P. Tan, Bishop-Hurley, R.G. Shivas & Marney, Index Austral. Fungi 15: 1 (2023).

**Figure 6.**
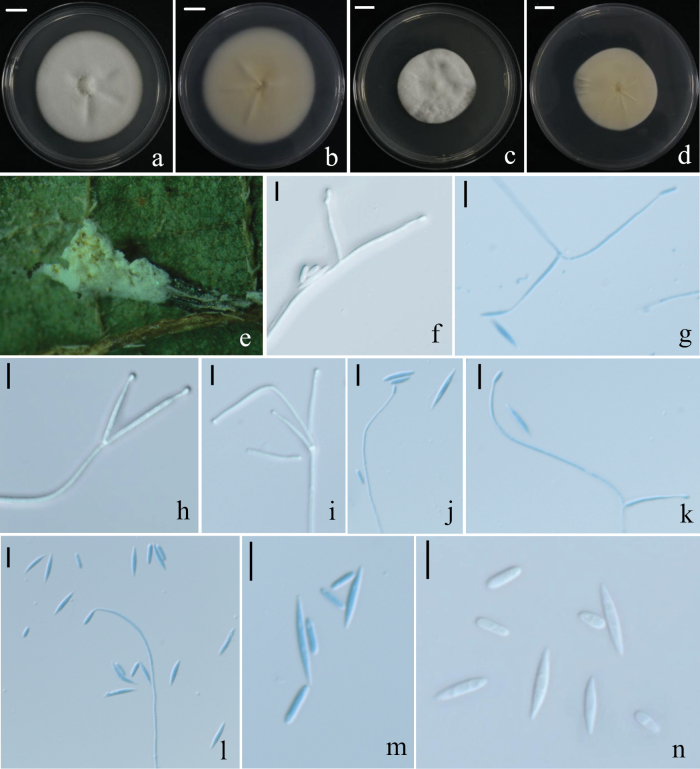
Holotype specimen and microscopic characteristics of *C. margaretspencerae*. **a, b**. Colony obverse and reverse on PDA; **c, d**. Colony obverse and reverse on SDAY/4; **e**. Infected host spider; **f–l**. Conidiogenous structures and conidia; **m, n**. Mature conidia. Scale bars: 10 mm (**a–d**); 5 um (**f–n**).

##### Typification.

China • Guizhou Province, Tongren City, Fanjingshan National Nature Reserve (27°50'N, 108°47'E), on dead spiders (*Araneae*), July 2024; Xiaoyun Chang and Mingjun Chen, holotype FJS20240724-86, ex-type RCEF7796.

##### Description.

On the host spider, the body is completely overlaid by a dense, tufted mycelium, ranging from pale yellow to yellowish cream. Hyphae hyaline, smooth-walled, branched, 0.7–1.5 μm wide. Phialides cylindrical, slightly tapering toward the apex, arising from hyphae or differentiated conidiophores, solitary or in whorls of 2–3, measuring (7.8–)12–40(–46) × 0.6–1.5 μm. Conidia are usually produced singly at the tips of phialides and occur in two forms: macroconidia fusiform, typically with sharp ends, straight or slightly curved, measuring 6.6–12.2 × 0.9–1.7 μm; microconidia ellipsoidal, straight or slightly curved, measuring 3.1–5.3 × 1–2 μm.

##### Culture characteristics.

On PDA, colonies are white on the surface, with the reverse pale beige at the center and lighter toward the margins. Mycelium is dense, and the colony reaches approximately 64 mm in diameter after 14 days at 25 °C. On 1/4 SDAY, colony growth is slower; the surface is white, and the reverse is beige, with distinct radial cracks.

##### Host.

spider (*Araneae*), insect.

##### Additional material examined.

China • Guizhou Province, Tongren City, Fanjingshan National Nature Reserve (27°50'N, 108°47'E), on a dead spider, July 2024; Xiaoyun Chang and Mingjun Chen, RCEF7877 (living culture). Australia, Queensland, Tully, on an unidentified dead insect, April 2021; T.S. Marney、M.D.E. Shivas and R.G. Shivas, BRIP 72656a.

##### Note.

In our multigene phylogenetic tree, strains RCEF7796 and RCEF7877 grouped with *Lecanicillium margaretspencerae* BRIP 72656a, with strong support (MLBS = 84, BPP = 1.0) (Fig. 1). However, *L. margaretspencerae* was placed with maximal support (MLBS = 100, BPP = 1.0) within the *Corniculantispora* clade, rather than the core *Lecanicillium* group, indicating a distant phylogenetic relationship from the latter. To accurately reflect its phylogenetic position, we propose the new combination *Corniculantispora margaretspencerae*. Originally described from Australia, this species is reported here for the first time from China, representing a new record for the country. While initially introduced by Tan and Shivas (2023c) from a single strain in Australia based on ITS, nrLSU, *TEF1* and *RPB2* sequences, it lacked detailed morphological description.

Our discovery of this species in Guizhou Province, China, confirmed its identity as conspecific with BRIP 72656a, despite minor sequence differences, which are considered intraspecific variation. This study provides the first comprehensive morphological description of the species and establishes strain RCEF7796 as the epitype, thereby stabilizing its taxonomic concept.

#### Corniculantispora saksenae

Taxon classification

Animalia

HypocrealesClavicipitaceae

(Y.P. Tan, Bishop-Hurley & Marney), X.Y. Chang & M.J. Chen comb. nov.

59D594FE-C415-59A2-B9F9-797830B61D6C

858510

##### Basionym.

*Lecanicillium saksenae* (Kushwaha) Kurihara & Sukarno, Mycoscience 50 (5): 377 (2009).

##### Synonyms.

*Verticillium saksenae* Kushwaha, Curr. Sci. 49: 948 (1980).

#### Gibellula alba

Taxon classification

Animalia

HypocrealesClavicipitaceae

X.Y. Chang & M.J. Chen sp. nov.

B47FD890-55D6-5047-8D9D-0B977A05340F

859737

Fig. 7

##### Etymology.

alba (Latin), white, refers to the white hyphae that wrap around the host.

**Figure 7.**
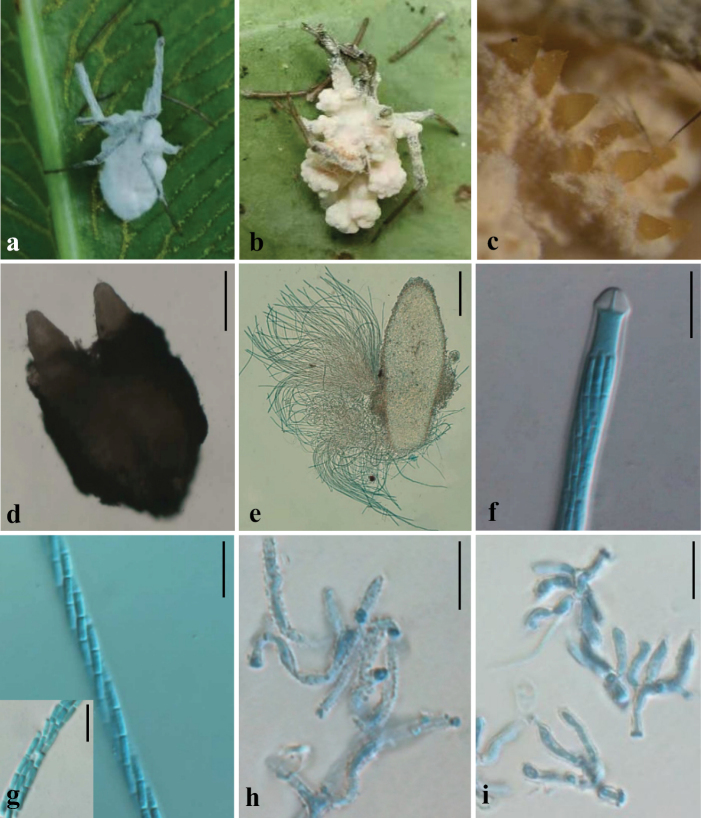
Morphological characteristics of *G. alba*. **a–c**. Fungus on a spider; **d, e**. Perithecium; **f**. Ascus; **g**. Ascospores; **h**. Rough-walled conidiophores; **i**. Granulomanus synanamorph. Scale bars: 200 μm (**d, e**); 10 μm (**f–i**).

##### Typification.

China • Anhui Province, Chizhou City, Shitai County, Guniujiang Scenic Area (30°04'N, 117°29'E), on a dead spider (*Araneae*), July 2021; Ting Wang and Mingjun Chen, holotype GNJ20210711-02.

##### Description.

Except for the metatarsi and tarsi, the host spider is entirely covered by a dense mat of mycelium. In the early stage of infection, the mycelium is pure white (Fig. 7a); during the mid to late stages, the mycelial mat turns flesh pink (Fig. 7b), and perithecia become aggregated on the dorsal side of the host’s abdomen. Perithecia are ivory to maize yellow, translucent, and ellipsoidal to clavate in shape, measuring 673–802 × 236.5–296.5 μm. Asci are cylindrical, measuring 505–700 μm in length and 4.5–6.5 μm in width. Ascospores are filiform, multiseptate, and arranged either in parallel or in a helical manner, measuring (666–)670–727(–730) × 2–3 μm. The ascus cap is hemispherical, 5–6 × 3.5–4 μm. Ascospores fragment into secondary ascospores, which are short-cylindrical and measure 4–6 × 1.5–2 μm. Conidiophores bear verrucose (warty) surfaces, and no clearly defined conidiogenous structures were observed at the apex. The asexual morph is recognized as *Hirsutella*-like.

##### Host.

spider (*Araneae*).

##### Additional material examined.

China • Anhui Province, Chizhou City, Shitai County, Guniujiang Scenic Area (30°04'N, 117°29'E), on dead spiders, July 2021; Ting Wang and Mingjun Chen, GNJ20210711-03.

##### Note.

*G. alba*, collected in Anhui, China, is described here as a new species of the cosmopolitan spider-pathogenic genus *Gibellula*. Consistent genealogical separation of *G. alba* from its closest relatives was recovered based on the rDNA (ITS+nrLSU) and *TEF1* loci (Suppl. material 3: fig. S4). Polygenetic phylogenetic analyses indicate that *G. alba* forms a distinct and well-supported clade (MLBS = 100, BPP = 1.0), showing close relatedness to *G. pigmentosinum* (Fig. 2). A BLASTN search of the GenBank database revealed notable sequence differences between *G. alba* and *G. pigmentosinum*, with nrLSU and *TEF1* sequence identities at 96.33% and 96.18%, respectively, compared to *G. pigmentosinum* strain BCC41870. These genetic markers offer a reliable method for distinguishing *G. alba* from closely related species. Due to specialized growth requirements, most *Gibellula* species are difficult to culture in vitro, and the absence of diagnostic morphological or molecular characters for some taxa has caused considerable taxonomic confusion (Samson and Evans 1973; Chen et al. 2022a). In *G. alba*, only the sexual morph was clearly observed, which is notable given that most congeners are known solely from their asexual morphs, while sexual or granulomanus synasexual forms have been reported only rarely (Evans et al. 2025). As the number of described species continues to grow, the genus exhibits remarkable morphological diversity, yet the mechanisms and ecological triggers governing its reproductive mode remain largely unknown, and the structure and biological role of granulomanus synasexual morphs are still speculative. Fewer than eighteen *Gibellula* species have been reported from China, but members of this genus are increasingly valued for their potential in the discovery of bioactive secondary metabolites, chemotaxonomic markers, and insights into host-pathogen interactions, highlighting the need for continued sampling and integrated morphological-molecular studies to refine the taxonomy of this ecologically specialized lineage.

#### Gibellula guizhouensis

Taxon classification

Animalia

HypocrealesClavicipitaceae

X.Y. Chang & M.J. Chen sp. nov.

665A392B-06E5-58A2-9DF1-5803338C77F7

861831

Fig. 8

##### Etymology.

name refers to “Guizhou Province” where the holotype was collected.

**Figure 8.**
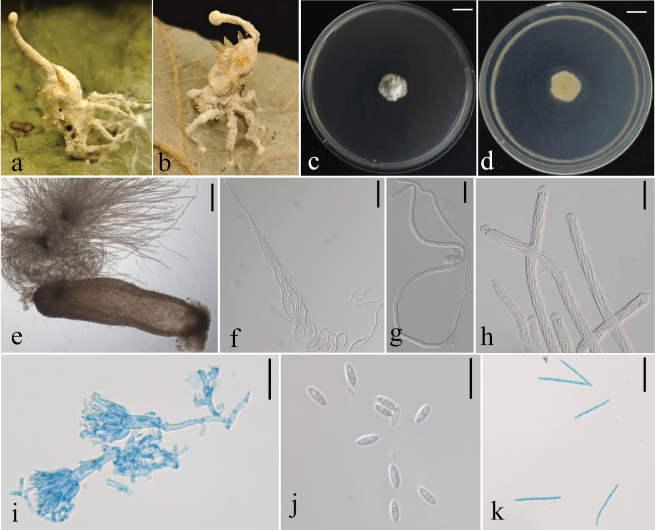
Morphological characteristics of G. sp. **a, b**. Fungus on a spider; **c, d**. Colony obverse and reverse on PDA; **e**. Perithecium; **f, g**. Ascus; **h**. Ascus tip; **i**. Conidiophores; **j**. Conidia; **k**. Ascospores. Scale bars:10 mm (**c, d**); 200 μm (**e**); 100 μm (**f**); 50 μm (**g**); 20 μm (**h, i**); 10 μm (**j, k**).

##### Typification.

China • Guizhou Province, Tongren City, Fanjingshan Nature Reserve (27°50'N, 108°47'E), on a dead spider (*Araneae*), July 2025; Xiaoyun Chang and Mingjun Chen, holotype FJS20250705-58, ex-type RCEF8165.

##### Description.

Mycelial mat is creamy yellow and velvety, completely covering the spider host, firmly attaching the host to underside of living leaf by the mycelia covering its legs. Synnema solitary, arising from the posterior region of the host abdomen, cylindrical, consisting of a compact bundle of parallel hyphae, with a blunt tip. Conidiophores rising from mycelial mat and synnema, smooth, septate, cylindrical, predominantly biverticillate, 50–90(-104) × 4–5.5 μm, vesicles rarely developed. Several metulae are borne on the apex of conidiophore. Metulae cylindrical, 8.5–11.5 × 4.5–6 μm, with a number of phialides in whorls. Phialides broadly cylindrical, with the apex tapering abruptly to a short neck 10–13 × 2.5–3.5 μm. Conidia fusiform, 5–7 × 1.7–3 μm, in chains, borne on each phialide. Perithecia occurring on the mycelial mat covering the host legs, superficial, mostly arranged in groups, cylindrical, reddish yellow, one-third immersed in the network of mycelia, 900–1300 × 310–350 μm. Asci (242-)350–740(-813) × (3-)5–8.5(-10) μm, ascus tip 4–6 × 7–9 μm. Ascospores often breaking into part-spores. Part-spores bacilliform, 21–29(-31) × 1.4–2.1 μm. Granulomanus-like asexual morph absent.

##### Host.

spider (*Araneae*).

##### Additional material examined.

China • Guizhou Province, Tongren City, Fanjingshan Nature Reserve (27°50'N, 108°47'E), on a dead spider, July 2025; Xiaoyun Chang and Mingjun Chen, RCEF8164, RCEF8166, RCEF8168, RCEF8170.

##### Note.

*G. guizhouensis*, discovered in guizhou, China, is formally described here as a novel species within the cosmopolitan spider-pathogenic genus *Gibellula*. Consistent genealogical separation of *G. guizhouensis* from its closest relatives was recovered based on the rDNA (ITS+nrLSU), *TEF1*, *RPB1* and *RPB2* loci (Suppl. material 3: fig. S4). Polygenetic phylogenetic analyses indicate that *G. guizhouensis* forms a distinct and well-supported clade (MLBS = 100, BPP = 1.0), showing close relatedness to *G. clavulifera* var. alba (Fig. 2). A BLASTN search of the GenBank database revealed notable sequence differences between *G. guizhouensis* and *G. clavulifera* var. alba, with ITS, nrLSU, *TEF1*, *RPB1* and *RPB2* sequence identities at 96.43%, 97.76%, 97.41%, 96.18%, and 96.41%, respectively, compared to *G. clavulifera* var. alba strain ARSEF1915. Compared with the typical characteristics, *G. guizhouensis* was distinguished from *G. clavulifera* var. alba by its shorter metulae (8.5–11.5 × 4.5–6 μm vs 9–15 × 3–4 μm) and wider phialides (10–13 × 2.5–3.5 μm vs 10–12.4 × 1.5–2.5 μm) (Humber and Rombach 1987). The integration of distinctive morphological traits and robust multi-locus phylogenetic evidence provides support for the establishment of *G. guizhouensis* as a new species.

#### Hevansia pseudonelumboides

Taxon classification

Animalia

HypocrealesClavicipitaceae

X.Y. Chang T. Wang & M.J. Chen sp. nov.

D25DF857-C298-5F8D-9701-9307B7C556C3

860241

Fig. 9

##### Etymology.

refers to the morphological similarity of this species to *Hevansia nelumboides*, with the prefix “pseudo-” (false) indicating its close yet distinct taxonomic relationship.

**Figure 9.**
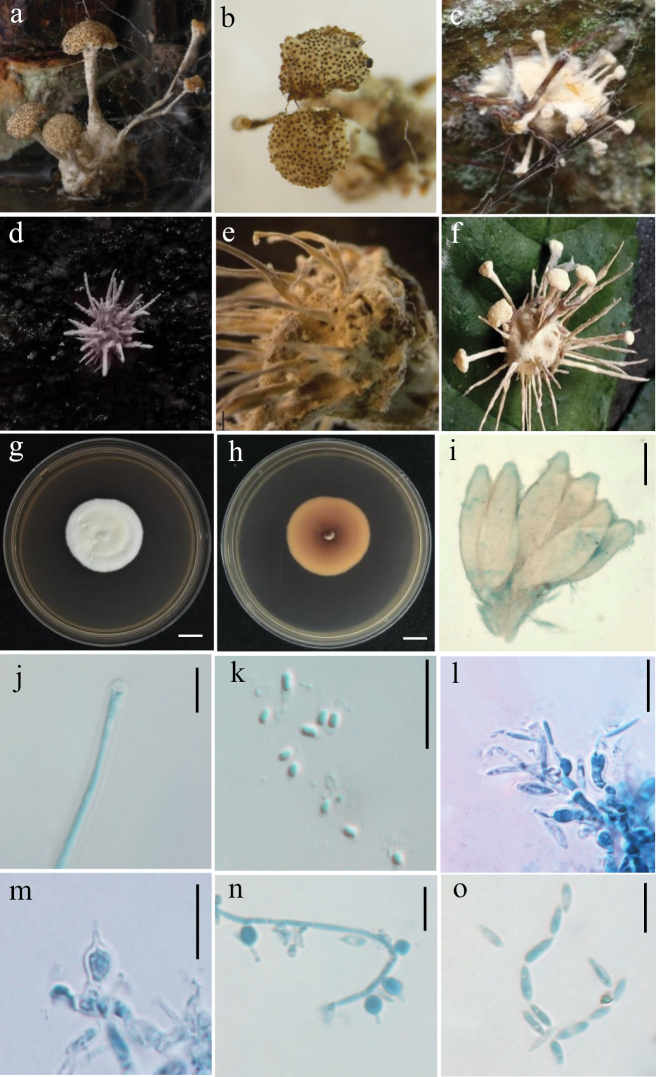
Morphological characteristics of *H. pseudonelumboides*. **a–f**. The infected host spider exhibits both sexual and asexual morphs simultaneously; **g, h**. Colony obverse and reverse on PDA; **i**. Perithecium; **j**. Ascus; **k**. ascospores; **i–n**. Conidiogenous structure; **o**. Conidia. Scale bars:10 mm (**g, h**); 100 μm (**f**); 10 μm (**j–o**).

##### Typification.

China • Jiangxi Province, Jiujiang City, Lushan Mountain (29°32'N, 115°58'E), on a dead spider (*Araneae*), August 2023; Xiaoyun Chang and Mingjun Chen, holotype LS20230802-71, ex-type RCEF7275.

##### Description.

Sexual morph: The host spider is entirely covered by mycelium, with stromata emerging solitarily and unbranched from the dorsal side of the host. Stipes cylindrical, 2.0–3.6 mm long and 0.4–0.9 mm thick, initially pale orange, later becoming pale yellow or white. The fertile parts are rosette-shaped, 1.7–2.3 mm in diameter, light orange in early stages and turning light brown in maturity. Perithecia are immersed, subellipsoidal, dark brown, measuring 553–564 × 109.8–175.6 μm. Asci are cylindrical, 133–165 × 5.1–5.8 μm, with a hemispherical apical cap measuring 4.6–5.1 × 2.5–2.9 μm. Ascospores were not clearly observed, but scattered secondary spores were present, capsule-like, measuring 1.6–2.1 × 0.8–1.1 μm. Asexual morph: Synnemata are densely arranged on the surface of the spider body. In fresh specimens, they are grayish white and slender conical. When dried, they become pale yellow and resemble slightly curled bamboo leaves, measuring 3.1–6.7 mm in length and 108–185 μm in width at the middle. Phialides are globose to ellipsoidal, sparsely distributed on the hyphae, measuring 4.5–6.2 × 4.4–5.8 μm, abruptly tapering into a narrow neck at the apex. Conidia are fusiform to cylindrical, forming in chains, and measure 6.5–8.7 × 2.1–2.8 μm.

##### Culture characteristics.

On PDA, colonies are white on the surface and pale yellowish brown to light brownish orange on the reverse, with a purplish center. The mycelium is dense, and growth is slow, reaching a diameter of up to 36 mm after 14 days at 25 °C.

##### Additional material examined.

China • Anhui Province, Chizhou City, Shitai County, Guniujiang Scenic Area (30°04'N, 117°29'E), on dead spiders, July 2021; Ting Wang and Mingjun Chen, GNJ20210710-05, GNJ20210710-08 and GNJ20210711-04. China • Jiangxi Province, Jiujiang City, Lushan Mountain (29°32'N, 115°58'E), on a dead spider, August 2023; Xiaoyun Chang and Mingjun Chen, RCEF7270. China • Guizhou Province, Tongren City, Fanjingshan National Nature Reserve (27°50'N, 108°47'E), on a dead spider, July 2024; Xiaoyun Chang and Mingjun Chen, RCEF7801.

##### Note.

Phylogenetically, multilocus analyses support *H. pseudonelumboides* as a distinct and well-supported monophyletic lineage (Fig. 2). Moreover, single-locus phylogenies based on rDNA (ITS+nrLSU), *TEF1*, *RPB1* and *RPB2* exhibit genealogical concordance, each recovering *H. pseudonelumboides* as monophyletic (Suppl. material 3: fig. S5), consistent with the criteria of GCPSR. Sexually, all four *Hevansia* species possess stipitate stromata, with two distinct types of fertile structures: *H. minuta* features oval fertile parts, while the remaining three species have disc-shaped structures. Perithecia are consistently immersed across all species. The asci are cylindrical, but those of *H. pseudonelumboides* are significantly shorter compared to the other three species, providing a useful distinguishing characteristic (Table 3). Asexually, synnemata are absent in *H. minuta*, whereas the other species develop numerous synnemata. Phialides are generally subglobose with distinct necks, except *H. longispora*. Conidia are typically cylindrical or fusiform, with size variations among species (Table 4). Additionally, both *H. pseudonelumboides* and *H. novoguineensis* produce a purple pigment that diffuses into PDA medium, whereas *H. minuta* does not produce any pigment. Importantly, all species in this genus are spider pathogens, indicating that *Hevansia* comprises a group of obligate, host-specific entomopathogenic fungi specialized on targeting spiders.

**Table 3.** Morphological comparisons of sexual morphs in *Hevansia*.

Species	Host	Stromata	Fertile part	Perithecia	Asci	Ascospores	References
*Hevansia minuta*	Spider (*Theridiidae*, *Meotipa* sp.)	Stipitate, solitary, white to cream, 10 mm long, 1 mm broad	Oval, ca. 2–2.5 mm long, ca. 1.5 mm broad	Immersed, narrowly ovoid, 400–500 × 100–170 µm	Cylindrical, 325–450 × 3–5 µm	Filiform, whole ascospores, 320–450 × 0.5–1.5 µm	Mongkolsamrit et al. (2022)
*H. nelumboides*	Spider	Stipitate, white, 4 mm long, 0.4 mm broad	Disc-shaped, 2 × 0.8 mm	Immersed, fusoid-ellipsoidal, 535–545 × 180–190 µm	400–450 × 5–6 µm	Part-spores, ca. 5 × 1 µm	Kobayasi and Shimizu (1977)
*H. novoguineensis*	Spider (*Theridiidae*)	Stipitate, solitary, or multiple, cylindrical, white to pale yellow, 3–5 mm long, 0.5–1 mm broad	Disc-shaped, upper surface slightly con vex, 1–3 × 1–2 mm	Immersed, narrowly ovoid, 500–750 × 200–300 µm	Cylindrical, 350–450 × 5–7 µm	Filiform, whole ascospores, 400–460 × 1–1.5 µm	Mongkolsamrit et al. (2022)
*H. pseudonelumboides*	Spider	Stipitate, solitary, Stipes cylindrical, initially pale orange, later becoming pale yellow or white, 2.0–3.6 mm long and 0.4–0.9 mm thick	Disc-shaped, 1.7–2.3 mm in diameter	Immersed, subellipsoidal, dark brown, 553–564 × 109.8–175.6 μm	Cylindrical, 133–165 × 5.1–5.8 μm	Ascospores not observed, secondary spores capsule-like, 1.6–2.1 × 0.8–1.1 μm	This study

**Table 4.** Morphological comparisons of asexual morphs in *Hevansia.*

Species	Host	Synnemata	Phialides	Conidia	References
*Hevansia arachnophila*	Spider	Simple, solitary (rarely two or three together), cylindrical, cream, up to 6 mm long, 45–100 µm broad	Globose, 3–4.5 µm broad, with distinct necks, 1–2 × 0.5 µm	Cymbiform, 3.5–6 × 1–1.5 µm	Hywel-Jones (1996)
*H. longispora*	Spider	Multiple, clavate, brown, 250–700 µm long	Ellipsoid to cylindrical, 7–15 × 2–4 µm	Cylindrical to fusiform, 8.8–14.8 × 2–3 µm	Huang et al. (2000)
*H. mainiae*	Spider	Numerous, simple, filiform, 3–5 mm long and 50–150 µm wide, composed of textura porrecta.	Phialidic globose to lageniform or obovoid, 4–11 × 4–6 µm, with a short cylindrical neck 2.5–5 × 1 µm, hyaline, thin-walled	Cylindrical to fusoid, rounded at apex, narrowed near the base, with a minute refractive hilum, 7–12 × 1.5–2 µm	Crous et al. (2023)
*H. minuta*	Spider (*Theridiidae*, *Meotipa* sp.)	Non-synnemata	Globose to ovoid, 5–7 × 5–6 µm with distinct necks, 1–2 × 0.5 µm	Fusiform, 2–7 × 2–3 µm	Mongkolsamrit et al. (2022)
*H. nelumboides*	Spider (*Theridiidae*)	NA	Elongate	Ovoid, 5 × 3 µm	Kobayasi and Shimizu (1977)
*H. novoguineensis*	Spider	Multiple, cylindrical, occasionally acuminate apex, white, up to 8 mm long, 50–200 µm broad	Globose to subglobose, 4–6 × 4–6 µm, with distinct necks, 0.5–1.5 × 0.5–1 µm	Fusoid or fusiform elliptical, 2–10 × 1–2.5 µm	Mongkolsamrit et al. (2022)
*H. novoguineensis*	Spider	Multiple, slender, acuminate apex, white to pale yellow, 3.5 mm long, 50–150 µm broad	Globose to ovoid, 5–6.5 × 4–6 µm broad, with distinct necks, 2–3 × 0.8–1.5 µm	Cylindrical, curved or slightly fusiform, 10.5–17.5 × 1.5–3 µm	Samson and Brady (1982)
*H. ovalongata*	Spider	Multiple, simple, or branch, white to greyish orange, 2.2–9 mm long, 112–520 µm broad	Globose to subglobose, cylindrical, or ellipsoid, 6–8.7 × 4–6.4 µm, with distinct necks, 1.4–3.2 × 0.8–1.8 µm	Ellipsoid, obovate to oblong, 6–10.3 × 2.4–4.4 µm	Hsieh et al. (1997)
*H. pseudonelumboides*	Spider	Numerous, simple, filiform, 3.1–6.7 mm long, 108–185 μm broad	Globose to ellipsoidal, 4.5–6.2 × 4.4–5.8 μm, abruptly tapering into a narrow neck at the apex	Fusiform to cylindrical, 6.5–8.7 × 2.1–2.8 μm.	This study

#### Husseyia sinensis

Taxon classification

Animalia

HypocrealesClavicipitaceae

X.Y. Chang & M.J. Chen sp. nov.

01CD11FF-6DF7-509A-B9E3-C134BC157DDB

861832

Fig. 10

##### Etymology.

Referring to the country where the fungus was first discovered.

**Figure 10.**
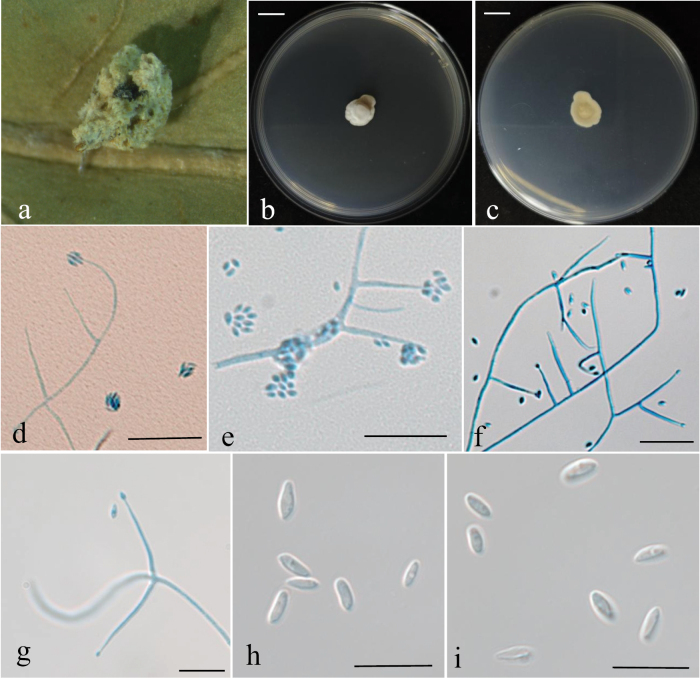
Holotype specimen and microscopic characteristics of *H. sinensis*. **a**. Infected host spider; **b, c**. Colony obverse and reverse on PDA; **d–g**. Conidiogenous structures; **h, i**. Mature conidia. Scale bars: 10 mm (**b, c**); 20 μm (**d–f**); 10 μm (**g–i**).

##### Typification.

China • Guizhou Province, Tongren City, Fanjingshan Nature Reserve (27°50'N, 108°45'E), on a dead spider (*Araneae*), July 2025; Xiaoyun Chang and Mingjun Chen, holotype FJS20250706-97, ex-type RCEF8284.

##### Description.

The host spider is completely enveloped by a tufted, pale yellow mycelium. Hyphae septate, hyaline, smooth-walled, 1.0–2.5 μm wide. Conidiophores hyaline, smooth-walled, emerging from aerial hyphae or chondroid mycelium, with single phialide or whorls of 2 phialides or verticillium-like from hyphae directly. Phialides slightly swollen at the base, cylindrical, 15–30(-67) × 0.7–1.7 μm, gradually tapering into a narrow neck. Conidia hyaline, smooth-walled, predominantly elliptical, ovoid or fusiform, 3.1–6.1(-7.7) × 1.4–2.5(-4.0) μm, aggregated in slimy heads at the apex of phialides.

##### Culture characteristics.

On PDA, colonies are white to pale yellow on the surface and pale yellow on the reverse, with lighter-colored margins. Mycelium is dense and cottony, with slow growth, reaching 13–16 mm in diameter after 14 days at 25 °C.

##### Host.

spider (*Araneae*).

##### Additional material examined.

China • Anhui Province, Luan City, Wanfoshan Nature Reserve (31°03'N, 116°33'E), on a dead spider, June 2025; Xiaoyun Chang and Mingjun Chen, RCEF8222.

##### Note.

The genus *Husseyia* was recently established by Tan and Shivas (2023c) and currently comprises only two species, *H. annamariae* and *H. queenslandica*, both of which were described solely based on molecular phylogenetic data, without accompanying morphological information. Consequently, detailed morphological comparisons between *H. sinensis* and the previously described species of the genus have not been possible until now. In the present study, single-locus phylogenetic trees based on rDNA (ITS+nrLSU), *TEF1*, *RPB1*, and *RPB2* consistently resolved *H. sinensis* as a distinct lineage from its closest relatives, in accordance with the GCPSR criterion (Suppl. material 3: fig. S5). Moreover, *H. sinensis* formed an independent and strongly supported clade (MLBS = 98, BPP = 1.0) in the multigene molecular phylogenetic analyses (Fig. 3) and is characterized by a unique combination of morphological features, including conidiophores bearing single phialides or whorls of two phialides, occasionally forming *Verticillium*-like structures, and ellipsoidal to fusiform conidia aggregated in slimy heads at the apex of phialides. Collectively, the present study provides the first comprehensive morphological characterization within the genus *Husseyia*, thereby contributing to a better understanding of its taxonomy.

#### Liangia guizhouensis

Taxon classification

Animalia

HypocrealesClavicipitaceae

X.Y. Chang & M.J. Chen sp. nov.

CE650562-906A-500C-B851-AA27DFDCEC25

858518

Fig. 11

##### Etymology.

name refers to “Guizhou Province” where the holotype was collected.

**Figure 11.**
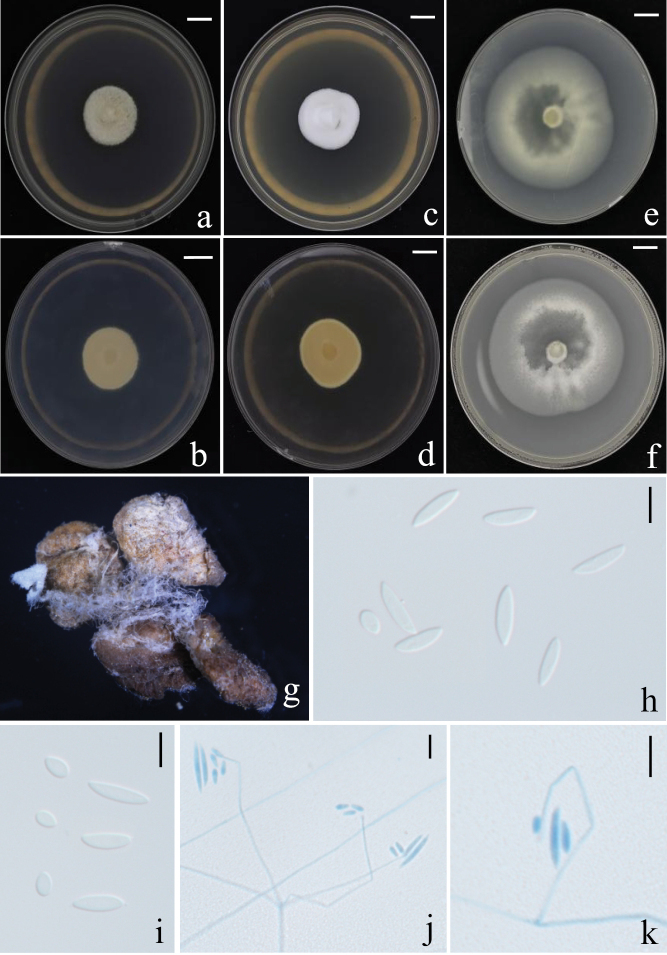
Holotype specimen and microscopic characteristics of *L. guizhouensis*. **a**. Infected host spider; **b, c**. Colony obverse and reverse on PDA; **d, e**. Colony obverse and reverse on SDAY/4; **f–i**. Conidiogenous structures and conidia; **j**. Mature conidia. Scale bars: 10 mm (**a–f**); 10 μm (**h–k**).

##### Typification.

China • Guizhou Province, Tongren City, Fanjingshan National Nature Reserve (27°50'N, 108°47'E), on the spider egg sac that could not be accurately identified, July 2024; Xiaoyun Chang and Mingjun Chen, holotype FJS20240724-85, ex-type RCEF7795.

##### Description.

Hyphae hyaline, septate, branched, with smooth walls, 0.7–3.0 µm wide. Phialides cylindrical, occurring directly from the prostrate hyphae, gradually attenuated toward the apex, either solitary or in clusters of 2–4, 14.5–36.7 × 0.6–1.3 µm. The conidia exist in two types: macroconidia and microconidia. Both types are aseptate, transparent, with smooth walls, single-celled, and upright, either solitary or paired at the apex of the conidiophore. Both types of conidia can be produced from the same phialides, often appearing together. macroconidia fusiform or semi-ellipsoidal, 5.7–11.7 × 1.2–2.5 µm (x̄ = 8.0 ± 1.3 × 1.9 ± 0.3 µm); microconidia oval or ellipsoidal, 2.2–4.1(–5.0) × 1.1–2.5(–3.0) µm (x̄ = 3.0 ± 0.6 × 1.6 ± 0.4 µm).

##### Culture characteristics.

On PDA, colonies are white on the surface and pale yellow on the reverse, with felt-like mycelium and slow growth, reaching approximately 25 mm in diameter after 14 days at 25 °C. On MEA, colonies are white on the surface and pale brown on the reverse, with lighter coloration around the margins. On OA, colonies are white with thin and sparse mycelium; the center lacks mycelial growth, but the colony spreads relatively quickly.

##### Host.

unidentified spider egg sac.

##### Note.

Phylogenetic analyses have confirmed that *L. guizhouensis* forms a distinct and well-supported clade (MLBS = 100, BPP = 1.0), closely related to *L. sinensis*. These are currently the only two known species within the genus. In addition, single-locus phylogenies based on rDNA (ITS+nrLSU), *TEF1*, *RPB1* and *RPB2* showed genealogical concordance, each supporting the monophyly of *L. guizhouensis*, consistent with the criteria of GCPSR (Suppl. material 3: fig. S7). A BLASTN search against the GenBank database revealed significant sequence divergence between the type strains of *L. guizhouensis* and *L. sinensis* YFCC 3103, with identities of 96.64% for ITS, 99.28% for nrLSU, 95.47% for *TEF1*, 91.55% for *RPB1*, and 86.71% for *RPB2*, providing molecular evidence for species delimitation.

Morphologically, *L. guizhouensis* and *L. sinensis* share similar characteristics, yet they exhibit slight differences in the structure of phialides and the morphology of their two types of conidia. In *L. guizhouensis*, phialides are solitary or arranged in verticils of 2–4, measuring 14.5–36.7 × 0.6–1.3 μm. The macroconidia are fusiform to subellipsoidal, 5.7–11.7 × 1.2–2.5 μm, while the microconidia are ovoid to ellipsoidal, 2.2–5.0 × 1.1–3.0 μm. In contrast, *L. sinensis* has solitary phialides, 16.7–59.0 × 0.7–1.6 μm in size. Its macroconidia are oblong to fusiform, 4.5–9.3 × 1.2–1.9 μm, and its microconidia are ellipsoidal to ellipsoid, measuring 1.8–3.3 × 1.1–1.8 μm (Wang et al. 2020). These clear morphological distinctions (Table 5), together with multi-locus phylogenetic support, allow *L. guizhouensis* to be confidently recognized as a new species.

**Table 5.** Morphological comparisons of asexual morphs in *Liangia*.

Characteristic	*L. sinensis*	*L. guizhouensis*
Colony growth (PDA)	28–34 mm in 14 days at 25 °C, slow-growing	25 mm in 14 days at 25 °C, slow-growing
Colony color	White, reverse pale brown	White, reverse pale yellow
Hyphae	Hyaline, septate, branched, smooth-walled, and 0.7–2.4 µm wide.	hyaline, septate, branched, with smooth walls, 0.7–3.0 µm wide
Phialides	Lanceolate, occurring directly from the prostrate hyphae, solitary, gradually attenuated toward the apex, 16.7–59.0 µm long, 0.7–1.6 µm wide at the base and 0.3–0.7 µm wide at the apex.	cylindrical, occurring directly from the prostrate hyphae, gradually attenuated toward the apex, either solitary or in clusters of 2–4, 14.5–36.7 × 0.6–1.3 µm
Macroconidia	Oblong-oval to fusiform, 4.5–9.3 × 1.2–1.9 µm	Fusiform or semi-ellipsoidal, 5.7–11.7 × 1.2–2.5 µm (x̄ = 8.0 ± 1.3 × 1.9 ± 0.3 µm)
Microconidia	Oval to ellipsoidal, 1.8–3.3 × 1.1–1.8 µm	Oval or ellipsoidal, 2.2–4.1(–5.0) × 1.1–2.5(–3.0) µm (x̄ = 3.0 ± 0.6 × 1.6 ± 0.4 µm)
References	Wang et al. (2020)	This study

#### Nuciformispora araneae

Taxon classification

Animalia

HypocrealesClavicipitaceae

(Sukarno & Kurihara) X.Y. Chang & M.J. Chen nom. nov.

1CA7DCD1-1542-5B2D-A34D-CC104B05E84B

858506

##### Basionym.

*Lecanicillium araneicola* Sukarno & Kurihara, Mycoscience 50 (5): 369–379 (2009).

#### Nuciformispora rasoulzarei

Taxon classification

Animalia

HypocrealesClavicipitaceae

(A. Armand & Khodap.) X.Y. Chang & M.J. Chen comb. nov.

B27A0DD1-D6C6-5318-B8A2-CE4CACB17773

858507

##### Basionym.

*Lecanicillium rasoulzarei* A. Armand & Khodap., Arch. Microbiol. 206 (no. 202): 11 (2024).

#### Nuciformispora spenceae

Taxon classification

Animalia

HypocrealesClavicipitaceae

(Y.P. Tan, Bishop-Hurley & Marney) X.Y. Chang & M.J. Chen comb. nov.

ADC8A1F5-AD31-55A1-ADD9-90D31F4FB18D

858508

##### Basionym.

*Lecanicillium spenceae* Y.P. Tan, Bishop-Hurley & Marney, Index Austral. Fungi 22: 3 (2023).

##### Note.

Our multigene phylogenetic analyses indicate that three members of the former *Lecanicillium aranearum* species complex—*L. araneicola*, *L. spenceae*, and *L. rasoulzarei*—are phylogenetically nested within *Nuciformispora*, and therefore should be transferred to this genus. This result addresses a long-standing taxonomic uncertainty first recognized by Zare and Gams (2001), who noted that this morphologically and ecologically cohesive group required further investigation.

The genus *Nuciformispora* was only recently established in 2025, with *N. sinense* designated as the type species; its members are predominantly associated with spiders and insects (Chen et al. 2025c). *N. araneae*, *N. spenceae*, and *N. rasoulzarei* conform well to this host range. Morphologically, these three species also agree with the diagnostic characteristics of *Nuciformispora*, including white colonies in surface view, phialides occurring solitarily or in groups of two to four, and the presence or absence of octahedral crystals (Table 6). Therefore, these results support the placement of the three species within the genus *Nuciformispora*.

**Table 6.** Asexual morphology of *Nuciformispora* species associated with this study

Characteristic	*Nuciformispora aranearum*	*N. araneae*	*N. araneicola*	*N. rasoulzarei*
Colony growth (PDA)	40 mm in 10 days at 25 °C	Not mentioned	29–31 mm in 14 days at 25 °C	36–37 mm in 10 days at 25 °C
Colony color (surface)	White	White	White	White
Colony color (reverse)	Yellowish cream	Creamy-white	Yellowish	Light yellow
Hyphae	Not detailed	Aerial, prostrate, 1–2.5 μm wide	Prostrate hyphae smooth, septate, hyaline, 0.9–1.5 μm diam	Hyaline, septate, smooth-walled, 1–2 μm wide (x̄ = 1.2 μm)
Phialides	20–30 × 1.2–1.5 μm, tapering at apex	Solitary or in whorls of 2–4, 14–31.5 × 1–2 μm, slender, tapering	Solitary or phialides in whorls of two to three, 10.5–24.1 × 1.0–1.7 μm (x̄ = 16.7 × 1.3 μm), with a cylindrical basal portion, tapering into a distinct neck.	13–26 × 1–1.2 μm (x̄ = 20 × 1 μm); solitary, paired, or in whorls of 3–5; long, narrow, aculeate
Conidia type	Single type	Dimorphic (macro- and microconidia)	Dimorphic (macro- and microconidia)	Dimorphic (macro- and microconidia)
Macroconidia	slightly pointed at one or both ends.straight or curved, usually asymmetrically narrowed or subacute at the ends, measuring 5–8 × 0.7–1.5 μm	Acerose, 8.5–12(–14) × 1.5–2 μm, pointed ends, slightly curved or nearly straight	Date-pit shape, 8.0–12.9 × 1.4–2.1 μm (x̄ = 10.6 × 1.6 μm)	Falcate, 8–10(–12) × 1.2–1.5(–2) μm (x̄ = 9.5 × 1.3 μm); asymmetrically narrowed, pointed
Microconidia		Allantoid to ellipsoidal, 3–5 × 1–2 μm	Cylindrical, 2.8–4.6 × 0.9–1.4 μm (x̄ = 3.9 × 1.2 μm)	Short-cylindrical or ovoid, 3–5 × 1–1.2 μm (x̄ = 3.7 × 1 μm); pointed at one or both ends
Conidia arrangement	Not specified	Not specified	Not specified	In subglobose to globose heads
Octahedral crystal	Present	Not mentioned	Absent	Absent
References	Zare and Gams (2001)	Sukarno et al. (2009)	Chen et al. (2025c)	Armand et al. (2024)

This taxonomic revision further reassesses certain species previously assigned to *Lecanicillium* sensu lato and helps resolve some of the remaining issues associated with this broadly defined genus. *Lecanicillium* sensu lato has been shown to comprise multiple phylogenetically distinct lineages distributed among different genera within *Cordycipitaceae* (Zhou et al. 2022; Khonsanit et al. 2024). Nevertheless, several taxa currently placed in *Lecanicillium* remain unresolved. For example, *L. fusisporum* appears to represent an isolated lineage for which no appropriate generic placement is presently available.

#### Purpureocillium araneicola

Taxon classification

Animalia

HypocrealesClavicipitaceae

X.Y. Chang & M.J. Chen sp. nov.

26DF3C19-5A76-56C3-B5FA-D3B92506162F

858519

Fig. 12

##### Etymology.

referring to the ability to colonize spider.

**Figure 12.**
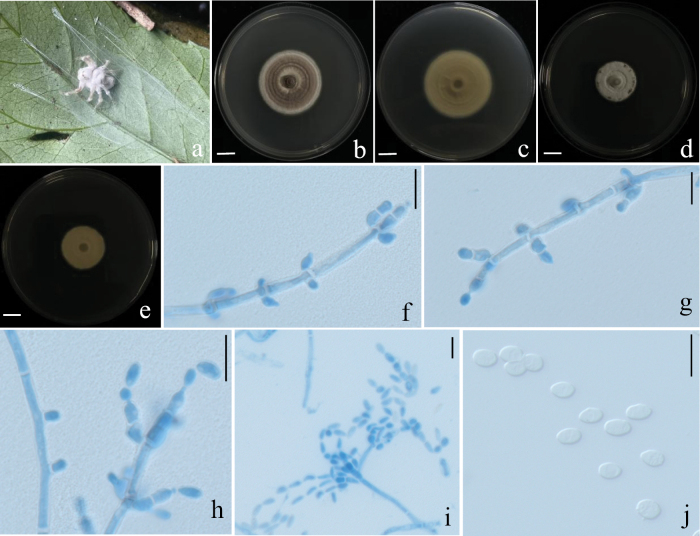
Holotype specimen and microscopic characteristics of *P. araneicola*. **a**. Infected host spider; **b, c**. Colony obverse and reverse on PDA; **d, e**. Colony obverse and reverse on SDAY/4; **f–i**. Conidiogenous structures and conidia; **j**. Mature conidia. Scale bars: 10 mm (**b–e**); 10 μm (**f–j**).

##### Typification.

China • Guizhou Province, Tongren City, Fanjingshan National Nature Reserve (27°50'N, 108°47'E), on a dead spider (*Araneae*), July 2024; Xiaoyun Chang and Mingjun Chen, holotype FJS20240723-50, ex-type RCEF7731.

##### Description.

A tomentose mycelium, grayish-white to pale grayish-purple, completely covers the body wall of the host spider, with abundant conidia produced on the surface at later stages. Hyphae hyaline, smooth-walled, branched, 1.5–3.0 μm wide. Conidiophores hyaline, erect, elongate, 50–186 × 2.2–4.5 μm, distinctly septate with internodes 10–40 μm long. Conidiophores produce short branches at septa; branches verticillate, hyaline, aseptate, short-cylindrical, 4.5–8.5 × 2.6–5.5 μm. Branch apices bear solitary or 2–5 verticillate phialides; phialides obovate to cylindrical at base, occasionally tapering apically, 4.5–8.0 × 2.6–4.5 μm. Conidia hyaline, smooth-walled, thin-walled, ellipsoidal to ovoid, formed in chains, 4.5–8.2 × 2.5–5.3 μm. The shape and size of phialides and conidia in culture conditions were similar to those on spiders.

##### Culture characteristics.

On PDA, colonies are initially white on the surface, gradually turning greyish purple in the center at later stages; the reverse is pale yellow, with lighter coloration at the margins. Mycelium is velvety, and growth is slow, reaching approximately 35 mm in diameter after 14 days at 25 °C. Colonies display distinct concentric rings. On 1/4 SDAY, colony growth is also slow, with the surface initially white and later developing grayish purple patches at the margins, the reverse remains pale yellow.

##### Host.

spider (*Araneae*).

##### Additional material examined.

China • Anhui Province, Chizhou City, Shitai County, Guniujiang Scenic Area (30°04'N, 117°29'E), on a dead spider, August 2020; Ting Wang and Mingjun Chen, GNJ20200814-05. China • Guangdong Province, Shenzhen City, Nanling County (22°36'N, 114°08'E), on a dead spider, August 2021; Qianle Lu, NL20210822-14.

##### Note.

Phylogenetic analyses have shown that *P. araneicola* forms a distinct and well-supported clade (MLBS = 98, BPP = 1.0), indicating a close relationship with *P. atypicola* and *P. atlanticum*. In addition, single-locus phylogenies based on rDNA (ITS+nrLSU), *TEF1*, *RPB1* and *RPB2* showed genealogical concordance, each supporting the monophyly of *P. araneicola*, consistent with GCPSR (Suppl. material 3: fig. S8). A BLASTN search of the GenBank database revealed substantial sequence divergence between the type strain of *P. araneicola* and *P. atypicola* CBS744.73, with identities of 95.44% for nrLSU, 93.90% for *TEF1* and 86.20% for *RPB1*. And the nrLSU sequence of *P. araneicola* showed 95.69% identity with that of *P. atlanticum* FLOR73173. Morphologically, *P. araneicola* is distinguished from *P. atypicola* by having its notably longer conidiophores, measuring 50–186 × 2.2–4.5 μm, compared to 6.0–12.0 × 2–3 μm in *P. atypicola*, and slightly broader conidia, measuring 4.5–8.2 × 2.5–5.3 μm vs. 3.5–8.0 × 1.5–3 μm. *P. araneicola* was distinguished from *P. atlanticum* by its larger conidia [ellipsoidal to ovoid, 4.5–8.2 × 2.5–5.3 μm vs cylindrical, (3.49–)4.3–6.37(–6.6) × (1.6–)1.68–2.44(–2.7) μm]. *Purpureocillium* species display diverse ecological strategies and host associations, engaging in saprobic, symbiotic, or parasitic interactions with a wide range of organisms (Ban et al. 2015a; Figueredo et al. 2020; Calvillo‐Medina et al. 2021; Zhang et al. 2022; Ferreira-Machado et al. 2023; Chang et al. 2024b; Chen et al. 2024b). To date, with the exception of *P. atypicola* and *P. atlanticum*, only *P. lilacinum* has been documented infecting spiders, based on a single record from Argentina (Manfrino et al. 2017). Notably, our study identifies an additional spider-associated lineage within this genus through the description of *P. araneicola*, a sister species to *P. atypicola* and *P. atlanticum* that can be readily distinguished based on molecular data. All three species exhibit a spider-parasitic lifestyle, suggesting a strong host association and ecological similarity among phylogenetically close taxa.

### Divergence time estimation

To explore the evolution, origin, and diversification history of spider-pathogenic fungal lineages, we conducted phylogenetic analyses using a concatenated nucleotide sequence dataset from five gene regions: ITS, nrLSU, *TEF1*, *RPB1* and *RPB2*. The dataset encompasses the majority of known species within the families *Cordycipitaceae*, *Ophiocordycipitaceae* and *Clavicipitaceae*, as well as other related groups. It includes 31 newly sequenced species from this study, 617 *Sordariomycetes* species sourced from public databases and eight *Leotiomycetes* species used as outgroups (See Suppl. material 2).

**Figure 13.**
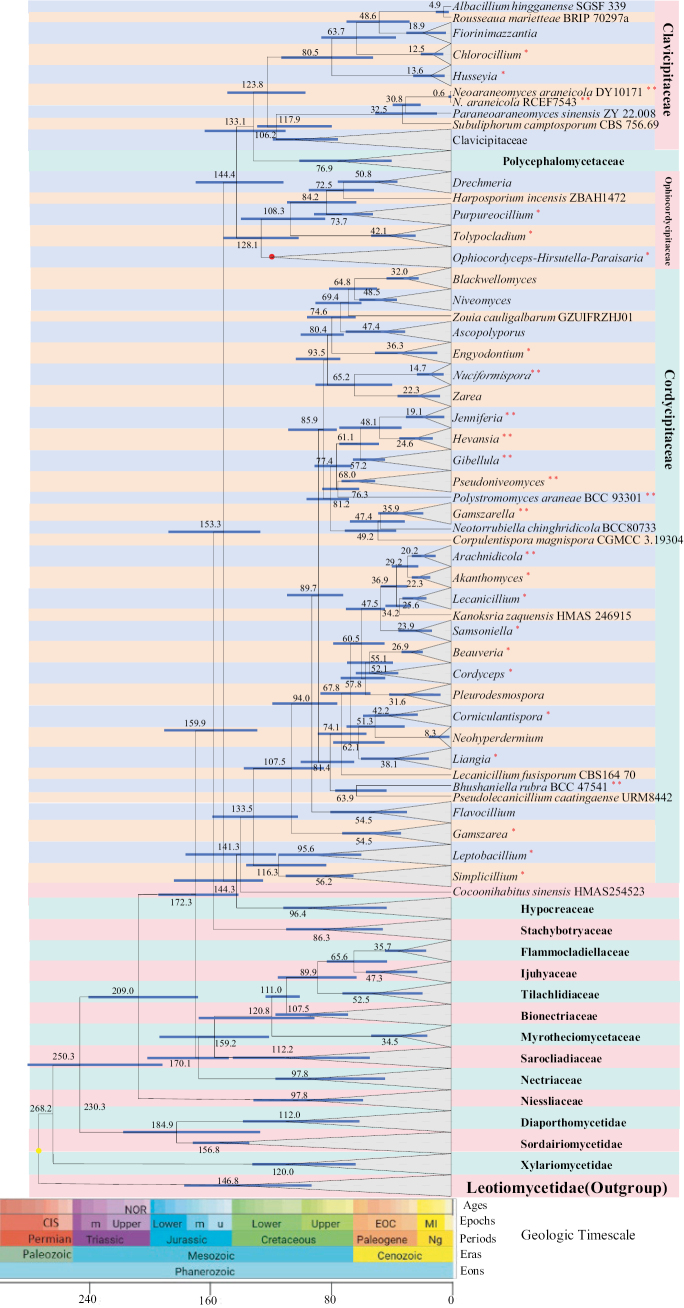
Molecular clock analysis based on a five-locus dataset to infer the divergence times of major clades in the *Hypocreales*. The 95% highest posterior density (HPD) of estimated divergence times, represented by horizontal blue bars, is scaled in millions of years (Mya). “*” indicates that the clade contains spider pathogenic fungi, and “**” indicates that the clade consists of obligate spider pathogenic fungi or is almost entirely composed of spider pathogenic fungi.

The concatenated alignment contains 3,582 nucleotide positions partitioned as follows: ITS (1–468), nrLSU (469–1,258), *TEF1* (1,259–2,136), *RPB1* (2,137–3,307), and *RPB2* (3,308–3,582). We identified a total of 3,093 unique site patterns including 2,223 parsimony-informative sites, 530 singleton sites and 829 constant sites. The best-fit substitution model selected for BEAST analysis was GTR+I+G. A ML tree was constructed to serve as the starting tree for the analysis. The inferred topologies were highly consistent and the final result is presented as a maximum clade credibility (MCC) tree (Fig. 13).

The MCC tree revealed that the order *Hypocreales* comprises 15 well-resolved families: *Bionectriaceae*, *Clavicipitaceae*, *Cordycipitaceae*, *Hypocreaceae*, *Nectriaceae*, *Ophiocordycipitaceae*, *Polycephalomycetaceae*, *Stachybotryaceae*, *Cocoonihabitaceae*, *Flammocladiellaceae*, *Ijuhyaceae*, *Myrotheciomycetaceae*, *Niessliaceae*, *Sarocladiaceae* and *Tilachlidiaceae* (for detailed divergence times, see Table 7). The origin of *Hypocreales* was estimated to date back to the Late Triassic (250.3 Mya), with a crown age in the Middle Jurassic (209 Mya, 95% HPD: 160–259 Mya). These families represent distinct evolutionary trajectories within *Hypocreales*, with varying ecological strategies and host affiliations.

**Table 7.** Estimation of divergence time based on five-locus concatenated molecular clock analysis.

Taxon	Crown age (Mya)	Stem age (Mya)	Taxon	Crown age (Mya)	Stem age (Mya)
* Akanthomyces *	22.3	29.2	* Jenniferia *	19.1	48.1
* Arachnidicola *	20.2	29.2	* Kanoksria *	\	34.2
* Ascopolyporus *	47.4	74.6	* Lecanicillium *	25.6	34.2
* Beauveria *	26.9	55.1	* Leptobacillium *	95.6	116.3
* Bhushaniella *	\	63.9	* Liangia *	38.1	62.1
* Bionectriaceae *	107.5	111	* Myrotheciomycetaceae *	34.5	120.8
* Blackwellomyces *	32	64.8	* Nectriaceae *	97.8	170.1
* Chlorocillium *	12.5	63.7	* Neoaraneomyces *	0.6	30.8
* Clavicipitaceae *	123.8	133.1	* Neohyperdermium *	8.3	51.3
* Cocoonihabitaceae *	\	141.3	* Neotorrubiella *	\	47.4
* Cordycipitaceae *	133.5	141.3	* Niessliaceae *	97.8	209
* Cordyceps *	52.1	55.1	* Niveomyces *	48.5	64.8
* Corniculantispora *	42.2	51.3	* Nuciformispora *	14.7	62.5
* Corpulentispora *	\	49.2	* Ophiocordycipitaceae *	128.1	144.4
* Engyodontium *	36.3	80.4	* Pleurodesmospora *	31.6	57.8
* Flammocladiellaceae *	35.7	65.6	* Polycephalomycetaceae *	76.9	133.1
* Flavocillium *	54.5	94	* Polystromomyces *	\	76.3
* Gamszarea *	54.5	107.5	* Pseudoniveomyces *	68	76.3
* Gamszarella *	35.9	47.4	* Purpureocillium *	73.7	84.2
* Gibellula *	57.2	61.1	* Samsoniella *	23.9	47.5
* Hevansia *	24.6	48.1	* Sarocladiaceae *	112.2	159.2
* Husseyia *	13.6	80.5	* Simplicillium *	56.2	116.3
* Hypocreaceae *	96.4	144.3	* Tolypocladium *	42.1	108.3
* Hypocreales *	250.3	209	* Zarea *	22.3	65.2
* Ijuhyaceae *	47.3	65.6	* Zouia *	\	69.4

Among these lineages, *Niessliaceae* which is associated with plant-based nutrition, represents the earliest-diverging family. It was subsequently followed by the divergence of *Sarocladiaceae*, *Stachybotryaceae* and *Nectriaceae* which are predominantly plant endophytes or saprotrophs. These clades continued evolving along a plant-associated trajectory and eventually gave rise to the youngest families in the order, *Ijuhyaceae* and *Flammocladiellaceae*, which originated during the Late Cretaceous (approximately 65.6 Mya). Other lineages adopted alternative ecological strategies. *Hypocreaceae*, predominantly comprising mycoparasitic and saprotrophic fungi, is inferred to have originated in the Late Jurassic (144.3 Mya), with subsequent diversification during the Early Cretaceous (96.4 Mya). *Ophiocordycipitaceae*, representing insect- and nematode-pathogenic fungi, originated around 144.4 Mya and diverged at 128.1 Mya. *Cordycipitaceae*, which largely comprises arthropod-pathogenic fungi, is inferred to have originated in the Late Jurassic (141.3 Mya), with diversification following in the Early Cretaceous (133.5 Mya). Within this family, transitional genera such as *Simplicillium* and *Leptobacillium* retained ancestral traits associated with plant parasitism. Subsequently, a major radiation of insect-pathogenic fungi was initiated around 107.5 Mya.

The diversification of insect-pathogenic fungi preceded that of spider-pathogenic lineages. However, during later stages of evolution, both groups underwent nearly parallel and independent radiations within distinct phylogenetic clades. The spider-pathogenic lineage is estimated to have originated around 77.4 Mya, followed by the emergence of several genera exhibiting lineage-specific host specialization. These include *Polystromomyces* (stem age: 76.3 Mya), *Pseudoniveomyces* (stem: 76.3 Mya; crown: 68.0 Mya), *Gibellula* (stem: 61.1 Mya; crown: 57.2 Mya), *Hevansia* (stem: 48.1 Mya; crown: 24.6 Mya) and *Jenniferia* (stem: 48.1 Mya; crown: 19.1 Mya). These genera exhibit high host specificity and are almost exclusively associated with spiders. Furthermore, the genus *Arachnidicola*, which consists solely of spider-pathogenic species, originated from a *Samsoniella*-like ancestor around 29.2 Mya. Within *Cordycipitaceae*, several genera with broader host ranges also gave rise to spider-parasitic species in the later stages of diversification. In *Ophiocordycipitaceae*, *Purpureocillium* diversified into obligate spider-pathogenic species, including *P. atypicola* and *P. araneicola*, around 35 Mya. *Clavicipitaceae*, thought to have originated from arthropod-associated ancestors, later underwent a host shift toward plant parasitism, while retaining some insect-associated lineages. The diversification of spider-pathogenic fungi within *Clavicipitaceae* occurred during the later phases of its evolution, with crown ages of *Chlorocillium* and *Husseyia* estimated at 12.5 Mya and 13.6 Mya, respectively, during the Miocene.

Collectively, these findings indicate that the diversification of obligate spider-pathogenic fungi did not stem from a single ancestral lineage. Instead, various insect-pathogenic and plant-endophytic fungi independently acquired the capacity to parasitize spiders in subsequent evolutionary phases. While many entomopathogenic fungi display broad host ranges, spider-pathogenic lineages demonstrate a clear evolutionary trajectory towards greater host specificity.

## Discussion

### Limitations of taxonomy and host specificity

Our comprehensive sampling across multiple Chinese provinces yielded substantial taxonomic novelties, including eight new species: *Arachnidicola anhuiensis*, *Gamszarella araneae*, *Gibellula alba*, *Gibellula guizhouensis*, *Hevansia pseudonelumboides*, *Husseyia sinensis*, *Liangia guizhouensis* and *Purpureocillium araneicola*. Additionally, we propose six new combinations and document *Corniculantispora margaretspencerae* as a new record for China. Notably, some of these species, such as *A. anhuiensis* and *L. guizhouensis*, are currently represented by single strains, which may not fully capture intraspecific morphological or genetic variation. For *G. alba*, although the sexual morph was successfully observed, no culture could be obtained and the asexual morph could not be clearly examined.

The successful acquisition of living cultures for several new species required repeated field surveys and intensive isolation efforts. Beyond the species formally described, our collections included specimens likely representing five undescribed taxa, designated here as *Gibellula* sp. XYC-2025e, *Gibellula* sp. XYC-2025f, *Gibellula* sp. TW-2025a, *Chlorocillium* sp. XYC-2025d, and *Husseyia* sp. XYC-2025g. As these putative species lack stable morphological differences from known taxa, they are not formally described here. As more collections become available, reliable morphological characteristics may be identified, potentially leading to the formal description of these species. This conservative treatment was adopted to avoid premature taxonomic inflation and to ensure that new species are proposed only when sufficient diagnostic evidence is available.

Most spider-pathogenic fungi are difficult to culture in vitro because of their specialized growth requirements, and some historical type specimens lack morphological or molecular diagnostic features, which has complicated taxonomy and species identification (Samson and Evans 1973; Chen et al. 2022a; Mendes-Pereira et al. 2023). The reliance on single or few specimens is therefore sometimes unavoidable when dealing with obligate and highly host-specific fungi. This may be attributed to their high degree of host specificity, as observed in genera such as *Gibellula*, *Hevansia*, *Jenniferia* and *Polystromomyces* (Kuephadungphan et al. 2022; Mongkolsamrit et al. 2022), which makes it challenging to reproduce their on-host growth conditions in vitro. Conventional culture media often lack certain essential nutrients or environmental factors, leading to failure in isolating viable cultures (Vartoukian 2016).

Nevertheless, cultures remain important in both traditional and modern taxonomy. Features such as culture morphology, appressoria formation, and asexual morph production provide valuable characters for species identification (Wijayawardene et al. 2021). However, for spider-pathogenic fungi, taxonomic work relying solely on cultured material is often impractical. In this study, taxa represented by single strains are supported by stable morphological characters and robust multi-locus phylogenetic placement. These taxa should be regarded as testable species hypotheses, open to reassessment as additional collections become available. Future research should focus on improving isolation and cultivation techniques for spider-pathogenic fungi to clarify species boundaries more effectively.

### Significant species diversity of spider-pathogenic fungi

In summary, this study introduces several new taxa that expand the known diversity of spider-pathogenic fungi. Previous estimates of the group’s diversity have likely underestimated its true scale. Shrestha et al. (2019) documented 86 spider-pathogenic species within 13 genera of *Hypocreales*, primarily distributed across *Cordycipitaceae* (8 genera, 75 species), *Ophiocordycipitaceae* (4 genera, 10 species) and *Clavicipitaceae* (1 genus, 1 species). Wang et al. (2024a) updated these numbers to 119 species across 18 genera, including one *Chlorocillium* species (incertae sedis). Subsequently, Chen et al. (2025b) added two species from the *Clavicipitaceae* and noted that, with the placement of *Chlorocillium* in *Clavicipitaceae*, more spider-pathogenic fungi now fall under this family. Nyffeler and Hywel-Jones (2024) also highlighted that the actual number of spider-pathogenic fungi is likely far greater than previously recognized. By integrating our newly isolated taxa with published global records, we provide an updated framework indicating that spider-pathogenic fungi currently comprise 181 species in 35 genera across four families of *Hypocreales* (including unpublished data from this study). In China, 79 species in 22 genera and four families have been reported to date. Notably, China, as one of the world’s biodiversity hotspots with highly diverse topography and climatic conditions, does not yet exhibit markedly higher recorded diversity of spider-pathogenic fungi than countries such as Japan and Thailand, suggesting that many species remain undiscovered and untapped within its territory. Despite the group’s substantial diversity, it remains underexplored and underrepresented in broader research efforts. As new species continue to be discovered, we anticipate a significant increase in recognized diversity, which may rekindle interest among mycologists, arachnologists, and related specialists, and ultimately support the scientific development and application of these ecologically important fungi.

### Origin and evolution of spider-pathogenic fungi

In our molecular clock analyses, we applied a Calibrated Yule model as the tree prior. The Yule model is a simple and widely used speciation model that assumes constant diversification rates and no extinction, making it generally appropriate for analyzing sequences from multiple species across genera, such as in our multi-locus dataset. While real fungal lineages, including spider-pathogenic *Hypocreales*, may exhibit heterogeneous diversification and non-zero extinction, the Yule model provides a practical and computationally tractable framework for estimating divergence times across a large phylogeny (Heled and Drummond 2011; Wei et al. 2022). Its assumptions should be considered when interpreting absolute ages, particularly for deeper nodes, and future analyses could explore more complex birth–death models, which explicitly account for variable speciation and extinction rates, to evaluate the robustness of divergence-time estimates under alternative tree priors. Using this framework, we investigated the evolutionary history of spider-pathogenic fungi based on a concatenated five-locus dataset (ITS, nrLSU, *TEF1*, *RPB1*, *RPB2*) from 648 strains representing most known taxa in *Cordycipitaceae*, *Ophiocordycipitaceae*, and *Clavicipitaceae*. The MCC tree resolved *Hypocreales* into 15 well-supported families, with stem and crown ages estimated at 250.2 Mya (Late Triassic) and 209 Mya (Middle Jurassic). Previous studies have repeatedly estimated the divergence times of *Hypocreales* (Table 8) and our results are broadly consistent with those of Wei et al. (2022), who reported 240 Mya and 205 Mya, respectively. *Niessliaceae* was the earliest diverging lineage, followed by endophytic or saprophytic families (*Sarocladiaceae*, *Stachybotryaceae*, *Nectriaceae*), while *Ijuhyaceae* and *Flammocladiellaceae* were the youngest (65.6 Mya), though Sun et al. (2023) suggested divergence ages of most families between 116–159 Mya, warranting further taxonomic review. *Cordycipitaceae*, a major insect-pathogenic lineage, originated in the late Jurassic (141.3 Mya) and diversified in the Early Cretaceous (133.5 Mya), with transitional genera (*Simplicillium*, *Leptobacillium*) retaining plant-associated traits (Chang et al. 2024a). This shift from plant- to arthropod-associated parasitism around 107.5 Mya highlights the role of ecological opportunity in driving evolutionary innovation. Spider-pathogenic fungi likely originated later (77.4 Mya), coinciding with angiosperm-driven diversification of insects and spiders, including the radiation of modern families such as *Araneidae* and *Salticidae* (100–80 Mya), supporting a co-evolutionary model (Shao and Li 2018; Wang et al. 2025). The earlier divergence of insect pathogens likely reflects the earlier adaptive radiation of insects, while spider defenses (exoskeletons, immune responses, behaviors) may have delayed fungal adaptation, leading to obligate spider-associated genera such as *Polystromomyces*, *Pseudoniveomyces*, *Gibellula*, *Hevansia* and *Jenniferia*. These genera likely evolved through long-term host specialization, with slower evolutionary rates than broad-host-range insect pathogens. Whether virulence genes were acquired via horizontal gene transfer from insect pathogens remains unknown. Arthropod-pathogenic fungi range from specialists to generalists (Vega et al. 2012); specialists excel in low-diversity host environments (Woolhouse et al. 2001), while generalists thrive in diverse ecosystems (Hajek et al. 2022; Sacco and Hajek 2023). We hypothesize that spider pathogens adopted high-efficiency parasitism under limited host resources, evolving adaptations to spider cuticles, immunity and behaviors to occupy distinct niches and reduce competition. In *Ophiocordycipitaceae*, specialized spider pathogens such as *P. atypicola* and *P. araneicola* diverged approximately 35 Mya, while in *Clavicipitaceae*, the spider-pathogenic genera *Chlorocillium* and *Husseyia* originated around 12.5 and 13.6 Mya, respectively. Some generalist entomopathogens can also infect spiders (Savić et al. 2016; Chen et al. 2017; Manfrino et al. 2017), reflecting diverse adaptive pathways. Overall, these patterns underscore the roles of co-evolution, niche differentiation, and genetic adaptation in the diversification of spider-pathogenic fungi.

**Table 8.** Estimated divergence times of *Hypocreales*.

Data Source	Stem age (Mya)	Crown Age (Mya)
This study	250.2	209
Bao et al. (2023)	274	222
Hyde et al. (2017)	231	/
Hyde et al. (2020)	229	/
Ren et al. (2021)	/	225 or 228
Samarakoon et al. (2016)	200	163
Sun et al. (2023)	/	206
Sung et al. (2008)	/	193
Wei et al. (2022)	250	200

### Biotechnological potential and applied significance

Spider-pathogenic fungi represent an underexplored reservoir of bioactive compounds with pharmaceutical applications. Previous studies have identified numerous secondary metabolites with antimicrobial, antiviral and immunomodulatory properties (Isaka et al. 2013; Isaka et al. 2014; Kuephadungphan et al. 2014). The taxonomic expansion documented in this study substantially increases the available genetic resources for bioprospecting and biotechnological development.

Furthermore, the ecological specialization observed in spider-pathogenic fungi suggests highly refined metabolic pathways adapted to overcome spider-specific immune defenses. Understanding these biochemical adaptations may provide insights into novel bioactive compounds and mechanisms of host-pathogen interaction that could be exploited for therapeutic applications.

In conclusion, this study significantly advances our understanding of spider-pathogenic fungal diversity through the description of numerous taxonomic novelties and phylogenetic reassessments. The description of several new taxonomic entities substantially expands known taxonomic diversity while providing robust molecular frameworks for future systematic research. Our molecular clock analysis reveals complex evolutionary patterns involving multiple independent origins of spider parasitism and progressive specialization toward host-specific ecological strategies.

Future research should integrate multi-omics approaches with comprehensive field surveys to fully characterize the molecular mechanisms underlying host specialization and the biogeographic factors shaping species distributions. Such integrative approaches will be essential for realizing the full biotechnological potential of these ecologically significant fungi while contributing to broader understanding of fungal evolution and biodiversity patterns.

## Supplementary Material

XML Treatment for Arachnidicola anhuiensis

XML Treatment for Gamszarella araneae

XML Treatment for Gamszarella sotirae

XML Treatment for Corniculantispora margaretspencerae

XML Treatment for Corniculantispora saksenae

XML Treatment for Gibellula alba

XML Treatment for Gibellula guizhouensis

XML Treatment for Hevansia pseudonelumboides

XML Treatment for Husseyia sinensis

XML Treatment for Liangia guizhouensis

XML Treatment for Nuciformispora araneae

XML Treatment for Nuciformispora rasoulzarei

XML Treatment for Nuciformispora spenceae

XML Treatment for Purpureocillium araneicola

## Additional information

### Use of AI

No use of AI was reported.

### Adherence to national and international regulations

All the fungal strains used in this study have been legally obtained, respecting the Convention on Biological Diversity (Rio Convention).

### Author ORCIDs

Xiao-Yun Chang https://orcid.org/0000-0002-0093-9582

Yan-Jia Qi https://orcid.org/0009-0001-9815-7369

Ting Wang https://orcid.org/0000-0002-9296-7280

Zeng-Zhi Li https://orcid.org/0000-0002-9606-5030

Guang-Shuo Li https://orcid.org/0000-0002-7285-0712

Bo Huang https://orcid.org/0000-0001-6032-7396

Ming-Jun Chen https://orcid.org/0000-0002-1439-7796

### Data availability

All sequence data generated for this study can be accessed via GenBank: https://www.ncbi.nlm.nih.gov/genbank/.

## Supplementary materials

Supplementary material 1 Sampling locations, meteorological data, and alignment trimming statistics Data type xlsx Explanation note **figure S1**. Schematic map of sampling locations in this study. Red dots indicate sampling coordinates. **table SS1**. Sampling dates of all strains examined in this study and corresponding daily meteorological data. **table SS2**. Summary of alignment trimming statistics for phylogenetic analyses. https://binary.pensoft.net/file/1585501This dataset is made available under the Open Database License (http://opendatacommons.org/licenses/odbl/1.0/). The Open Database License (ODbL) is a license agreement intended to allow users to freely share, modify, and use this Dataset while maintaining this same freedom for others, provided that the original source and author(s) are credited. Xiao-Yun Chang, Yan-Jia Qi, Ming-Jun Chen

Supplementary material 2 GenBank accession numbersr for molecular clock analysis Data type docx Explanation note **table SS3**. Species information used for molecular clock analysis. Species names, strain numbers, GenBank accession numbers, and hosts. https://binary.pensoft.net/file/1585502This dataset is made available under the Open Database License (http://opendatacommons.org/licenses/odbl/1.0/). The Open Database License (ODbL) is a license agreement intended to allow users to freely share, modify, and use this Dataset while maintaining this same freedom for others, provided that the original source and author(s) are credited. Xiao-Yun Chang, Yan-Jia Qi, Ming-Jun Chen

Supplementary material 3 Single-gene phylogenetic tree of the new species Data type docx Explanation note **figure S2**. Single-gene phylogenetic trees illustrating the phylogenetic relationships among species within the genus *Arachnidicola*. **figure S3**. Single-gene phylogenetic trees illustrating the phylogenetic relationships among species within the genus *Gamszarella*. **figure S4**. Single-gene phylogenetic trees illustrating the phylogenetic relationships among species within the genus *Gibellula*. **figure S5**. Single-gene phylogenetic trees illustrating the phylogenetic relationships among species within the genus *Hevansia*. **figure S6**. Single-gene phylogenetic trees illustrating the phylogenetic relationships among species within the genus *Chlorocillium* and *Husseyia*. **figure S7**. Single-gene phylogenetic trees illustrating the phylogenetic relationships among species within the genus *Liangia*. **figure S8**. Single-gene phylogenetic trees illustrating the phylogenetic relationships among species within the genus *Purpureocillium*. **table SS4**. Genealogical concordance among single-locus phylogenies supporting species delimitation under GCPSR. https://binary.pensoft.net/file/1585503This dataset is made available under the Open Database License (http://opendatacommons.org/licenses/odbl/1.0/). The Open Database License (ODbL) is a license agreement intended to allow users to freely share, modify, and use this Dataset while maintaining this same freedom for others, provided that the original source and author(s) are credited. Xiao-Yun Chang, Yan-Jia Qi, Ming-Jun Chen

Supplementary material 4 Alignment used for constructing the phylogenetic tree shown in figure 1 Data type phy Explanation note Concatenated sequence alignment of five genes (ITS, nrLSU, *TEF1*, *RPB1*, and *RPB2*) used for reconstructing the phylogenetic tree in Fig. 1. https://binary.pensoft.net/file/1585504This dataset is made available under the Open Database License (http://opendatacommons.org/licenses/odbl/1.0/). The Open Database License (ODbL) is a license agreement intended to allow users to freely share, modify, and use this Dataset while maintaining this same freedom for others, provided that the original source and author(s) are credited. Xiao-Yun Chang, Yan-Jia Qi, Ming-Jun Chen

Supplementary material 5 Alignment used for constructing the phylogenetic tree shown in Fig. 2 Data type phy Explanation note Concatenated sequence alignment of five genes (ITS, nrLSU, *TEF1*, *RPB1*, and *RPB2*) used for reconstructing the phylogenetic tree in Fig. 2. https://binary.pensoft.net/file/1585505This dataset is made available under the Open Database License (http://opendatacommons.org/licenses/odbl/1.0/). The Open Database License (ODbL) is a license agreement intended to allow users to freely share, modify, and use this Dataset while maintaining this same freedom for others, provided that the original source and author(s) are credited. Xiao-Yun Chang, Yan-Jia Qi, Ming-Jun Chen

Supplementary material 6 Alignment used for constructing the phylogenetic tree shown in figure 3 Data type phy Explanation note Concatenated sequence alignment of five genes (ITS, nrLSU, *TEF1*, *RPB1*, and *RPB2*) used for reconstructing the phylogenetic tree in Fig. 3. https://binary.pensoft.net/file/1585506This dataset is made available under the Open Database License (http://opendatacommons.org/licenses/odbl/1.0/). The Open Database License (ODbL) is a license agreement intended to allow users to freely share, modify, and use this Dataset while maintaining this same freedom for others, provided that the original source and author(s) are credited. Xiao-Yun Chang, Yan-Jia Qi, Ming-Jun Chen

Supplementary material 7 Alignment sequences for molecular clock analysis Data type phy Explanation note The concatenated sequence alignment data of five genes (ITS, nrLSU, *TEF1*, *RPB1*, and *RPB2*) used for molecular clock analysis. https://binary.pensoft.net/file/1585507This dataset is made available under the Open Database License (http://opendatacommons.org/licenses/odbl/1.0/). The Open Database License (ODbL) is a license agreement intended to allow users to freely share, modify, and use this Dataset while maintaining this same freedom for others, provided that the original source and author(s) are credited. Xiao-Yun Chang, Yan-Jia Qi, Ming-Jun Chen
