# Supplementary material for: Taxonomy, phylogeny, and evolutionary diversification of spider-pathogenic fungi from China (Hypocreales, Ascomycota)
Source: IMA Fungus. 2026 Apr 6;17:e171548. doi: 10.3897/imafungus.17.171548 (PMC13077317; doi:10.3897/imafungus.17.171548)
Supplement: Supplementary material 2 — GenBank accession numbersr for molecular clock analysis [file imafungus-17-e171548-s002.docx]

Table S3. Species information used for molecular clock analysis. Species names, strain numbers, GenBank accession numbers, and hosts.

| Subclass | Order | Family | Species | Strain code | GenBank accession number | | | | | Host | References |
| --- | --- | --- | --- | --- | --- | --- | --- | --- | --- | --- | --- |
|  |  |  |  |  | ITS | *TEF1* | nrLSU | *RPB1* | *RPB2* |  |  |
| Leotiomycetidae | Helotiales | Cenangiaceae | *Cenangiopsis quercicola* | KL174 | LT158425 | KX090663 | KX090811 | KX090760 | KX090713 | *Quercus robur* | Beenken et al. (2023) |
| Leotiomycetidae | Helotiales | Mollisiaceae | *Mollisia cinerea* | AFTOL-ID 76 | DQ491498 | DQ471051 | DQ470942 | DQ471122 | DQ470883 | Fallen log | Pärtel et al. (2017) |
| Leotiomycetidae | Helotiales | Rutstroemiaceae | *Rutstroemia tiliacea* | KL160 | LT158423 | KX090661 | KX090808 | KX090757 | KX090711 | Tilia sp. | Pärtel et al. (2017) |
| Leotiomycetidae | Helotiales | Sclerotiniaceae | *Pycnopeziza sejournei* | KL267 | LT158443 | KX090679 | KX090827 | KX090772 | KX090726 | *Hedera helix* | Pärtel et al. (2017) |
| Leotiomycetidae | Hypocreales | Ophiocordycipitaceae | *Drechmeria balanoides* | CBS 250.82 | NR_155044 | DQ522342 | NG_059128 | DQ522388 | DQ522442 | Nematoda | Spatafora et al. (2007) |
| Leotiomycetidae | Leotiales | Leotiaceae | *Leotia lubrica* | AFTOL-ID 1 | DQ491484 | DQ471041 | AY544644 | DQ471113 | DQ470876 | Woodland | Pärtel et al. (2017) |
| Leotiomycetidae | Rhytismatales | Rhytismataceae | *Coccomyces dentatus* | AFTOL-ID 147 | DQ491499 | DQ497605 | AY544657 | - | DQ247789 | Plant | Karakehian et al. (2019) |
| Leotiomycetidae | Thelebolales | Thelebolaceae | *Thelebolus globosus* | AFTOL-ID 5016 | MH862951 | FJ238418 | FJ176905 | FJ238446 | FJ238385 | Lake biomats | Schoch et al. (2009) |
| Diaporthomycetidae | Diaporthales | Melanconidaceae | *Melanconis stilbostoma* | AFTOL-ID 936 | DQ323524 | DQ836910 | FJ713618 | FJ238429 | DQ836890 | *Betula pendula* | Schoch et al. (2009) |
| Diaporthomycetidae | Helotiales | Mollisiaceae | *Mollisia ventosa* | TNS:F:86243 | LC754415 | LC756242 | LC754441 | LC754470 | LC756210 | Tilia sp. | Unpublished |
| Diaporthomycetidae | Phyllachorales | Phyllachoraceae | *Apiosphaeria guaranitica* | UB23393 | MG573329 | MG573320 | - | - | MG573312 | *Handroanthus ochraceus* | Guterres et al. (2018) |
| Hypocreomycetidae | Hypocreales | Bionectriaceae | *Acremonium alternatum* | CBS 407.66 | OQ429442 | OQ470739 | OQ055353 | - | OQ560696 | *Hypoxylon deustum* | Hou et al. (2023) |
| Hypocreomycetidae | Hypocreales | Bionectriaceae | *Acremonium brachypenium* | CBS 866.73 | OQ429443 | OQ470740 | OQ055354 | - | OQ453837 | *Cocos nucifera* | Hou et al. (2023) |
| Hypocreomycetidae | Hypocreales | Bionectriaceae | *Bulbithecium truncatum* | CBS 113718 | OQ429513 | OQ470803 | OQ055424 | - | OQ453900 | *Vachellia nubica* | Hou et al. (2023) |
| Hypocreomycetidae | Hypocreales | Bionectriaceae | *Clonostachys aranearum* | GZAC QLS0625clo | KU173835 | - | - | - | - | Araneae: spider | Wang et al. (2023a) |
| Hypocreomycetidae | Hypocreales | Bionectriaceae | *Clonostachys australiana* | CBS 102421 | OQ910540 | OQ944554 | OQ910899 | - | OQ927618 | Bark of recently dead tree | Zhao et al. (2023) |
| Hypocreomycetidae | Hypocreales | Bionectriaceae | *Clonostachys buxi* | CBS 696.93 | KM231840 | KM231977 | KM231721 | KM232273 | KM232416 | Leaves of Buxus sempervirens | Wang et al. (2023a) |
| Hypocreomycetidae | Hypocreales | Bionectriaceae | *Clonostachys chloroleuca* | CBS 141588 l | KC806286 | KX184988 | OQ910908 | KX184923 | OQ927627 | Soil | Wang et al. (2023a) |
| Hypocreomycetidae | Hypocreales | Bionectriaceae | *Clonostachys chuyangsinensis* | YFCC 896 | MW199067 | MW295967 | MW199056 | - | - | Araneae: spider | Wang et al. (2023a) |
| Hypocreomycetidae | Hypocreales | Bionectriaceae | *Clonostachys fujianensis* | CBS 127474 | OQ910620 | OQ944632 | OQ910979 | - | OQ927691 | Bamboo stem | Zhao et al. (2023) |
| Hypocreomycetidae | Hypocreales | Bionectriaceae | *Clonostachys fusca* | CBS 207.93 | OQ910622 | OQ944634 | OQ910981 | - | OQ927693 | Herbaceous stem | Zhao et al. (2023) |
| Hypocreomycetidae | Hypocreales | Bionectriaceae | *Clonostachys lucifer* | CBS 100008 | OQ910644 | OQ944656 | OQ911003 | - | OQ927713 | *Casicena arborea* | Zhao et al. (2023) |
| Hypocreomycetidae | Hypocreales | Bionectriaceae | *Clonostachys moreaui* | CBS 127881 | OQ910647 | OQ944659 | OQ911006 | - | OQ927716 | *Laurus novocanariensis* | Zhao et al. (2023) |
| Hypocreomycetidae | Hypocreales | Bionectriaceae | *Clonostachys oblongispora* | CBS 100285 | OQ910648 | OQ944660 | OQ911007 | - | OQ927717 | *Orixa japonica* | Zhao et al. (2023) |
| Hypocreomycetidae | Hypocreales | Bionectriaceae | *Clonostachys parasporodochialis* | CBS 192.93 | OQ910651 | OQ944663 | OQ911010 | - | OQ927720 | Terminal branchlet of recently dead tree | Zhao et al. (2023) |
| Hypocreomycetidae | Hypocreales | Bionectriaceae | *Clonostachys pityrodes* | CBS 102033 | OQ910655 | OQ944667 | OQ911014 | - | OQ927723 | Bark | Zhao et al. (2023) |
| Hypocreomycetidae | Hypocreales | Bionectriaceae | *Clonostachys rhizophaga* | CBS 202.37 | OQ910694 | OQ944706 | OQ911053 | - | OQ927762 | *Ulmus americana* | Zhao et al. (2023) |
| Hypocreomycetidae | Hypocreales | Bionectriaceae | *Clonostachys samuelsii* | CBS 699.97 | OQ910812 | OQ944822 | OQ911171 | - | OQ927878 | Bark of tree | Zhao et al. (2023) |
| Hypocreomycetidae | Hypocreales | Bionectriaceae | *Clonostachys subquaternata* | CBS 100003 | OQ910865 | OQ944874 | OQ911224 | - | OQ927928 | *Casicena arborea* | Zhao et al. (2023) |
| Hypocreomycetidae | Hypocreales | Bionectriaceae | *Emericellopsis alkalina* | CBS 127350 | MH864534 | *KC998993* | MH875970 | - | KC999029 | Soda soil | Hou et al. (2023) |
| Hypocreomycetidae | Hypocreales | Bionectriaceae | *Emericellopsis terricola* | CBS 120.40 | MH856058 | *OQ470890* | MH867553 | - | OQ453980 | Soil | Hou et al. (2023) |
| Hypocreomycetidae | Hypocreales | Bionectriaceae | *Fusariella arenula* | CBS 329.77 | OQ429593 | OQ470903 | OQ055504 | - | OQ453992 | *Phormium tenax* | Hou et al. (2023) |
| Hypocreomycetidae | Hypocreales | Bionectriaceae | *Gliomastix polychroma* | CBS 181.27 | OQ429629 | OQ470931 | OQ055528 | - | OQ454020 | Hevea brasiliensis | Hou et al. (2023) |
| Hypocreomycetidae | Hypocreales | Bionectriaceae | *Hydropisphaera fungicola* | CBS 122304 | OQ429666 | OQ470973 | OR052107 | - | OQ454063 | *Ulocladium atrum* | Hou et al. (2023) |
| Hypocreomycetidae | Hypocreales | Bionectriaceae | *Ovicillium oosporum* | CBS 110151 | OQ429758 | OQ471084 | OQ055657 | - | OQ454169 | *Theobroma gileri* | Hou et al. (2023) |
| Hypocreomycetidae | Hypocreales | Bionectriaceae | *Paracylindrocarpon aurantiacum* | CBS 135909 | OQ429763 | OQ471090 | OQ055662 | - | OQ454175 | Lamium galeobdolon | Hou et al. (2023) |
| Hypocreomycetidae | Hypocreales | Bionectriaceae | *Stilbocrea gracilipes* | SSM-2021081303 | ON041132 | ON125559 | ON041116 | ON081502 | ON081503 | Dead wood | Maharachchikumbura et al. (2022) |
| Hypocreomycetidae | Hypocreales | Bionectriaceae | *Waltergamsia parva* | CBS 831.97 | OQ429947 | OQ471280 | OQ430197 | - | OQ454347 | *Cirsium arvense* | Hou et al. (2023) |
| Hypocreomycetidae | Hypocreales | Clavicipitaceae | *Aciculosporium oplismeni* | MAFF 246966a | LC571760 | LC572040 | LC571760 | - | LC572054 | *Oplismenus undulatifolius* | Tanaka et al. (2021) |
| Hypocreomycetidae | Hypocreales | Clavicipitaceae | *Aciculosporium phalalidis* | CCC 293 | AJ133399 | LT216524 | AJ133399 | - | LT216598 | *Phalaris tuberosa* | Tanaka et al. (2021) |
| Hypocreomycetidae | Hypocreales | Clavicipitaceae | *Aciculosporium sasicola* | TNSF60466 | LC571758 | LC572038 | LC571758 | - | LC572052 | *Sasa senanensis* | Tanaka et al. (2021) |
| Hypocreomycetidae | Hypocreales | Clavicipitaceae | *Aciculosporium sasicola* | MAFF 247297 | LC571759 | LC572039 | LC571759 | - | LC572053 | *Sasa palmata* | Tanaka et al. (2021) |
| Hypocreomycetidae | Hypocreales | Clavicipitaceae | *Aciculosporium take* | MAFF 241224 | LC571753 | LC572034 | LC571753 | - | LC572048 | *Phyllostchys pubescens* | Tanaka et al. (2021) |
| Hypocreomycetidae | Hypocreales | Clavicipitaceae | *Albacillium hingganense* | SGSF 339 | OR740562 | MN065771 | OR740566 | OR769082 | OR769081 | Dead fallen leaves | Ding et al. (2024) |
| Hypocreomycetidae | Hypocreales | Clavicipitaceae | *Aschersonia badia* | BCC 8105 | - | DQ522317 | DQ518752 | DQ522363 | DQ522411 | Insect | Spatafora et al. (2007) |
| Hypocreomycetidae | Hypocreales | Clavicipitaceae | *Aschersonia calendulina* | MFLU:162918 | - | KY646198 | - | KY646199 | KY646197 | Insect | Mongkolsamrit et al. (2009) |
| Hypocreomycetidae | Hypocreales | Clavicipitaceae | *Aschersonia luteola* | SM00098.03 | JN942616 | - | JN940907 | JN987885 | - | Insect | Mongkolsamrit et al. (2009) |
| Hypocreomycetidae | Hypocreales | Clavicipitaceae | *Aschersonia minutispora* | BCC 20635 | EU409583 | DQ552143 | GU552149 | - | - | Insect | Mongkolsamrit et al. (2009) |
| Hypocreomycetidae | Hypocreales | Clavicipitaceae | *Aschersonia samoensis* | BCC 7865 | - | DQ384975 | DQ384946 | DQ384997 | - | Insect | Mongkolsamrit et al. (2009) |
| Hypocreomycetidae | Hypocreales | Clavicipitaceae | *Atkinsonella hypoxylon* | B4728 | U78052 | KP689546 | - | - | KP689514 | *Danthonia spicata* | Lin et al. (2025) |
| Hypocreomycetidae | Hypocreales | Clavicipitaceae | *Balansia claviceps* | CBS:501.70 | MH859816 | - | MH871588 | - | - | *Cyrtococcum oxyphyllum* | Vu et al. (2019) |
| Hypocreomycetidae | Hypocreales | Clavicipitaceae | *Balansia henningsiana* | GAM 16112 | - | AY489610 | AY545727 | - | DQ522413 | Panicum sp. | Spatafora et al. (2007) |
| Hypocreomycetidae | Hypocreales | Clavicipitaceae | *Chlorocillium araneogenum* | DY101741 | MW730532 | MW753037 | MW730618 | - | MW753030 | Araneae: spider | Chen et al. (2025a) |
| Hypocreomycetidae | Hypocreales | Clavicipitaceae | *Chlorocillium griseum* | RCEF4626 | OM843125 | MW091326 | MW084341 | MW091330 | MW091328 | Araneae: spider | Wang et al. (2024a) |
| Hypocreomycetidae | Hypocreales | Clavicipitaceae | *Chlorocillium griseum* | RCEF7541 | PV134479 | PV166479 | PV134538 | PV166543 | PV166578 | Araneae: spider | This study |
| Hypocreomycetidae | Hypocreales | Clavicipitaceae | *Chlorocillium gueriniae* | BRIP 72680a | OR750699 | OR737799 | OR731505 | - | OR737788 | Insect | Chen et al. (2025a) |
| Hypocreomycetidae | Hypocreales | Clavicipitaceae | *Chlorocillium lepidopterorum* | SD05361 | MW730543 | MW753041 | MW730624 | - | - | Lepidoptera pupa | Chen et al. (2025a) |
| Hypocreomycetidae | Hypocreales | Clavicipitaceae | *Chlorocillium mauryae* | MST F26633 | PQ607739 | PQ566634 | - | - | PQ566633 | Insect | Tan et al. (2024) |
| Hypocreomycetidae | Hypocreales | Clavicipitaceae | *Chlorocillium montefioreae* | BRIP 70299a | PP420202 | PP438400 | PP415875 | - | PP438395 | Insect | Chen et al. (2025a) |
| Hypocreomycetidae | Hypocreales | Clavicipitaceae | *Chlorocillium sinense* | KY07181 | PP768154 | PP766580 | PP768156 | - | PP766578 | Araneae: spider | Chen et al. (2025a) |
| Hypocreomycetidae | Hypocreales | Clavicipitaceae | *Chlorocillium sp. XYC-2025d* | RCEF7509 | PV134470 | PV166470 | PV134529 | PV166541 | - | Araneae: spider | This study |
| Hypocreomycetidae | Hypocreales | Clavicipitaceae | *Chlorocillium winlockiae* | MST F3581 | PQ607741 | PQ566637 | PQ607748 | - | PQ566636 | Insect | Tan et al. (2024) |
| Hypocreomycetidae | Hypocreales | Clavicipitaceae | *Claviceps bavariensis* | CCC 503 | JX083498 | JX083705 | - | - | JX083636 | *Ammophila arenaria* | Tanaka et al. (2023) |
| Hypocreomycetidae | Hypocreales | Clavicipitaceae | *Claviceps paspali* | ATCC 13892 | JN049818 | DQ52232 | U17398 | DQ522367 | DQ522416 | *Paspalum dilatatum* | Tanaka et al. (2023) |
| Hypocreomycetidae | Hypocreales | Clavicipitaceae | *Claviceps purpurea* | TNSF96394 | LC681687 | LC684117 | - | - | LC684643 | *Lolium multiflorum* | Tanaka et al. (2023) |
| Hypocreomycetidae | Hypocreales | Clavicipitaceae | *Claviceps ripicola* | DAOMC 251843 | MH477801 | MH397452 | - | - | MH349060 | *Phalaris arundinacea* | Tanaka et al. (2023) |
| Hypocreomycetidae | Hypocreales | Clavicipitaceae | *Commelinaceomyces aneilematis* | MAFF 246963 | LC474614 | LC474623 | LC474617 | LC474626 | LC474629 | Commelinaceae | Lovett et al. (2024) |
| Hypocreomycetidae | Hypocreales | Clavicipitaceae | *Conoideocrella luteorostrata* | ARSEF 114590 | - | OR500274 | - | OR500272 | OR500273 | *Fiorinia externa* | Lovett et al. (2024) |
| Hypocreomycetidae | Hypocreales | Clavicipitaceae | *Conoideocrella tenuis* | BCC 44534 | MG230251 | MG230541 | MG198773 | - | - | Hemiptera sp. | Lovett et al. (2024) |
| Hypocreomycetidae | Hypocreales | Clavicipitaceae | *Echinodothis tuberiformis* | J.F. White | OR162438 | AF276510 | JQ257009 | JQ257015 | JQ257020 | *Arundinaria tecta* | Kepler et al. (2012b) |
| Hypocreomycetidae | Hypocreales | Clavicipitaceae | *Epichloe sylvatica* | HKAS:106462 | OQ127332 | OQ186442 | OQ127367 | OQ186414 | OQ186392 | Stem of Microstegium sp. | Wei et al. (2022) |
| Hypocreomycetidae | Hypocreales | Clavicipitaceae | *Epichloe typhina* | ATCC 56429 | JN049832 | AF543777 | U17396 | AY489653 | DQ522440 | *Dactylis glomerata* | Lin et al. (2025) |
| Hypocreomycetidae | Hypocreales | Clavicipitaceae | *Fiorinimazzantia australiana* | BRIP 70251b | - | OR964979 | OR947081 | - | OR964975 | Insect | Tan and Shivas (2023d) |
| Hypocreomycetidae | Hypocreales | Clavicipitaceae | *Fiorinimazzantia elisabettae* | BRIP 72660a | OR750703 | OR737803 | OR731509 | - | OR737792 | insect | Tan and Shivas (2023d) |
| Hypocreomycetidae | Hypocreales | Clavicipitaceae | *Helicocollum krabiensis* | BCC 71373 | - | KT222341 | KT222326 | KT222334 | - | Hemiptera: scale insect | Luangsa-ard et al. (2017) |
| Hypocreomycetidae | Hypocreales | Clavicipitaceae | *Husseyia annamariae* | BRIP 72654a | OR750704 | OR737804 | OR731510 | - | OR737793 | Insect | Tan and Shivas (2023d) |
| Hypocreomycetidae | Hypocreales | Clavicipitaceae | *Husseyia queenslandica* | BRIP 72669a | OR947074 | - | OR947082 | - | - | Insect | Tan and Shivas (2023c) |
| Hypocreomycetidae | Hypocreales | Clavicipitaceae | *Husseyia sp. XYC-2025g* | LS20230802-70 | PV134520 | PV166531 | - | - | PV166615 | Araneae: spider | This study |
| Hypocreomycetidae | Hypocreales | Clavicipitaceae | *Hypocrella cf discoidea* | I93 901D | - | EU392646 | EU392567 | EU392700 | - | Scale insects or whiteflies | Wang et al. (2024e) |
| Hypocreomycetidae | Hypocreales | Clavicipitaceae | *Hypocrella viridans* | PC 635 | - | EU392651 | EU392572 | EU392705 | - | Scale insects or whiteflies | Wang et al. (2024e) |
| Hypocreomycetidae | Hypocreales | Clavicipitaceae | *Keithomyces carneus* | CBS 239.32 | NR_131993 | EF468789 | NG_057769 | EF468894 | EF468938 | Soil | Mongkolsamrit et al. (2020)a |
| Hypocreomycetidae | Hypocreales | Clavicipitaceae | *Keithomyces neogunnii* | BUM415 | MH143811 | MH143861 | MH143828 | MH143876 | MH143891 | Soil | Mongkolsamrit et al. (2020)a |
| Hypocreomycetidae | Hypocreales | Clavicipitaceae | *Marquandomyces marquandii* | CBS 182.27 | MH854923 | EF468793 | MH866418 | EF468899 | EF468942 | Soil | Chen et al. (2025a) |
| Hypocreomycetidae | Hypocreales | Clavicipitaceae | *Marquandomyces sinensis* | ZY22.065 | OR680544 | OR858938 | OR680611 | OR680906 | OR842959 | Soil | Zhang et al. (2024b) |
| Hypocreomycetidae | Hypocreales | Clavicipitaceae | *Metapochonia bulbillosa* | CBS 145.70 | MH859529 | EF468796 | AF339542 | EF468902 | EF468943 | Root of Picea abies | Hou et al. (2023) |
| Hypocreomycetidae | Hypocreales | Clavicipitaceae | *Metapochonia parasitica* | ARSEF 3436 | FJ973068 | EF468799 | EF468848 | EF468904 | EF468945 | Soil | Sung et al. (2007) |
| Hypocreomycetidae | Hypocreales | Clavicipitaceae | *Metapochonia rubescebs* | CBS 464.88 | MH862138 | EF468797 | MH873830 | EF468903 | EF468944 | Soil | Hou et al. (2023) |
| Hypocreomycetidae | Hypocreales | Clavicipitaceae | *Metapochonia suchlasporia* | CBS 251.83 | MH861580 | KJ398790 | MH873311 | KJ398649 | - | Egg of Heterodera avenae | Hou et al. (2023) |
| Hypocreomycetidae | Hypocreales | Clavicipitaceae | *Metapochonia variabilis* | LC5717 | KU746684 | KX855229 | KU746730 | KY883214 | KY883238 | Soil | Zhang et al. (2017) |
| Hypocreomycetidae | Hypocreales | Clavicipitaceae | *Metarhizium acridum* | ARSEF 7486 | HQ331458 | EU248845 | - | EU248897 | EU248925 | Orthoptera | Lin et al. (2025) |
| Hypocreomycetidae | Hypocreales | Clavicipitaceae | *Metarhizium anisopliae* | CBS 130.71 | MT078884 | MT078845 | MT078853 | MT078861 | MT078918 | Insect | Lin et al. (2025) |
| Hypocreomycetidae | Hypocreales | Clavicipitaceae | *Metarhizium baoshanense* | CCTCCM 2016589 | KY264172 | KY264169 | KY264174 | KY264180 | KY264183 | soil | Lin et al. (2025) |
| Hypocreomycetidae | Hypocreales | Clavicipitaceae | *Metarhizium bibionidarum* | KUNCC 10806 | PP256140 | PP328481 | PP256148 | PP294690 | PP314008 | March fly larvae Bibionidae, Diptera | Lin et al. (2025) |
| Hypocreomycetidae | Hypocreales | Clavicipitaceae | *Metarhizium dianzhongense* | KUNCC 10809 | PP256143 | PP328484 | PP256151 | PP294693 | PP314011 | White grubs, larvae of Scarabaeidae Coleoptera | Lin et al. (2025) |
| Hypocreomycetidae | Hypocreales | Clavicipitaceae | *Metarhizium flavoviride* | CBS 125.65 | MT078885 | MT078846 | MT078854 | MT078862 | MT078919 | Coleoptera | Lin et al. (2025) |
| Hypocreomycetidae | Hypocreales | Clavicipitaceae | *Metarhizium guizhouense* | CBS 258.90 | OP964701 | OQ440697 | OQ931838 | OQ957094 | OQ957109 | Lepidoptera | Hou et al. (2023) |
| Hypocreomycetidae | Hypocreales | Clavicipitaceae | *Metarhizium huainamdangense* | BCC 7672 | MN781901 | MN781711 | MN781856 | MN781758 | MN781806 | Leafhopper Hemiptera | Hou et al. (2023) |
| Hypocreomycetidae | Hypocreales | Clavicipitaceae | *Metarhizium rileyi* | CBS 806.71 | AY624205 | EF468787 | MH872111 | EF468893 | EF468937 | Lepidoptera | Lin et al. (2025) |
| Hypocreomycetidae | Hypocreales | Clavicipitaceae | *Metarhizium viridulum* | BCC 36261 | MT078878 | MN781680 | MN781827 | MN781737 | MN781781 | Cryptotympana facialis Hemiptera | Lin et al. (2025) |
| Hypocreomycetidae | Hypocreales | Clavicipitaceae | *Moelleriella alba* | BCC49409 | - | KX254423 | JQ269646 | JQ256906 | - | Whitefly nymphs | Wang et al. (2024e) |
| Hypocreomycetidae | Hypocreales | Clavicipitaceae | *Moelleriella chaiangmaiensis* | BCC60941 | - | MT672278 | MT659361 | MT672270 | - | Scale insect | Wang et al. (2024e) |
| Hypocreomycetidae | Hypocreales | Clavicipitaceae | *Moelleriella chumphonensis* | BCC47574 | - | KX254421 | JQ269647 | JQ256907 | - | Whitefly nymphs | Wang et al. (2024e) |
| Hypocreomycetidae | Hypocreales | Clavicipitaceae | *Moelleriella flava* | BCC60929 | - | KX254432 | KX298238 | MT672273 | - | Scale insects | Wang et al. (2024e) |
| Hypocreomycetidae | Hypocreales | Clavicipitaceae | *Moelleriella insperata* | ARSEF 2396 | - | DQ070029 | AY518374 | EU392713 | - | Scale insects and whiteflies | Wang et al. (2024e) |
| Hypocreomycetidae | Hypocreales | Clavicipitaceae | *Morakotia fusca* | BCC 79272 | - | KY794856 | KY794861 | KY794865 | - | Leaf sheath Poaceae | Mongkolsamrit et al. (2021b) |
| Hypocreomycetidae | Hypocreales | Clavicipitaceae | *Mycophilomyces periconiae* | CPC 27558 | KY173418 | KY173595 | KY173509 | - | - | Periconia sp. | Crous et al. (2013) |
| Hypocreomycetidae | Hypocreales | Clavicipitaceae | *Myriogenospora atramentosa* | AEG9632 | - | AY489628 | AY489733 | AY489665 | - | *Andropogon virginicus* | Lin et al. (2025) |
| Hypocreomycetidae | Hypocreales | Clavicipitaceae | *Neoaraneomyces araneicola* | DY10171 | MW730520 | MW753033 | MW730609 | - | MW753026 | Araneae: spider | Lin et al. (2025) |
| Hypocreomycetidae | Hypocreales | Clavicipitaceae | *Neoaraneomyces araneicola* | RCEF7543 | PV134480 | PV166481 | PV134539 | PV166544 | PV166579 | Araneae: spider | This study |
| Hypocreomycetidae | Hypocreales | Clavicipitaceae | *Nigelia aurantiaca* | BCC37621 | KY348783 | GU979955 | GU979946 | GU979964 | GU979970 | Larva of unidentified Lepidoptera | Kepler et al. (2012a) |
| Hypocreomycetidae | Hypocreales | Clavicipitaceae | *Nigelia martiale* | TTZ07071604 | JN049871 | JF416016 | JF415975 | JN049892 | JF415995 | Coleopteran | Kepler et al. (2012a) |
| Hypocreomycetidae | Hypocreales | Clavicipitaceae | *Orbiocrella petchii* | MFLU:220260 | OQ127339 | OQ186366 | OQ127374 | OQ186420 | OQ186396 | Scale insects attached to bamboo leaves | Dong et al. (2022b) |
| Hypocreomycetidae | Hypocreales | Clavicipitaceae | *Orbiocrella zlotorzyckae* | BRIP 72613a | OR527522 | OR514847 | OR527532 | - | OR514855 | Insect | Tan and Shiva (2023c) |
| Hypocreomycetidae | Hypocreales | Clavicipitaceae | *Papiliomyces albastromata* | YHH 2307002 | OR770519 | PP479838 | OR770504 | PP203269 | PP479841 | Larvae of Hepialidae in soil | Chen et al. (2025a) |
| Hypocreomycetidae | Hypocreales | Clavicipitaceae | *Papiliomyces longiclavatus* | YC20061403 | MZ702080 | MZ955880 | MZ702101 | MZ955876 | MZ955872 | Larvae of a bat moth Lepidoptera, Hepialidae | Chen et al. (2025a) |
| Hypocreomycetidae | Hypocreales | Clavicipitaceae | *Papiliomyces puniceum* | BUM1214 | OM955150 | OM988198 | OM951250 | OM988195 | OM988190 | Larva of Hepialidae | Chen et al. (2025a) |
| Hypocreomycetidae | Hypocreales | Clavicipitaceae | *Papiliomyces shibinense* | GZUHSB13050311 | KR153585 | KR153589 | - | KR153590 | - | Lepidopteran pupa | Zhang et al. (2023a) |
| Hypocreomycetidae | Hypocreales | Clavicipitaceae | *Parametarhizium changbaiense* | SGSF125 | MN589741 | MN908589 | MN589994 | MN917168 | MT921829 | Forest litters | Gao et al. (2021) |
| Hypocreomycetidae | Hypocreales | Clavicipitaceae | *Parametarhizium hingganense* | SGSF355 | MN055703 | MN065770 | MN061635 | MN917170 | MT939494 | Forest litters | Gao et al. (2021) |
| Hypocreomycetidae | Hypocreales | Clavicipitaceae | *Paraneoaraneomyces sinensis* | ZY 22.008 | OQ709256 | OQ719629 | OQ709262 | - | OQ719623 | Green belt soil | Zhang et al. (2023b) |
| Hypocreomycetidae | Hypocreales | Clavicipitaceae | *Pochonia boninensis* | JCM 18597 | AB709858 | AB758463 | AB709831 | AB758666 | AB758693 | Soil | Chen et al. (2025a) |
| Hypocreomycetidae | Hypocreales | Clavicipitaceae | *Pochonia cordycipiticonsociata* | CGMCC:317365 | KM263569 | KM263584 | KM263573 | KM263576 | KM263579 | *Hepialus armoricanus* | Huang et al. (2015) |
| Hypocreomycetidae | Hypocreales | Clavicipitaceae | *Pochonia sinensis* | ZY22.010 | OQ709258 | OQ719630 | OQ709264 | - | OQ719625 | Soil | Zhang et al. (2023b) |
| Hypocreomycetidae | Hypocreales | Clavicipitaceae | *Purpureomyces khaoyaiensis* | BCC1376 | KX983460 | KX983457 | KX983462 | - | KX983465 | Lepidoptera larva | Mongkolsamrit et al. (2020a) |
| Hypocreomycetidae | Hypocreales | Clavicipitaceae | *Purpureomyces maesotensis* | BCC89300 | MN781917 | MN781733 | MN781876 | MN781778 | - | Lepidoptera larva | Mongkolsamrit et al. (2020a) |
| Hypocreomycetidae | Hypocreales | Clavicipitaceae | *Purpureomyces pyriformis* | BCC85074 | MN781929 | MN781730 | MN781873 | MN781775 | MN781821 | Lepidoptera pupa | Chen et al. (2025a) |
| Hypocreomycetidae | Hypocreales | Clavicipitaceae | *Regiocrella camerunensis* | ARSEF 7682 | - | DQ118743 | DQ118735 | DQ127234 | - | Scale insects on living fern leaves | Mongkolsamrit et al. (2020a) |
| Hypocreomycetidae | Hypocreales | Clavicipitaceae | *Rousseaua marietteae* | BRIP 70297a | PP658052 | PP682355 | PP658053 | - | PP682354 | Araneae: spider | Tan and Shivas (2023d) |
| Hypocreomycetidae | Hypocreales | Clavicipitaceae | *Samuelsia chalalensis* | CUP067856 | - | EU392691 | EU392637 | EU392743 | - | Whiteflies on bamboo | Chaverri et al. (2008) |
| Hypocreomycetidae | Hypocreales | Clavicipitaceae | *Samuelsia geonomis* | CUP067857 | - | EU392692 | EU392638 | EU392744 | - | Scale insects or whiteflies on leaves of palm Geonomis sp | Chaverri et al. (2008) |
| Hypocreomycetidae | Hypocreales | Clavicipitaceae | *Samuelsia rufobrunnea* | CUP067858 | - | AY986944 | AY986918 | DQ000345 | - | Insects on leaves of palm Geonomis sp | Chaverri et al. (2008) |
| Hypocreomycetidae | Hypocreales | Clavicipitaceae | *Samuelsia sheikhii* | CUP067859 | - | EU392693 | EU392639 | EU392745 | - | Scale insects or whiteflies on leaves. | Chaverri et al. (2008) |
| Hypocreomycetidae | Hypocreales | Clavicipitaceae | *Shimizuomyces paradoxus* | EFCC 6279 | JN049847 | EF469071 | EF469084 | EF469100 | EF469117 | Smilacaceae | Hou et al. (2023) |
| Hypocreomycetidae | Hypocreales | Clavicipitaceae | *Subuliphorum camptosporum* | CBS:756.69 | OQ429878 | - | OQ430129 | - | OQ454278 | Air | Hou et al. (2023) |
| Hypocreomycetidae | Hypocreales | Clavicipitaceae | *Sungia yongmunensis* | EFCC 2131 | JN049856 | EF468770 | EF468833 | KJ398633 | KJ398730 | Lepidoptera pupa | Kepler et al. (2014) |
| Hypocreomycetidae | Hypocreales | Clavicipitaceae | *Ustilaginoidea dichromenae* | MRL IB9228 | - | JQ257025 | JQ257010 | JQ257013 | JQ257018 | Cyperaceae | Kepler et al. (2012b) |
| Hypocreomycetidae | Hypocreales | Clavicipitaceae | *Ustilaginoidea usambarensis* | TNSF87158 | LC474613 | LC474622 | - | LC474625 | LC474628 | Gramineaceae | Tanaka et al. (2020) |
| Hypocreomycetidae | Hypocreales | Clavicipitaceae | *Yosiokobayasia kusanagiensis* | TNSF 18494 | - | JF416014 | JF415972 | JN049890 | - | Lepidoptera pupa | Chen et al. (2025a) |
| Hypocreomycetidae | Hypocreales | Cocoonihabitaceae | *Cocoonihabitus sinensis* | HMAS254523 | KY924870 | - | KY924869 | - | - | Saturniidae | Zhuang and Zeng (2017) |
| Hypocreomycetidae | Hypocreales | Cordycipitaceae | *Akanthomyces ampullifer* | CCF 6648 | PP437869 | PP436442 | PP437868 | PP436443 | PP436444 | *Limonia nubeculosa* | Kubátová et al. (2024) |
| Hypocreomycetidae | Hypocreales | Cordycipitaceae | *Akanthomyces australiensis* | BRIP 72630a | NR_191288 | OR514840 | OR527524 | - | OR514848 | Insect | Kubátová et al. (2024) |
| Hypocreomycetidae | Hypocreales | Cordycipitaceae | *Akanthomyces coccidioperitheciatus* | NHJ 6709 | JN049865 | EU369025 | EU369042 | EU369067 | EU369086 | Araneae: spider | Pu et al. (2025) |
| Hypocreomycetidae | Hypocreales | Cordycipitaceae | *Akanthomyces laosensis* | YFCC 1910941 | OQ509523 | OQ506286 | OQ509510 | - | OQ511549 | Lepidoptera: Noctuidae | Kubátová et al. (2024) |
| Hypocreomycetidae | Hypocreales | Cordycipitaceae | *Akanthomyces noctuidarum* | BCC 36265 | MT356072 | MT477978 | MT356084 | MT477994 | MT477987 | Lepidoptera | Pu et al. (2025) |
| Hypocreomycetidae | Hypocreales | Cordycipitaceae | *Akanthomyces pseudonoctuidarum* | YFCC 1808943 | OQ509525 | OQ506288 | OQ509512 | OQ511537 | OQ511551 | Lepidoptera: Noctuidae | Kubátová et al. (2024) |
| Hypocreomycetidae | Hypocreales | Cordycipitaceae | *Akanthomyces pyralidarum* | BCC 28816 | MT356080 | MT477982 | MT356091 | MT478000 | MT478007 | Lepidoptera | Pu et al. (2025) |
| Hypocreomycetidae | Hypocreales | Cordycipitaceae | *Akanthomyces taiwanicus* | NTUPPMCC 20-060 | MT974202 | MW200213 | MT974356 | MW200221 | MW200230 | Agrius convolvuli Lepidoptera | Pu et al. (2025) |
| Hypocreomycetidae | Hypocreales | Cordycipitaceae | *Akanthomyces tortricidarum* | BCC 72638 | MT356076 | MT478004 | MT356088 | MT477997 | MT477992 | Lepidoptera | Kubátová et al. (2024) |
| Hypocreomycetidae | Hypocreales | Cordycipitaceae | *Akanthomyces tuberculatus* | BCC 16819 | - | MF416490 | MF416546 | MF416647 | MF416444 | Lepidoptera | Kepler et al. (2017) |
| Hypocreomycetidae | Hypocreales | Cordycipitaceae | *Akanthomyces xixiuensis* | HKAS 125851 | OP693461 | OP838888 | OP693481 | OP838890 | OP838892 | Lepidoptera | Kubátová et al. (2024) |
| Hypocreomycetidae | Hypocreales | Cordycipitaceae | *Arachnidicola anhuiensis* | RCEF7681 | PV134497 | PV166497 | PV134556 | PV166551 | PV166595 | Araneae: spider | This study |
| Hypocreomycetidae | Hypocreales | Cordycipitaceae | *Arachnidicola araneicola* | GY29011 | MK942431 | MK955950 | - | MK955944 | MK955947 | Araneae: spider | Pu et al. (2025) |
| Hypocreomycetidae | Hypocreales | Cordycipitaceae | *Arachnidicola araneogena* | GZUIF DX2 | MH978179 | MH978187 | - | MH978182 | MH978185 | Araneae: spider | Chen et al. (2018) |
| Hypocreomycetidae | Hypocreales | Cordycipitaceae | *Arachnidicola bashanensis* | CQ05621 | OQ300412 | OQ325024 | OQ300420 | - | OQ349684 | Araneae: spider | Pu et al. (2025) |
| Hypocreomycetidae | Hypocreales | Cordycipitaceae | *Arachnidicola beibeiensis* | CQ05921 | OQ300415 | OQ325028 | OQ300424 | - | OQ349688 | Araneae: spider | Pu et al. (2025) |
| Hypocreomycetidae | Hypocreales | Cordycipitaceae | *Arachnidicola kanyawimiae* | TBRC 7242 | MF140751 | MF140838 | MF140718 | MF140784 | MF140808 | Araneae: spider | Pu et al. (2025) |
| Hypocreomycetidae | Hypocreales | Cordycipitaceae | *Arachnidicola kunmingensis* | YFCC 1808940 | OQ509522 | OQ506285 | OQ509509 | OQ511534 | OQ511548 | Araneae: spider | Pu et al. (2025) |
| Hypocreomycetidae | Hypocreales | Cordycipitaceae | *Arachnidicola subaraneicola* | YFCC 2107937 | OQ509527 | OQ506290 | OQ509514 | OQ511539 | OQ511553 | Araneae: spider | Pu et al. (2025) |
| Hypocreomycetidae | Hypocreales | Cordycipitaceae | *Arachnidicola sulphurea* | TBRC 7248 | MF140758 | MF140843 | MF140722 | MF140787 | MF140812 | Araneae: spider | Pu et al. (2025) |
| Hypocreomycetidae | Hypocreales | Cordycipitaceae | *Arachnidicola thailandica* | TBRC 7245 | MF140754 | MF140839 | - | - | MF140809 | Araneae: spider | Pu et al. (2025) |
| Hypocreomycetidae | Hypocreales | Cordycipitaceae | *Arachnidicola tiankengensis* | KY11571 | ON502848 | ON525447 | ON502825 | - | ON525446 | Araneae: spider | Pu et al. (2025) |
| Hypocreomycetidae | Hypocreales | Cordycipitaceae | *Arachnidicola waltergamsii* | TBRC 7252 | MF140748 | MF140834 | MF140714 | MF140782 | MF140806 | Araneae: spider | Pu et al. (2025) |
| Hypocreomycetidae | Hypocreales | Cordycipitaceae | *Araneicillium aranearum* | CBS726.73a | AJ292464 | EF468781 | AF339537 | EF468887 | EF468934 | Araneae: spider | Zare et al. (2000) |
| Hypocreomycetidae | Hypocreales | Cordycipitaceae | *Araneicillium araneicola* | BTCC F35 | AB378506 | - | - | - | - | Araneae: spider | Tan and Shivas (2023c) |
| Hypocreomycetidae | Hypocreales | Cordycipitaceae | *Araneicillium spenceae* | BRIP 72646a | OR750698 | OR737798 | - | - | OR737787 | Araneae: spider | Tan and Shivas (2023c) |
| Hypocreomycetidae | Hypocreales | Cordycipitaceae | *Ascopolyporus albus* | BCC 48975 | OL331502 | OL322035 | OL322048 | OL322056 | OL322065 | Coccoidea | Thanakitpipattana et al. (2022) |
| Hypocreomycetidae | Hypocreales | Cordycipitaceae | *Ascopolyporus galloides* | BCC 48704 | OL331509 | OL322031 | OL322044 | OL322055 | OL322062 | Coccoidea | Thanakitpipattana et al. (2022) |
| Hypocreomycetidae | Hypocreales | Cordycipitaceae | *Ascopolyporus griseoperitheciatus* | BCC25788 | OL331508 | OL322038 | OL322051 | OL322058 | OL322068 | Coccoidea | Thanakitpipattana et al. (2022) |
| Hypocreomycetidae | Hypocreales | Cordycipitaceae | *Ascopolyporus khaoyaiensis* | BCC 43741 | OL331513 | OL322040 | OL322041 | - | OL322070 | Coccoidea | Thanakitpipattana et al. (2022) |
| Hypocreomycetidae | Hypocreales | Cordycipitaceae | *Ascopolyporus polychrous* | PC546 | - | DQ118745 | AY886546 | DQ127236 | - | Coccoidea | Thanakitpipattana et al. (2022) |
| Hypocreomycetidae | Hypocreales | Cordycipitaceae | *Ascopolyporus purpuratus* | BCC88430 | OL331506 | OL322032 | OL322045 | OL322059 | OL322063 | Coccoidea | Thanakitpipattana et al. (2022) |
| Hypocreomycetidae | Hypocreales | Cordycipitaceae | *Ascopolyporus tibetensis* | HKAS 127121 | NR_189925 | OQ716552 | OQ702346 | - | - | Bamboo | Yu et al. (2023) |
| Hypocreomycetidae | Hypocreales | Cordycipitaceae | *Ascopolyporus villosus* | ARSEF 6355 | AY886544 | DQ118750 | - | DQ127241 | - | Coccoidea | Thanakitpipattana et al. (2022) |
| Hypocreomycetidae | Hypocreales | Cordycipitaceae | *Beauveri acridophila* | HUA 179219 | MG516585 | JQ958613 | JQ895541 | JX003857 | JX003841 | Acrididae: Ommatolampis sp. | Wang et al. (2022a) |
| Hypocreomycetidae | Hypocreales | Cordycipitaceae | *Beauveri araneola* | GZAC 150317 | KT961700 | KT961699 | - | KT961701 | - | Araneae: Spider | Wang et al. (2022a) |
| Hypocreomycetidae | Hypocreales | Cordycipitaceae | *Beauveri bassiana* | ARSEF 1564 | HQ880761 | HQ880974 | - | HQ880833 | HQ880905 | *Hyphantria cunea* | Wang et al. (2022a) |
| Hypocreomycetidae | Hypocreales | Cordycipitaceae | *Beauveri brongniartii* | ARSEF 617 | HQ880782 | HQ880991 | - | HQ880854 | HQ880926 | Coleoptera: Scarabaeidae | Wang et al. (2022a) |
| Hypocreomycetidae | Hypocreales | Cordycipitaceae | *Beauveria amorpha* | ARSEF 2641 | HQ880808 | - | - | HQ880880 | HQ880952 | Solenopsis sp. | Wang et al. (2022a) |
| Hypocreomycetidae | Hypocreales | Cordycipitaceae | *Beauveria araneola* | GZZY20241001-01 | PV134588 | PV166538 | PV134588 | PV166568 | PV166619 | Araneae: spider | This study |
| Hypocreomycetidae | Hypocreales | Cordycipitaceae | *Beauveria asiatica* | YFCC 5600 | - | MN576996 | MN576826 | MN576886 | MN576940 | Coleoptera: Cerambycidae | Wang et al. (2022a) |
| Hypocreomycetidae | Hypocreales | Cordycipitaceae | *Beauveria baoshanensis* | CCTCC AF20180117 | MG642827 | MG642897 | MG642840 | MG642854 | MG642867 | Coleoptera: Chrysomelidae | Wang et al. (2022a) |
| Hypocreomycetidae | Hypocreales | Cordycipitaceae | *Beauveria bassiana* | FJS20240723-47 | PV134517 | PV166521 | PV134579 | PV166564 | PV166610 | Araneae: spider | This study |
| Hypocreomycetidae | Hypocreales | Cordycipitaceae | *Beauveria caledonica* | ARSEF 2567 | HQ880817 | EF469057 | AF339520 | HQ880889 | HQ880961 | Soil | Wang et al. (2022a) |
| Hypocreomycetidae | Hypocreales | Cordycipitaceae | *Beauveria diapheromeriphila* | MCA 1557 | JQ958608 | JQ958612 | MF416529 | JX003851 | - | Diapheromeridae | Wang et al. (2022a) |
| Hypocreomycetidae | Hypocreales | Cordycipitaceae | *Beauveria gryllotalpidicola* | BCC81472 | MK632053 | MK632068 | MK632099 | MK632178 | MK632151 | Orthoptera: Gryllotalpidae | Thanakitpipattana et al. (2020) |
| Hypocreomycetidae | Hypocreales | Cordycipitaceae | *Beauveria hoplocheli* | MNHN-RF-06107 | KC339691 | KC339702 | - | KM453954 | KM453963 | Coleoptera: Melolonthidae | Wang et al. (2022a) |
| Hypocreomycetidae | Hypocreales | Cordycipitaceae | *Beauveria kipukae* | ARSEF 7032 | HQ880803 | HQ881005 | - | HQ880875 | HQ880947 | Homoptera: Delphacidae | Wang et al. (2022a) |
| Hypocreomycetidae | Hypocreales | Cordycipitaceae | *Beauveria lii* | RCEF5500 | JN689372 | JN689371 | - | JN689374 | JN689370 | Coleoptera: Coccinellidae | Wang et al. (2022a) |
| Hypocreomycetidae | Hypocreales | Cordycipitaceae | *Beauveria locustiphila* | TS881 | JQ958606 | JQ958619 | JQ895535 | JX003847 | JX003845 | Colpolopha sinuata | Wang et al. (2022a) |
| Hypocreomycetidae | Hypocreales | Cordycipitaceae | *Beauveria loeiensis* | BCC78420 | MK632042 | MK632070 | MK632101 | MK632180 | MK632154 | Orthoptera | Chuang et al. (2024) |
| Hypocreomycetidae | Hypocreales | Cordycipitaceae | *Beauveria majiangensis* | MFLU:22-0272 | OQ127342 | OQ186369 | OQ127377 | OQ186423 | - | Coleoptera | Wang et al. (2022a) |
| Hypocreomycetidae | Hypocreales | Cordycipitaceae | *Beauveria malawiensis* | BUB444 | MG642833 | MG642905 | MG642847 | MG642860 | MG642875 | Insect | Chuang et al. (2024) |
| Hypocreomycetidae | Hypocreales | Cordycipitaceae | *Beauveria polyrhachicola* | YFCC 859 | - | MW168236 | MW173995 | MW168202 | MW168219 | Hymenoptera:Formicidae | Wang et al. (2022a) |
| Hypocreomycetidae | Hypocreales | Cordycipitaceae | *Beauveria polyrhachicola* | RCEF7859 | PV134514 | PV166515 | PV134574 | PV166561 | PV166607 | Araneae: spider | This study |
| Hypocreomycetidae | Hypocreales | Cordycipitaceae | *Beauveria pseudobassiana* | YFCC 1806007 | - | MN523553 | MN523524 | MN523582 | MN523611 | Maladera sp. | Wang et al. (2022a) |
| Hypocreomycetidae | Hypocreales | Cordycipitaceae | *Beauveria pseudobassiana* | RCEF5413 | OL684598 | PV097801 | PV102576 | PV097801 | PV097804 | Araneae: spider | This study |
| Hypocreomycetidae | Hypocreales | Cordycipitaceae | *Beauveria rudraprayagi* | MTCC 8017 | JQ266173 | JQ990914 | - | JQ990892 | JQ990870 | Lepidoptera: Bombycidae | Wang et al. (2022a) |
| Hypocreomycetidae | Hypocreales | Cordycipitaceae | *Beauveria scarabaeidicola* | ARSEF 5689 | JN049827 | DQ522335 | AF339524 | DQ522380 | DQ522431 | Coleoptera: Scarabaeidae | Spatafora et al. (2007) |
| Hypocreomycetidae | Hypocreales | Cordycipitaceae | *Beauveria sinensis* | RCEF3903 | HQ270152 | HQ270151 | - | JX524283 | JX524284 | Lepidoptera: Geometridae | Wang et al. (2022a) |
| Hypocreomycetidae | Hypocreales | Cordycipitaceae | *Beauveria songmingensis* | YFCC 860 | - | MW168238 | MW173997 | MW168204 | MW168221 | Coleoptera: Scarabaeidae | Wang et al. (2022a) |
| Hypocreomycetidae | Hypocreales | Cordycipitaceae | *Beauveria sungii* | ARSEF 1685 | AY531990 | AY531899 | - | HQ880881 | HQ880953 | Coleoptera: Scarabaeidae | Chuang et al. (2024) |
| Hypocreomycetidae | Hypocreales | Cordycipitaceae | *Beauveria vermiconia* | ARSEF 2922 | HQ880822 | AY531920 | MH872616 | HQ880894 | HQ880966 | Soil | Wang et al. (2022a) |
| Hypocreomycetidae | Hypocreales | Cordycipitaceae | *Beauveria yunnanensis* | CCTCC AF2018010 | OP964682 | MW168240 | MW173999 | MW168206 | MW168223 | Lepidopteran pupa | Wang et al. (2022a) |
| Hypocreomycetidae | Hypocreales | Cordycipitaceae | *Bhushaniella rubra* | BCC 47541 | OQ892128 | OQ914428 | OQ892133 | OQ914431 | OQ914433 | Spider eggs in sac | Mongkolsamrit et al. (2023) |
| Hypocreomycetidae | Hypocreales | Cordycipitaceae | *Blackwellomyces aurantiacus* | BCC 85060 | MT000692 | MT017840 | MT003028 | MT017800 | MT017819 | Lepidoptera | Pu et al. (2025) |
| Hypocreomycetidae | Hypocreales | Cordycipitaceae | *Blackwellomyces calendulinus* | BCC 68502 | MT000695 | MT017843 | MT003031 | MT017803 | MT017822 | Coleoptera | Pu et al. (2025) |
| Hypocreomycetidae | Hypocreales | Cordycipitaceae | *Blackwellomyces cardinalis* | OSC 93609 | MH862928 | DQ522325 | AY184962 | DQ522370 | DQ522422 | Archolophinae | Pu et al. (2025) |
| Hypocreomycetidae | Hypocreales | Cordycipitaceae | *Blackwellomyces kaihuaensis* | HMAS 285455 | OQ981961 | OQ980401 | OQ981968 | OQ980409 | OQ980408 | Lepidoptera | Pu et al. (2025) |
| Hypocreomycetidae | Hypocreales | Cordycipitaceae | *Blackwellomyces lateris* | MFLU 18-0663 | MK086059 | MK069471 | MK086061 | MK084615 | MK079354 | Lepidoptera | Pu et al. (2025) |
| Hypocreomycetidae | Hypocreales | Cordycipitaceae | *Blackwellomyces minutus* | BCC 88269 | MT000696 | MT017844 | MT003032 | MT017804 | MT017823 | Coleoptera | Pu et al. (2025) |
| Hypocreomycetidae | Hypocreales | Cordycipitaceae | *Blackwellomyces pseudomilitaris* | TBRC 3662 | MT000700 | MT017848 | MT003036 | MT017808 | - | Lepidoptera | Mongkolsamrit et al. (2020b) |
| Hypocreomycetidae | Hypocreales | Cordycipitaceae | *Blackwellomyces roseostromatus* | BCC 91358 | MT000697 | MT017845 | MT003033 | MT017805 | MT017824 | Lepidoptera | Pu et al. (2025) |
| Hypocreomycetidae | Hypocreales | Cordycipitaceae | *Blackwellomyces taiwanensis* | NTUCC 20-071 | MT974225 | MW200242 | MT974409 | MW200246 | MW200250 | Tenebrionidae | Pu et al. (2025) |
| Hypocreomycetidae | Hypocreales | Cordycipitaceae | *Cordyceps amoene-rosea* | CBS 107.73 | MH860646 | MF416494 | MF416550 | MF416651 | MF416445 | Coleoptera | Dong et al. (2022b) |
| Hypocreomycetidae | Hypocreales | Cordycipitaceae | *Cordyceps araneae* | BCC 85066 | MT000703 | MT017851 | MT003038 | MT017811 | MT017829 | Araneae: spider | Dong et al. (2022b) |
| Hypocreomycetidae | Hypocreales | Cordycipitaceae | *Cordyceps bifusispora* | EFCC 8260 | AY245627 | EF468747 | EF468807 | EF468855 | EF468910 | Lepidopteran pupa | Dong et al. (2022b) |
| Hypocreomycetidae | Hypocreales | Cordycipitaceae | *Cordyceps blackwelliae* | TBRC 7256 | MF140736 | MF140822 | MF140702 | MF140771 | MF140795 | Lepidoptera pupae | Dong et al. (2022b) |
| Hypocreomycetidae | Hypocreales | Cordycipitaceae | *Cordyceps brevistroma* | BCC 78209 | - | MT017855 | MT003044 | MT017817 | MT017835 | Lepidoptera | Dong et al. (2022b) |
| Hypocreomycetidae | Hypocreales | Cordycipitaceae | *Cordyceps bullispora* | YFCC 8400 | - | OL473523 | OL468575 | OL739569 | OL473534 | Lepidopteran pupa | Dong et al. (2022b) |
| Hypocreomycetidae | Hypocreales | Cordycipitaceae | *Cordyceps caloceroides* | MCA 2249 | - | MF416470 | MF416525 | MF416632 | - | Araneae: spider | Dong et al. (2022b) |
| Hypocreomycetidae | Hypocreales | Cordycipitaceae | *Cordyceps cateniannulata* | HKAS:102451 | OQ127347 | OQ186373 | OQ127381 | - | OQ186401 | Insect cocoon | Wei et al. (2022) |
| Hypocreomycetidae | Hypocreales | Cordycipitaceae | *Cordyceps cateniobliqua* | HKAS:102458 | OQ127346 | OQ186372 | OQ127380 | OQ186426 | OQ186400 | Insect larvae | Wei et al. (2022) |
| Hypocreomycetidae | Hypocreales | Cordycipitaceae | *Cordyceps cf. ochraceostromata* | ARSEF 5691 | JN049849 | EF468759 | EF468819 | EF468867 | EF468921 | Lepidoptera | Dong et al. (2022b) |
| Hypocreomycetidae | Hypocreales | Cordycipitaceae | *Cordyceps chiangdaoensis* | YFCC 857 | - | MW168234 | MW173993 | MW168200 | MW168217 | Coleoptera | Dong et al. (2022b) |
| Hypocreomycetidae | Hypocreales | Cordycipitaceae | *Cordyceps cicadae* | HKAS:102460 | OQ127350 | OQ186376 | OQ127384 | OQ186428 | OQ186402 | Cicada nymph | Mongkolsamrit et al. (2020b) |
| Hypocreomycetidae | Hypocreales | Cordycipitaceae | *Cordyceps cocoonihabita* | YFCC 3415 | - | MN576949 | MN576779 | MN576839 | MN576895 | Lepidoptera | Chuang et al. (2024) |
| Hypocreomycetidae | Hypocreales | Cordycipitaceae | *Cordyceps coleopterorum* | CBS 110.73 | AY624177 | JQ425689 | JF415988 | JN049903 | JF416006 | Coleoptera | Dong et al. (2022b) |
| Hypocreomycetidae | Hypocreales | Cordycipitaceae | *Cordyceps farinosa* | CBS 111113 | MZ853944 | MF416499 | MF416554 | MF416656 | MF416450 | Lepidoptera pupa | Dong et al. (2022b) |
| Hypocreomycetidae | Hypocreales | Cordycipitaceae | *Cordyceps fumosorosea* | NTUPPMCC 18-112 | MT966054 | MW025847 | MT974267 | MW025895 | MW025929 | Insect | Dong et al. (2022b) |
| Hypocreomycetidae | Hypocreales | Cordycipitaceae | *Cordyceps fumosorosea* | RCEF7555 | PV134492 | PV166493 | PV134551 | PV166549 | PV166591 | Araneae: spider | This study |
| Hypocreomycetidae | Hypocreales | Cordycipitaceae | *Cordyceps hehuanensis* | NTUPPMCC 18-144 | MT966065 | MW025861 | MT974287 | MW025906 | MW025943 | lepidopteran | Chuang et al. (2024) |
| Hypocreomycetidae | Hypocreales | Cordycipitaceae | *Cordyceps inthanonensis* | BCC 56302 | MT000705 | MT017853 | MT003040 | MT017814 | MT017831 | Lepidoptera | Dong et al. (2025) |
| Hypocreomycetidae | Hypocreales | Cordycipitaceae | *Cordyceps jakajanicola* | BCC 79816 | - | MN338479 | MN275696 | MN338484 | MN338489 | Hemiptera cicada nymph | Dong et al. (2025) |
| Hypocreomycetidae | Hypocreales | Cordycipitaceae | *Cordyceps javanica* | CBS 134.22 | MH854719 | MF416504 | MF416558 | MF416661 | MF416455 | Coleoptera | Dong et al. (2025) |
| Hypocreomycetidae | Hypocreales | Cordycipitaceae | *Cordyceps koratensis* | NHJ 2662 | GQ250008 | GQ250032 | GQ249982 | ON470206 | ON470208 | Araneae: spider | Mongkolsamrit et al. (2022) |
| Hypocreomycetidae | Hypocreales | Cordycipitaceae | *Cordyceps kuiburiensis* | BCC 90322 | MN099707 | MK988032 | MK968816 | MK988030 | - | Araneae: spider | Dong et al. (2025) |
| Hypocreomycetidae | Hypocreales | Cordycipitaceae | *Cordyceps lepidopterorum* | TBRC 7263 | MF140765 | MF140819 | MF140699 | MF140768 | MF140792 | Lepidoptera | Dong et al. (2025) |
| Hypocreomycetidae | Hypocreales | Cordycipitaceae | *Cordyceps locastrae* | NTUPPMCC 17-042 | MT966044 | MW025837 | MT974256 | MW025883 | MW025917 | Locastra muscosalis Lepidoptera pupa | Chuang et al. (2024) |
| Hypocreomycetidae | Hypocreales | Cordycipitaceae | *Cordyceps malleiformis* | NTUPPMCC 18-143 | MT966063 | MW025856 | MT974282 | MW025902 | MW025938 | Lepidopteran pupa | Chuang et al. (2024) |
| Hypocreomycetidae | Hypocreales | Cordycipitaceae | *Cordyceps militaris* | NTUPPMCC 17-039 | MT966047 | MW025840 | MT974259 | MW025886 | MW025920 | Lepidopteran pupa | Chuang et al. (2024) |
| Hypocreomycetidae | Hypocreales | Cordycipitaceae | *Cordyceps neopruinosa* | BCC 91362 | MT000712 | MT017859 | MT003048 | MT017818 | MT017839 | Lepidoptera | Dong et al. (2022b) |
| Hypocreomycetidae | Hypocreales | Cordycipitaceae | *Cordyceps nidus* | HUA 186125 | - | KC610722 | KC610752 | - | KC610711 | Araneae: spider | Dong et al. (2022b) |
| Hypocreomycetidae | Hypocreales | Cordycipitaceae | *Cordyceps ninchukispora* | BCRC 31900 | MT965715 | MT987641 | MT974311 | MW025916 | MW025953 | Seed of Beilschmiedia erythrophloia | Chuang et al. (2024) |
| Hypocreomycetidae | Hypocreales | Cordycipitaceae | *Cordyceps nodulosa* | IMI 338014R | EF513012 | - | - | - | - | Pyralidae | Kouvelis et al. (2008) |
| Hypocreomycetidae | Hypocreales | Cordycipitaceae | *Cordyceps poluscapitis* | CS21040411 | OM905725 | OM955655 | OM905730 | OM955649 | OM955652 | Formicidae Hymenoptera | Peng et al. (2023) |
| Hypocreomycetidae | Hypocreales | Cordycipitaceae | *Cordyceps pruinosa* | ARSEF 5413 | JN049826 | DQ522351 | AY184968 | DQ522397 | DQ522451 | Lepidoptera | Spatafora et al. (2007) |
| Hypocreomycetidae | Hypocreales | Cordycipitaceae | *Cordyceps pseudorosea* | NTUPPMCC 17-041 | MT966045 | MW025838 | MT974257 | MW025884 | MW025918 | Erebid adult Lepidoptera | Chuang et al. (2024) |
| Hypocreomycetidae | Hypocreales | Cordycipitaceae | *Cordyceps qingchengensis* | HKAS:102444 | OQ127352 | OQ186378 | OQ127386 | OQ186429 | OQ186403 | Insect cocoon | Wei et al. (2022) |
| Hypocreomycetidae | Hypocreales | Cordycipitaceae | *Cordyceps siangyangensis* | NTUPPMCC 18-150 | MT966073 | MW025872 | MT974300 | MW025911 | MW025948 | Insect | Chuang et al. (2024) |
| Hypocreomycetidae | Hypocreales | Cordycipitaceae | *Cordyceps sp.* | CBS 102184 | JN049858 | EF468803 | AF339564 | EF468907 | EF468948 | Araneae: spider | Dong et al. (2022b) |
| Hypocreomycetidae | Hypocreales | Cordycipitaceae | *Cordyceps tenuipes* | ARSEF 5135 | AY624196 | JF416020 | JF415980 | JN049896 | JF416000 | Lepidoptera | Dong et al. (2022b) |
| Hypocreomycetidae | Hypocreales | Cordycipitaceae | *Cordyceps tenuipes* | RCEF7553 | PV134490 | PV166491 | PV134549 | PV166548 | PV166589 | Araneae: spider | This study |
| Hypocreomycetidae | Hypocreales | Cordycipitaceae | *Cordyceps yaoluopingensis* | CGMCC 23076 | ON311002 | ON314456 | ON311006 | ON314458 | ON314454 | Lepidoptera pupa | Chuang et al. (2024) |
| Hypocreomycetidae | Hypocreales | Cordycipitaceae | *Corniculantispora aranearum* | CBS 797.84 | - | KM283811 | KM283787 | KM283833 | KM283853 | Agaricus bisporus | Khonsanit et al. (2024) |
| Hypocreomycetidae | Hypocreales | Cordycipitaceae | *Corniculantispora dimorphum* | CBS 363.86 | MH873656 | EF468784 | AF339559 | EF468890 | - | Agaricus bisporus | Khonsanit et al. (2024) |
| Hypocreomycetidae | Hypocreales | Cordycipitaceae | *Corniculantispora margaretspencerae* | BRIP 72656a | OR527517 | OR514841 | NG_242150 | - | OR514849 | Insect | Tan and Shivas (2023c) |
| Hypocreomycetidae | Hypocreales | Cordycipitaceae | *Corniculantispora margaretspencerae* | RCEF7796 | PV134505 | PV166504 | PV134564 | PV166554 | - | Araneae: spider | This study |
| Hypocreomycetidae | Hypocreales | Cordycipitaceae | *Corniculantispora psalliotae* | CBS 367.86 | - | KM283823 | KM283800 | - | - | Puccinia graminis | Khonsanit et al. (2024) |
| Hypocreomycetidae | Hypocreales | Cordycipitaceae | *Corniculantispora psalliotae* | CBS 532.81 | MH861374 | EF469067 | AF339560 | EF469096 | EF469112 | Soil | Khonsanit et al. (2024) |
| Hypocreomycetidae | Hypocreales | Cordycipitaceae | *Corniculantispora saksenae* | IMI 179841 | AJ292432 | - | - | - | - | Soil | Zare et al. (2000) |
| Hypocreomycetidae | Hypocreales | Cordycipitaceae | *Corpulentispora magnispora* | CGMCC 3.19304 | MK329102 | MK336037 | MK329007 | - | MK335985 | Soil | Zhang et al. (2021) |
| Hypocreomycetidae | Hypocreales | Cordycipitaceae | *Engyodontium aranearum* | CBS 309.85 | AJ292391 | DQ522341 | KM283802 | KM283844 | KM283866 | Araneae: spider | Spatafora et al. (2007) |
| Hypocreomycetidae | Hypocreales | Cordycipitaceae | *Engyodontium huhutii* | GZUIFR-huhu | MN944445 | MT006068 | - | MT006058 | MT006063 | Araneae: spider | Zhou et al. (2020) |
| Hypocreomycetidae | Hypocreales | Cordycipitaceae | *Engyodontium parvisporum* | IHEM 22910 | LC092896 | LC425558 | LC092915 | - | - | Coccoidea: Aspidiotus | Tsang et al. (2016) |
| Hypocreomycetidae | Hypocreales | Cordycipitaceae | *Engyodontium rectidentatum* | IRAN-3690 C | OR327477 | OR352918 | - | - | - | Coccidae | Armand et al. (2024) |
| Hypocreomycetidae | Hypocreales | Cordycipitaceae | *Flavocillium acerosum* | CBS 418.81 | MH861361 | KM283810 | KM283786 | KM283832 | KM283852 | Crinipellis perniciosa | Wang et al. (2020) |
| Hypocreomycetidae | Hypocreales | Cordycipitaceae | *Flavocillium bifurcatum* | YFCC 6101 | MN576833 | MN576951 | MN576781 | MN576841 | MN576897 | Lepidoptera pupa | Wang et al. (2020) |
| Hypocreomycetidae | Hypocreales | Cordycipitaceae | *Flavocillium praecognitum* | MGC39 | MT247058 | MT267523 | MT247060 | - | MT267525 | Insect | Wang et al. (2020) |
| Hypocreomycetidae | Hypocreales | Cordycipitaceae | *Gamszarea coprophilum* | CGMCC3.18986 | MH177616 | MH184587 | MH177619 | MH177622 | MH177624 | Marmota monax | Su et al. (2019) |
| Hypocreomycetidae | Hypocreales | Cordycipitaceae | *Gamszarea humicola* | CGMCC 3.19303 | MK329092 | MK336027 | MK328997 | - | MK335979 | Soil | Zhang et al. (2021) |
| Hypocreomycetidae | Hypocreales | Cordycipitaceae | *Gamszarea indonesiaca* | BTCC-F36 | AB378516 | - | - | - | - | Araneae: spider | Sukarno et al. (2009) |
| Hypocreomycetidae | Hypocreales | Cordycipitaceae | *Gamszarea lunata* | CGMCC 3.19315 | MK329094 | MK336029 | MK328999 | - | MK335981 | Rock | Zhang et al. (2021) |
| Hypocreomycetidae | Hypocreales | Cordycipitaceae | *Gamszarea microspora* | LRMH C331 | OR264322 | OR291126 | OR263657 | OR269655 | OR291088 | Cave surface | Zhang et al. (2021) |
| Hypocreomycetidae | Hypocreales | Cordycipitaceae | *Gamszarea testudineum* | UBOCC-A-112180 | LT992874 | LT992868 | LT992876 | - | - | Chemical solution of nickel | Zhang et al. (2021) |
| Hypocreomycetidae | Hypocreales | Cordycipitaceae | *Gamszarea wallacei* | CBS 101237 | EF641891 | EF469073 | AY184967 | EF469102 | EF469119 | Lepidopteran larva | Zhang et al. (2021) |
| Hypocreomycetidae | Hypocreales | Cordycipitaceae | *Gamszarella antillana* | CBS 350.85 | MH861888 | DQ522350 | AF339536 | DQ522396 | DQ522450 | Dead carpophore of basidiomycete | Spatafora et al. (2007) |
| Hypocreomycetidae | Hypocreales | Cordycipitaceae | *Gamszarella araneicola* | RCEF7803 | PV134570 | PV166510 | PV134570 | PV166558 | PV166602 | Araneae: spider | This study |
| Hypocreomycetidae | Hypocreales | Cordycipitaceae | *Gamszarella buffelskloofina* | CBS 150062 | OR680769 | OR683715 | OR717025 | - | OR683726 | Insect | Crous et al. (2023) |
| Hypocreomycetidae | Hypocreales | Cordycipitaceae | *Gamszarella sotirae* | BRIP 72673a | PP707902 | PP712789 | PP707921 | - | PP712792 | Insect | Tan and Shivas (2024b) |
| Hypocreomycetidae | Hypocreales | Cordycipitaceae | *Gibellula alba* | GNJ20210711-02 | - | OM863559 | OM090756 | - | - | Araneae: spider | This study |
| Hypocreomycetidae | Hypocreales | Cordycipitaceae | *Gibellula aurea* | 26PACOTI | OK329885 | OK392622 | - | - | OK315663 | Araneae: spider | Evans et al. (2025) |
| Hypocreomycetidae | Hypocreales | Cordycipitaceae | *Gibellula brevistipitata* | BCC57817 | OK040729 | OK040697 | OK040706 | OK040715 | - | Araneae: spider | Evans et al. (2025) |
| Hypocreomycetidae | Hypocreales | Cordycipitaceae | *Gibellula cebrennini* | BCC53605 | MT477069 | MT503328 | MT477062 | MT503321 | MT503336 | Araneae: spider | Evans et al. (2025) |
| Hypocreomycetidae | Hypocreales | Cordycipitaceae | *Gibellula clavulifera var. alba* | ARSEF1915 | JN049837 | DQ522360 | DQ518777 | DQ522408 | DQ522467 | Araneae: spider | Spatafora et al. (2007) |
| Hypocreomycetidae | Hypocreales | Cordycipitaceae | *Gibellula flava* | GNJ20200814-46 | - | MW961413 | MW969673 | MW980146 | - | Araneae: spider | Evans et al. (2025) |
| Hypocreomycetidae | Hypocreales | Cordycipitaceae | *Gibellula flava* | LS20230802-66 | - | PV166528 | - | - | PV166612 | Araneae: spider | This study |
| Hypocreomycetidae | Hypocreales | Cordycipitaceae | *Gibellula fusiformispora* | BCC56802 | MT477070 | MT503329 | MT477063 | MT503322 | MT503337 | Araneae: spider | Evans et al. (2025) |
| Hypocreomycetidae | Hypocreales | Cordycipitaceae | *Gibellula gamsii* | BCC28797 | MH152531 | MH152562 | MH152541 | MH152549 | MH152557 | Araneae: spider | Evans et al. (2025) |
| Hypocreomycetidae | Hypocreales | Cordycipitaceae | *Gibellula leiopus* | BCC16025 | OK070780 | OK070782 | OK070781 | OK070783 | OK070784 | Araneae: spider | Evans et al. (2025) |
| Hypocreomycetidae | Hypocreales | Cordycipitaceae | *Gibellula longicaudata* | BCC40861 | OK040730 | OK040698 | NG_088295 | OK040716 | OK040724 | Araneae: spider | Evans et al. (2025) |
| Hypocreomycetidae | Hypocreales | Cordycipitaceae | *Gibellula longispora* | GNJ20210710-02 | - | OL981628 | OL854212 | - | OL981635 | Araneae: spider | Evans et al. (2025) |
| Hypocreomycetidae | Hypocreales | Cordycipitaceae | *Gibellula longispora* | NHJ-12014 | - | EU369017 | - | EU369055 | EU369075 | Araneae: spider | Evans et al. (2025) |
| Hypocreomycetidae | Hypocreales | Cordycipitaceae | *Gibellula nigelii* | NHJ 10808 | - | EU369018 | EU369035 | EU369056 | EU369076 | Araneae: spider | Evans et al. (2025) |
| Hypocreomycetidae | Hypocreales | Cordycipitaceae | *Gibellula parvula* | BCC49748 | OK040732 | OK040700 | OK040709 | OK040718 | OK040726 | Araneae: spider | Evans et al. (2025) |
| Hypocreomycetidae | Hypocreales | Cordycipitaceae | *Gibellula penicillioides* | RCEF7512 | PV134473 | PV166473 | PV134532 | PV166542 | PV166572 | Araneae: spider | This study |
| Hypocreomycetidae | Hypocreales | Cordycipitaceae | *Gibellula pigmentosinum* | BCC39707 | MH532875 | MH521894 | MH394674 | MH521801 | MH521856 | Araneae: spider | Kuephadungphan et al. (2020) |
| Hypocreomycetidae | Hypocreales | Cordycipitaceae | *Gibellula pilosa* | BCC45580 | OK040733 | OK040701 | OK040710 | OK040719 | - | Araneae: spider | Evans et al. (2025) |
| Hypocreomycetidae | Hypocreales | Cordycipitaceae | *Gibellula pulchra* | NHJ 10788 | - | EU369019 | EU369036 | EU369058 | EU369078 | Araneae: spider | Evans et al. (2025) |
| Hypocreomycetidae | Hypocreales | Cordycipitaceae | *Gibellula queenslandica* | BRIP 72767a | OR452099 | OR459912 | OR452103 | - | OR459907 | Araneae: spider | Tan and Shivas (2023a) |
| Hypocreomycetidae | Hypocreales | Cordycipitaceae | *Gibellula scorpioides* | BCC47976 | MT477078 | MT503335 | MT477066 | MT503325 | MT503339 | Araneae: spider | Evans et al. (2025) |
| Hypocreomycetidae | Hypocreales | Cordycipitaceae | *Gibellula solita* | BCC45574 | OK040736 | OK040703 | OK040712 | OK040721 | - | Araneae: spider | Evans et al. (2025) |
| Hypocreomycetidae | Hypocreales | Cordycipitaceae | *Gibellula* sp. | EPF081 | JX192723 | JX192818 | JX192754 | - | - | Araneae: spider | This study |
| Hypocreomycetidae | Hypocreales | Cordycipitaceae | *Gibellula* sp. | MT20211006-01 | OM842972 | OM863558 | OM090755 | - | - | Araneae: spider | This study |
| Hypocreomycetidae | Hypocreales | Cordycipitaceae | *Gibellula* sp. XYC-2025e | RCEF7879 | - | PV166609 | - | - | PV166609 | Araneae: spider | This study |
| Hypocreomycetidae | Hypocreales | Cordycipitaceae | *Gibellula* sp. XYC-2025f | LS20230802-73 | - | PV166532 | - | - | PV166616 | Araneae: spider | This study |
| Hypocreomycetidae | Hypocreales | Cordycipitaceae | *Gibellula trimorpha* | BCC36526 | OK040737 | OK040704 | - | OK040722 | OK040728 | Araneae: spider | Evans et al. (2025) |
| Hypocreomycetidae | Hypocreales | Cordycipitaceae | *Gibellula unica* | BCC45112 | OK040738 | OK040705 | OK040713 | OK040723 | MH521866 | Araneae: spider | Evans et al. (2025) |
| Hypocreomycetidae | Hypocreales | Cordycipitaceae | *Hevansia cf. novoguineensis* | NHJ4314 | - | EU369012 | - | EU369051 | EU369071 | Araneae: spider | Mongkolsamrit et al. (2024) |
| Hypocreomycetidae | Hypocreales | Cordycipitaceae | *Hevansia cf. websteri* | BCC36541 | MH532868 | MH521889 | MH394669 | MH521811 | MH521849 | Araneae: spider | Mongkolsamrit et al. (2022) |
| Hypocreomycetidae | Hypocreales | Cordycipitaceae | *Hevansia mainiae* | BRIP 62570a | - | OQ054473 | OQ025237 | - | OQ054472 | Araneae: spider | Voglmayr and Mombert (2023) |
| Hypocreomycetidae | Hypocreales | Cordycipitaceae | *Hevansia minuta* | BCC47519 | MZ684087 | MZ707811 | MZ684002 | MZ707826 | MZ707833 | Araneae: Meotipa sp. | Evans et al. (2025) |
| Hypocreomycetidae | Hypocreales | Cordycipitaceae | *Hevansia nelumboides* | BCC41864 | JN201871 | JN201867 | JN201873 | - | - | Araneae: spider | Evans et al. (2025) |
| Hypocreomycetidae | Hypocreales | Cordycipitaceae | *Hevansia novoguineensis* | BCC47881 | - | MH521886 | MH394650 | MH521807 | MH521845 | Araneae: spider | Evans et al. (2025) |
| Hypocreomycetidae | Hypocreales | Cordycipitaceae | *Hevansia novoguineensis* | CBS610.80 | MH532831 | MH521885 | MH394646 | - | MH521844 | Araneae: spider | Evans et al. (2025) |
| Hypocreomycetidae | Hypocreales | Cordycipitaceae | *Hevansia psuedonelumboides* | GNJ20210710-05 | - | ON326400 | OM976882 | - | - | Araneae: spider | This study |
| Hypocreomycetidae | Hypocreales | Cordycipitaceae | *Hevansia psuedonelumboides* | RCEF7275 | - | PV166469 | PV134528 | PV166540 | PV166571 | Araneae: spider | This study |
| Hypocreomycetidae | Hypocreales | Cordycipitaceae | *Jenniferia griseocinerea* | BCC42063 | MZ684092 | MZ707816 | MZ684007 | MZ707829 | MZ707838 | Araneae: Diaea cf. dorsata | Evans et al. (2025) |
| Hypocreomycetidae | Hypocreales | Cordycipitaceae | *Jenniferia cinerea* | BCC47914 | - | MH521888 | MH394652 | MH521821 | MH521851 | Araneae: spider | Evans et al. (2025) |
| Hypocreomycetidae | Hypocreales | Cordycipitaceae | *Jenniferia thomisidarum* | BCC37882 | MZ684100 | MZ707824 | MZ684011 | MZ707831 | MZ707844 | Araneae: Diaea cf. dorsata | Evans et al. (2025) |
| Hypocreomycetidae | Hypocreales | Cordycipitaceae | *Kanoksria zaquensis* | HMAS 246915 | MT789699 | MT797812 | MT789697 | MT797810 | - | Stroma and sclerotium of Ophiocordyceps sinensis | Khonsanit et al. (2024) |
| Hypocreomycetidae | Hypocreales | Cordycipitaceae | *Lecanicillium araneogenum* | GZU1031Lea | KX845703 | KX845697 | - | KX845699 | KX845701 | Araneae: spider | Pu et al. (2025) |
| Hypocreomycetidae | Hypocreales | Cordycipitaceae | *Lecanicillium araneosum* | KY11341 | ON502826 | ON525443 | ON502832 | - | ON525442 | Araneae: spider | Pu et al. (2025) |
| Hypocreomycetidae | Hypocreales | Cordycipitaceae | *Lecanicillium attenuatum* | CBS 170.76 | MH860970 | OP762607 | OP752153 | OP762611 | OP762615 | *Carpocapsa pomonella* | Pu et al. (2025) |
| Hypocreomycetidae | Hypocreales | Cordycipitaceae | *Lecanicillium fusisporum* | CBS164.70 | AJ292428 | KM283817 | KM283793 | KM283836 | KM283858 | *Coltricia perennis* | Pu et al. (2025) |
| Hypocreomycetidae | Hypocreales | Cordycipitaceae | *Lecanicillium lecanii* | CBS102067 | MH862778 | KM283818 | KM283795 | KM283838 | KM283860 | *Coccus viridis* | Pu et al. (2025) |
| Hypocreomycetidae | Hypocreales | Cordycipitaceae | *Lecanicillium lecanii* | RCEF6920 | PV102573 | - | PV102575 | PV097800 | - | Araneae: spider | This study |
| Hypocreomycetidae | Hypocreales | Cordycipitaceae | *Lecanicillium lepidopterorum* | GZAC SD05151 | MT705973 | - | - | - | MT727044 | Lepidoptera pupa | Pu et al. (2025) |
| Hypocreomycetidae | Hypocreales | Cordycipitaceae | *Lecanicillium longisporum* | CBS 126.27 | AJ292385 | KM283820 | KM283797 | KR064300 | KM283862 | Coccoidea: Icerya purchasi | Pu et al. (2025) |
| Hypocreomycetidae | Hypocreales | Cordycipitaceae | *Lecanicillium muscarium* | CBS143.62 | MH858126 | KM283821 | KM283798 | KM283841 | KM283863 | Aleyrodoidea: rialeurodes vaporariorum | Pu et al. (2025) |
| Hypocreomycetidae | Hypocreales | Cordycipitaceae | *Lecanicillium neocoleopterorum* | GY11241 | MN093296 | MN097813 | - | MN097816 | MN097812 | Ladybug | Pu et al. (2025) |
| Hypocreomycetidae | Hypocreales | Cordycipitaceae | *Lecanicillium pissodis* | CBS118231 | - | KM283822 | KM283799 | KM283842 | KM283864 | Curculionidae: Pissodes strobi | Pu et al. (2025) |
| Hypocreomycetidae | Hypocreales | Cordycipitaceae | *Lecanicillium* sp. | YFCC 945 | OQ509531 | OQ506294 | - | OQ511543 | OQ511557 | Soil | Wang et al. (2024c) |
| Hypocreomycetidae | Hypocreales | Cordycipitaceae | *Lecanicillium uredinophilum* | KUN 101469 | MG948306 | MG948316 | MG948308 | MG948312 | MG948314 | Insect | Wang et al. (2024c) |
| Hypocreomycetidae | Hypocreales | Cordycipitaceae | *Leptobacillium cavernicola* | LRMH C299 | OM622527 | OM654337 | OM628786 | OM677786 | OM654326 | Surface sampling in the Pair-non-Pair cave | Dong et al. (2025) |
| Hypocreomycetidae | Hypocreales | Cordycipitaceae | *Leptobacillium chinense* | CGMCC 3.14970 | JQ410324 | - | JQ410322 | - | - | Wood submerged in fresh water | Dong et al. (2025) |
| Hypocreomycetidae | Hypocreales | Cordycipitaceae | *Leptobacillium coffeanum* | CDA734 | MF066034 | - | MF066032 | - | - | *Coffea arabica* | Gomes et al. (2018) |
| Hypocreomycetidae | Hypocreales | Cordycipitaceae | *Leptobacillium filiforme* | URM 7918 | MH979338 | - | MH979399 | - | - | *Citrullus lanatus* | Dong et al. (2025) |
| Hypocreomycetidae | Hypocreales | Cordycipitaceae | *Leptobacillium latisporum* | TBRC 16288 | OP856540 | - | OP856529 | - | - | Soil | Dong et al. (2025) |
| Hypocreomycetidae | Hypocreales | Cordycipitaceae | *Leptobacillium leptobactrum* | JCM 39056 | LC496868 | LC496918 | LC496888 | - | - | Soil | Kondo et al. (2020) |
| Hypocreomycetidae | Hypocreales | Cordycipitaceae | *Leptobacillium marksiae* | BRIP 70307a | PQ061114 | PQ067352 | PQ047739 | - | PQ067351 | Insecta | [Tan and Shivas (2024c)](https://www.mycobank.org/details/19/20087340) |
| Hypocreomycetidae | Hypocreales | Cordycipitaceae | *Leptobacillium muralicola* | CGMCC3.19014 | MH379983 | - | MH379997 | - | - | White biofilm of mural | Sun et al. (2019) |
| Hypocreomycetidae | Hypocreales | Cordycipitaceae | *Leptobacillium xianyushanense* | RCEF7511 | PV134472 | PV166472 | PV134531 | - | - | Araneae: spider | This study |
| Hypocreomycetidae | Hypocreales | Cordycipitaceae | *Leptobacillium xianyushanense* | RCEF6795 | PV102572 | PV097803 | PV102574 | PV097803 | PV097803 | Araneae: spider | This study |
| Hypocreomycetidae | Hypocreales | Cordycipitaceae | *Liangia guizhouensis* | RCEF7795 | PV134504 | PV166503 | PV134563 | PV166553 | PV166598 | Araneae: spider | This study |
| Hypocreomycetidae | Hypocreales | Cordycipitaceae | *Liangia sinensis* | YFCC 3103 | MN576831 | MN576952 | MN576782 | MN576842 | MN576898 | *Beauveria yunnanensis* | Wang et al. (2020) |
| Hypocreomycetidae | Hypocreales | Cordycipitaceae | *Matutinistella brasiliensis* | COAD 3686 | PP836714 | PP855559 | PP836707 | PP855576 | PP855536 | *Pseudocercospora fijiensis* | Custódio and Pereira (2025) |
| Hypocreomycetidae | Hypocreales | Cordycipitaceae | *Microhilum oncoperae* | AFSEF 4358 | - | EF468785 | AF339532 | EF468891 | EF468936 | Oncopera intricate Lepidoptera | Sung et al. (2007) |
| Hypocreomycetidae | Hypocreales | Cordycipitaceae | *Neobaryopsis andensis* | KRAM A.F.25967-2 | MT153957 | - | MT153986 | - | - | *Lobariella pallida* | Flakus et al. (2019) |
| Hypocreomycetidae | Hypocreales | Cordycipitaceae | *Neohyperdermium piperis* | CBS 116719 | - | DQ118749 | AY466442 | DQ127240 | EU369083 | Scale insect Coccoidea, Hemiptera | Johnson et al. (2009) |
| Hypocreomycetidae | Hypocreales | Cordycipitaceae | *Neohyperdermium pulvinatum* | P.C. 602 | - | DQ118746 | DQ118738 | DQ127237 | - | Asteraceae | Chaverri et al. (2005) |
| Hypocreomycetidae | Hypocreales | Cordycipitaceae | *Neotorrubiella chinghridicola* | BCC80733 | MK632039 | MK632072 | MK632097 | MK632176 | MK632149 | Gryllidae | Thanakitpipattana et al. (2020) |
| Hypocreomycetidae | Hypocreales | Cordycipitaceae | *Niveomyces albus* | BCC83025 | ON103032 | ON125015 | ON103157 | ON286876 | ON125027 | *Ophiocordyceps dipterigena* | Pu et al. (2025) |
| Hypocreomycetidae | Hypocreales | Cordycipitaceae | *Niveomyces coronatus* | NY04434800 | - | ON513397 | ON493606 | ON513399 | ON513400 | *Ophiocordyceps camponoti-floridani* | Pu et al. (2025) |
| Hypocreomycetidae | Hypocreales | Cordycipitaceae | *Niveomyces formicidarum* | BCC83026 | ON103036 | ON125019 | ON103161 | ON286879 | ON125030 | *Ophiocordyceps polyrhachis-furcata* | Pu et al. (2025) |
| Hypocreomycetidae | Hypocreales | Cordycipitaceae | *Niveomyces hirsutellae* | BCC36631 | ON103039 | ON125022 | ON103164 | ON286882 | ON125033 | *Hirsutella versicolor* | Pu et al. (2025) |
| Hypocreomycetidae | Hypocreales | Cordycipitaceae | *Niveomyces multisynnematus* | BCC90308 | ON103038 | ON125021 | ON103163 | ON286881 | ON125032 | *Ophiocordyceps flavida* | Pu et al. (2025) |
| Hypocreomycetidae | Hypocreales | Cordycipitaceae | *Parengyodontium album* | LRMH C343 | OL913889 | OM201771 | OL981482 | OL904981 | OL904999 | Airborne | Leplat et al. (2022) |
| Hypocreomycetidae | Hypocreales | Cordycipitaceae | *Pleurodesmospora acaricola* | BCRC FU31537 | MZ435417 | LC629776 | MZ435415 | - | - | Arachnida: Acari sp. | Yeh et al. (2021) |
| Hypocreomycetidae | Hypocreales | Cordycipitaceae | *Pleurodesmospora coccorum* | R. Kirschner 4968 | MZ435418 | LC629777 | MZ435416 | - | - | *Aleurocanthus camelliae* | Yeh et al. (2021) |
| Hypocreomycetidae | Hypocreales | Cordycipitaceae | *Pleurodesmospora entomophila* | BRIP 72652a | OR527518 | OR514842 | OR527526 | - | OR514850 | Insect | [Tan and Shivas (2023b)](https://www.mycobank.org/details/19/10112498) |
| Hypocreomycetidae | Hypocreales | Cordycipitaceae | *Pleurodesmospora lepidopterorum* | DY10501 | MW826577 | MW834317 | - | MW834315 | MW834316 | Lepidoptera pupa | Chen et al. (2021) |
| Hypocreomycetidae | Hypocreales | Cordycipitaceae | *Polystromomyces araneae* | BCC 93301 | MZ684101 | MZ707825 | MZ684016 | MZ707832 | MZ707845 | Araneae: spider | Pu et al. (2025) |
| Hypocreomycetidae | Hypocreales | Cordycipitaceae | *Pseudogibellula formicarum* | CBS 433.73 | MH860731 | MT533481 | MH872442 | MT533475 | - | *Ricania mediana* | Mongkolsamrit et al. (2021a) |
| Hypocreomycetidae | Hypocreales | Cordycipitaceae | *Pseudolecanicillium caatingaense* | URM8442 | ON862934 | OP290525 | ON862926 | - | OP290513 | Cave air | Alves et al. (2022) |
| Hypocreomycetidae | Hypocreales | Cordycipitaceae | *Pseudoniveomyces arachnophilus* | NHJ2465 | MH532899 | MH521916 | - | ON470205 | ON470207 | Araneae: spider | Kobmoo et al. (2023) |
| Hypocreomycetidae | Hypocreales | Cordycipitaceae | *Pseudoniveomyces arachnovorum* | BCC95818 | OR098526 | OR133172 | - | OR133173 | OR133174 | Arachnida of spider eggs | Kobmoo et al. (2023) |
| Hypocreomycetidae | Hypocreales | Cordycipitaceae | *Pseudoniveomyces blattae* | BCC53568 | ON103043 | ON125025 | ON103168 | ON286886 | ON125037 | Blattodea | Pu et al. (2025) |
| Hypocreomycetidae | Hypocreales | Cordycipitaceae | *Samsoniella alboaurantium* | CBS 240.32 | AY624178 | JF416019 | JF415979 | JN049895 | JF415999 | Lepidoptera pupa | Chen et al. (2023) |
| Hypocreomycetidae | Hypocreales | Cordycipitaceae | *Samsoniella alpina* | YFCC 5836 | - | MN576981 | MN576811 | MN576871 | MN576925 | Lepidoptera: Hepialus sp. | Chen et al. (2023) |
| Hypocreomycetidae | Hypocreales | Cordycipitaceae | *Samsoniella anhuiensis* | RCEF2590 | - | OR966516 | OR978316 | OR989964 | - | Lepidoptera pupa | Pu et al. (2025) |
| Hypocreomycetidae | Hypocreales | Cordycipitaceae | *Samsoniella anhuiensis* | RCEF2830 | OM268837 | OM483864 | OM268837 | OM751889 | PV097805 | Araneae: spider | Pu et al. (2025) |
| Hypocreomycetidae | Hypocreales | Cordycipitaceae | *Samsoniella antleroides* | YFCC 6016 | - | MN576973 | MN576803 | MN576863 | MN576917 | Lepidoptera: Noctuidae | Pu et al. (2025) |
| Hypocreomycetidae | Hypocreales | Cordycipitaceae | *Samsoniella aranea* | RCEF2868 | OM268839 | OM483866 | OM268850 | OM751883 | OM802501 | Araneae: spider | Pu et al. (2025) |
| Hypocreomycetidae | Hypocreales | Cordycipitaceae | *Samsoniella aranea* | RCEF2831 | OM268838 | OM483865 | OM268838 | OM751882 | OM802500 | Araneae: spider | Pu et al. (2025) |
| Hypocreomycetidae | Hypocreales | Cordycipitaceae | *Samsoniella asiatica* | YFCC 869 | OQ476473 | OQ506153 | - | OQ506195 | OQ506187 | Lepidoptera pupa | Wang et al. (2023b) |
| Hypocreomycetidae | Hypocreales | Cordycipitaceae | *Samsoniella cardinalis* | YFCC 5830 | OQ476478 | MN576958 | MN576788 | MN576848 | MN576902 | Limacodidae pupa | Chen et al. (2023) |
| Hypocreomycetidae | Hypocreales | Cordycipitaceae | *Samsoniella coccinellidicola* | YFCC8772 | ON621670 | ON676514 | ON621670 | ON676502 | ON568685 | Coccinellidae sp. | Chen et al. (2023) |
| Hypocreomycetidae | Hypocreales | Cordycipitaceae | *Samsoniella cristata* | YFCC 7004 | - | MN576963 | MN576793 | MN576853 | MN576907 | Saturniidae pupa | Pu et al. (2025) |
| Hypocreomycetidae | Hypocreales | Cordycipitaceae | *Samsoniella duyunensis* | DY09161 | OQ379241 | OQ398145 | OQ363112 | OR296698 | OQ397660 | Formicidae | Chen et al. (2023) |
| Hypocreomycetidae | Hypocreales | Cordycipitaceae | *Samsoniella erucae* | KY11121 | ON502828 | ON525425 | ON502835 | - | ON525424 | *Caterpillar* | Pu et al. (2025) |
| Hypocreomycetidae | Hypocreales | Cordycipitaceae | *Samsoniella erucae* | RCEF2595 | OM751888 | OM483863 | OM751888 | OM751888 | - | Araneae: spider | This study |
| Hypocreomycetidae | Hypocreales | Cordycipitaceae | *Samsoniella farinospora* | YFCC8774 | - | ON676516 | ON621672 | ON676504 | ON568687 | Araneae: spider | Pu et al. (2025) |
| Hypocreomycetidae | Hypocreales | Cordycipitaceae | *Samsoniella formicae* | KY11041 | ON502852 | ON525421 | - | - | ON525420 | Formicidae | Chen et al. (2023) |
| Hypocreomycetidae | Hypocreales | Cordycipitaceae | *Samsoniella fusiformispora* | RCEF2588 | - | OR966515 | OR978315 | - | - | Lepidoptera pupa | This study |
| Hypocreomycetidae | Hypocreales | Cordycipitaceae | *Samsoniella fusiformispora* | RCEF5406 | OM268840 | OM483867 | OM268851 | OM751890 | - | Araneae: spider | Pu et al. (2025) |
| Hypocreomycetidae | Hypocreales | Cordycipitaceae | *Samsoniella guizhouensis* | KY11161 | ON502823 | ON525429 | ON502830 | - | ON525428 | Lepidoptera pupa | Chen et al. (2023) |
| Hypocreomycetidae | Hypocreales | Cordycipitaceae | *Samsoniella hepiali* | NTUPPMCC 18-159 | MT974209 | - | MT974362 | MW200228 | MW200237 | Lepidopteran larvae | Chuang et al. (2024) |
| Hypocreomycetidae | Hypocreales | Cordycipitaceae | *Samsoniella hymenopterorum* | A19522 | MN128081 | MN101591 | - | MN101589 | MN101590 | Beetle | Pu et al. (2025) |
| Hypocreomycetidae | Hypocreales | Cordycipitaceae | *Samsoniella inthanonensis* | TBRC 7915 | MF140761 | MF140849 | MF140725 | MF140790 | MF140815 | Lepidopteran larvae | Pu et al. (2025) |
| Hypocreomycetidae | Hypocreales | Cordycipitaceae | *Samsoniella kunmingensis* | YHH16002 | - | MN576972 | MN576802 | MN576862 | MN576916 | Lepidoptera pupa | Pu et al. (2025) |
| Hypocreomycetidae | Hypocreales | Cordycipitaceae | *Samsoniella lasiocampidarum* | NTUPPMCC 20-062 | MT974208 | MW200218 | MT974361 | MW200227 | MW200236 | Lasiocampid larva  Lepidoptera | Pu et al. (2025) |
| Hypocreomycetidae | Hypocreales | Cordycipitaceae | *Samsoniella lepidopterorum* | DL10071 | MN128076 | MN101594 | - | MN101592 | MN101593 | Lepidoptera pupa | Chuang et al. (2024) |
| Hypocreomycetidae | Hypocreales | Cordycipitaceae | *Samsoniella neopupicola* | KY11321 | ON502843 | ON525433 | ON502839 | - | ON525432 | Lepidoptera pupa | Pu et al. (2025) |
| Hypocreomycetidae | Hypocreales | Cordycipitaceae | *Samsoniella sapaensis* | YFCC873 | OQ476489 | OQ506152 | - | OQ506194 | OQ506186 | Lepidoptera pupa | Wang et al. (2023b) |
| Hypocreomycetidae | Hypocreales | Cordycipitaceae | *Samsoniella sinensis* | YFCC8766 | - | ON676523 | ON621679 | ON676511 | ON568694 | Lepidoptera pupa | Pu et al. (2025) |
| Hypocreomycetidae | Hypocreales | Cordycipitaceae | *Samsoniella tiankengensis* | KY11741 | ON502840 | ON525437 | ON502838 | - | ON525436 | Lepidoptera pupa | Pu et al. (2025) |
| Hypocreomycetidae | Hypocreales | Cordycipitaceae | *Samsoniella tortricidae* | YFCC 6131 | - | MN576976 | MN576806 | MN576866 | MN576920 | Tortricidae pupa | Pu et al. (2025) |
| Hypocreomycetidae | Hypocreales | Cordycipitaceae | *Samsoniella vallis* | DY07241 | OR263159 | OR282778 | OR263306 | OR282772 | OR282774 | Lepidoptera pupa | Pu et al. (2025) |
| Hypocreomycetidae | Hypocreales | Cordycipitaceae | *Samsoniella yuanzuiensis* | NTUPPMCC 20-065 | MT974207 | MW200217 | MT974360 | MW200226 | MW200235 | Insect pupa | Pu et al. (2025) |
| Hypocreomycetidae | Hypocreales | Cordycipitaceae | *Simplicillium album* | CGMCC 3.19635 | MK329133 | MK336068 | MK329038 | - | - | Soil | Chen et al. (2022) |
| Hypocreomycetidae | Hypocreales | Cordycipitaceae | *Simplicillium aogashimaense* | JCM 18167 | AB604002 | LC496904 | LC496874 | - | - | Soil | Chuang et al. (2024) |
| Hypocreomycetidae | Hypocreales | Cordycipitaceae | *Simplicillium araneae* | DY101811 | OM743774 | OM818465 | OM743792 | - | - | Araneae: spider | Chen et al. (2022) |
| Hypocreomycetidae | Hypocreales | Cordycipitaceae | *Simplicillium calcicola* | CGMCC 3.17943 | KU746706 | KX855252 | KU746752 | - | KY883257 | Rock | Chuang et al. (2024) |
| Hypocreomycetidae | Hypocreales | Cordycipitaceae | *Simplicillium cicadellidae* | GY11011 | MN006249 | MN022263 | - | MN022271 | - | Leafhopper | Chuang et al. (2024) |
| Hypocreomycetidae | Hypocreales | Cordycipitaceae | *Simplicillium coccinellidae* | DY101791 | MT453861 | MT471341 | MT453862 | - | - | Coccinellidae | Chen et al. (2022) |
| Hypocreomycetidae | Hypocreales | Cordycipitaceae | *Simplicillium coleopterorum* | SD05381 | OM743920 | OM818467 | OM743925 | - | - | Beetle | Chuang et al. (2024) |
| Hypocreomycetidae | Hypocreales | Cordycipitaceae | *Simplicillium cylindrosporum* | JCM 18169 | AB603989 | LC496906 | LC496876 | - | - | Soil | Chuang et al. (2024) |
| Hypocreomycetidae | Hypocreales | Cordycipitaceae | *Simplicillium formicae* | MFLUCC 18-1379 | MK766511 | MK926451 | MK766512 | MK882623 | - | Formicidae Hymenoptera | Chuang et al. (2024) |
| Hypocreomycetidae | Hypocreales | Cordycipitaceae | *Simplicillium guizhouense* | DY10052 | OM743241 | OM818454 | OM743252 | - | - | Ant | Chen et al. (2022) |
| Hypocreomycetidae | Hypocreales | Cordycipitaceae | *Simplicillium humicola* | CGMCC 3.19573 | MK329136 | MK336071 | MK329041 | - | - | Soil | Dong et al. (2025) |
| Hypocreomycetidae | Hypocreales | Cordycipitaceae | *Simplicillium hymenopterorum* | DY101691 | MT453848 | MT471337 | - | MT471344 | - | Ant | Chen et al. (2022) |
| Hypocreomycetidae | Hypocreales | Cordycipitaceae | *Simplicillium lamellicola* | CBS 116.25 | AJ292393 | DQ522356 | AF339552 | DQ522404 | DQ522462 | *Agaricus bisporus* | Spatafora et al. (2007) |
| Hypocreomycetidae | Hypocreales | Cordycipitaceae | *Simplicillium lanosoniveum* | CBS 704.86 | AJ292396 | DQ522358 | AF339553 | DQ522406 | DQ522464 | *Hemileia vastatrix* | Spatafora et al. (2007) |
| Hypocreomycetidae | Hypocreales | Cordycipitaceae | *Simplicillium larvatum* | DY101731 | OM743438 | OM818462 | OM743441 | OM818460 | OM818461 | Lepidoptera larva | Chen et al. (2022) |
| Hypocreomycetidae | Hypocreales | Cordycipitaceae | *Simplicillium lepidopterorum* | GY29131 | MN006251 | MN022265 | - | MN022273 | - | Leafhopper | Chuang et al. (2024) |
| Hypocreomycetidae | Hypocreales | Cordycipitaceae | *Simplicillium lepidopterorum* | RCEF7273 | PV134468 | PV166467 | PV134526 | PV166539 | PV166570 | Araneae: spider | This study |
| Hypocreomycetidae | Hypocreales | Cordycipitaceae | *Simplicillium minatense* | JCM 18176 | AB603992 | LC496908 | LC496878 | - | - | Soil | Chuang et al. (2024) |
| Hypocreomycetidae | Hypocreales | Cordycipitaceae | *Simplicillium neolepidopterorum* | DY101751 | MT453854 | MT471339 | MT453855 | - | - | Lepidoptera | Chen et al. (2022) |
| Hypocreomycetidae | Hypocreales | Cordycipitaceae | *Simplicillium niveum* | BCC 83036 | MW621499 | MW603488 | MW620992 | MW603489 | - | *Ophiocordyceps camponoti-leonardi* | Chuang et al. (2024) |
| Hypocreomycetidae | Hypocreales | Cordycipitaceae | *Simplicillium pseudocercosporicola* | COAD 3687 | PP836713 | PP855562 | PQ115147 | PP855565 | - | Pseudocercospora fijiensis | Custódio and Pereira, (2025) |
| Hypocreomycetidae | Hypocreales | Cordycipitaceae | *Simplicillium salviniae* | BCRC 34536 | MT974200 | MW200240 | MT974415 | MW200244 | MW200248 | Salvinia auriculata | Chuang et al. (2024) |
| Hypocreomycetidae | Hypocreales | Cordycipitaceae | *Simplicillium scarabaeoidea* | DY101391 | MT453842 | MT471335 | MT453844 | MT471343 | - | Scarabaeoidea | Chen et al. (2023) |
| Hypocreomycetidae | Hypocreales | Cordycipitaceae | *Simplicillium scarabaeoidea* | RCEF7549 | PV134486 | PV166487 | PV134545 | PV166546 | PV166585 | Araneae: spider | This study |
| Hypocreomycetidae | Hypocreales | Cordycipitaceae | *Simplicillium sympodiophorum* | JCM 18184 | AB604003 | LC496912 | LC496882 | - | - | Soil | Chuang et al. (2024) |
| Hypocreomycetidae | Hypocreales | Cordycipitaceae | *Simplicillium yunnanense* | YFCC 7133 | - | MN576954 | MN576784 | MN576844 | - | Synnemata of Arachnidicola waltergamsii | Chuang et al. (2024) |
| Hypocreomycetidae | Hypocreales | Cordycipitaceae | *Zarea aleophilum* | CBS 357.80 | MH861274 | KM283815 | KM283791 | KM283835 | KM283856 | *Agaricus bitorquis* | Khonsanit et al. (2024) |
| Hypocreomycetidae | Hypocreales | Cordycipitaceae | *Zarea flavidum* | CBS 300.70D | MH859668 | KM283813 | KM283789 | - | KM283855 | *Coltricia perennis* | Khonsanit et al. (2024) |
| Hypocreomycetidae | Hypocreales | Cordycipitaceae | *Zarea fungicola* | CBS 992.69 | MH859503 | KM283816 | KM283792 | - | KM283857 | *Agaricus bisporus* | Khonsanit et al. (2024) |
| Hypocreomycetidae | Hypocreales | Cordycipitaceae | *Zouia cauligalbarum* | GZUIFRZHJ01 | MH730663 | MH801920 | MH730667 | MH801922 | MH801924 | Lepidoptera: Stemborer | Khonsanit et al. (2024) |
| Hypocreomycetidae | Hypocreales | Flammocladiellaceae | *Flammocladiella aceris* | CPC 24422 | KR611883 | MW890088 | KR611901 | - | MW890061 | Twigs of Acer platanoides | Crous et al. (2015) |
| Hypocreomycetidae | Hypocreales | Flammocladiellaceae | *Flammocladiella anomiae* | CLL16017 | MN597422 | MW890089 | MW883811 | - | MW890062 | Stromata of Massaria anomia | Lechat et al. (2019) |
| Hypocreomycetidae | Hypocreales | Flammocladiellaceae | *Flammocladiella decora* | CLL16020 | MF611693 | OQ470901 | MF614949 | - | - | *Massaria inquinans* | Lechat et al. (2019) |
| Hypocreomycetidae | Hypocreales | Hypocreaceae | *Cladobotryum indoafrum* | TFC 201295 | FN859403 | FN868721 | FN859403 | FN868784 | FN868657 | Neonothopanus sp. | Põldmaa (2011) |
| Hypocreomycetidae | Hypocreales | Hypocreaceae | *Escovopsis aspergilloides* | CBS 423.93 | NR_137160 | AY172632 | KF293283 | MT305421 | MT305546 | *Trachymyrmex ruthae* | Montoya et al. (2023) |
| Hypocreomycetidae | Hypocreales | Hypocreaceae | *Escovopsis moelleri* | CBS 135748 | JQ815077 | JQ855712 | JQ855715 | MT305413 | MT305538 | *Acromyrmex subterraneus molestans* | Montoya et al. (2023) |
| Hypocreomycetidae | Hypocreales | Hypocreaceae | *Hypomyces australasiaticus* | TFC 03-8 | FN859428 | FN868746 | FN859428 | FN868807 | FN868681 | *Earliella scabrosa* | Põldmaa (2011) |
| Hypocreomycetidae | Hypocreales | Hypocreaceae | *Hypomyces gabonensis* | TFC 201156 | FN859430 | FN868749 | FN859430 | FN868810 | FN868684 | *Rigidoporus lineatus* | Põldmaa (2011) |
| Hypocreomycetidae | Hypocreales | Hypocreaceae | *Kiflimonium cryptomeriae* | UESTCC 23.0247 | OR887401 | PP076827 | OR887112 | - | PP076816 | *Cryptomeria japonica* | Tian et al. (2024) |
| Hypocreomycetidae | Hypocreales | Hypocreaceae | *Trichoderma alpinum* | HMAS 248821 | KY687906 | KY688012 | - | - | KY687958 | *Diseased Gastrodiaelata* | Chen and Zhuang, (2017) |
| Hypocreomycetidae | Hypocreales | Hypocreaceae | *Trichoderma amazonicum* | CBS 126898 | HM142358 | HM142376 | JN939814 | JQ031091 | HM142367 | *Hevea brasiliensis* | Chaverri et al. (2011) |
| Hypocreomycetidae | Hypocreales | Hypocreaceae | *Trichoderma harzianum* | CBS 226.95 | AJ222720 | AF348101 | MH874152 | JQ031082 | AF545549 | Soil | Chen and Zhuang, (2017) |
| Hypocreomycetidae | Hypocreales | Hypocreaceae | *Trichoderma turrialbense* | CBS 100525 | AF057600 | AF534614 | JN939837 | JQ031084 | AF545541 | *Mushroom compost* | Chaverri et al. (2003) |
| Hypocreomycetidae | Hypocreales | Ijuhyaceae | *Ijuhya chilensis* | CBS 102803 | KY607538 | - | KY607553 | KY607579 | - | *Nolina micrantha* | Ashrafi et al. (2017) |
| Hypocreomycetidae | Hypocreales | Ijuhyaceae | *Ijuhya corynospora* | CBS 342.77 | KY607539 | - | KY607554 | KY607580 | - | *Phormium tenax* | Ashrafi et al. (2017) |
| Hypocreomycetidae | Hypocreales | Ijuhyaceae | *Ijuhya faveliana* | CBS 133850 | KY607541 | - | KY607556 | KY607582 | - | Palm | Ashrafi et al. (2017) |
| Hypocreomycetidae | Hypocreales | Ijuhyaceae | *Ijuhya parilis* | CBS 136677 | KY607543 | - | KY607558 | KY607584 | - | Genista sp. | Ashrafi et al. (2017) |
| Hypocreomycetidae | Hypocreales | Ijuhyaceae | *Ijuhya peristomialis* | CBS 569.76 | KY607544 | KY607559 | PV273678 | KY607585 | PV273463 | *Cyathea dealbata* | Ashrafi et al. (2017) |
| Hypocreomycetidae | Hypocreales | Myrotheciomycetaceae | *Myrotheciomyces corymbiae* | CPC 33206 | OR051628 | OQ471031 | MH327837 | - | - | *Corymbia variegata* | Crous et al. (2018) |
| Hypocreomycetidae | Hypocreales | Myrotheciomycetaceae | *Trichothecium hongkongense* | CBS 101444 | OQ429887 | OQ471219 | OQ430139 | - | OQ454288 | Living leaves | Hou et al. (2023) |
| Hypocreomycetidae | Hypocreales | Myrotheciomycetaceae | *Trichothecium roseum* | PGTR-3 | OQ996601 | *OR101078* | OQ996630 | - | OR100961 | *Psidium guajava* | Hou et al. (2023) |
| Hypocreomycetidae | Hypocreales | Nectriaceae | *Allantonectria miltina* | CBS 125499 | KM231836 | KM231974 | KM231717 | KM232270 | HQ897730 | *Yucca elata* | Gräfenhan et al. (2011) |
| Hypocreomycetidae | Hypocreales | Nectriaceae | *Campylocarpon pseudofasciculare* | CBS 112679 | AY677306 | JF735692 | HM364314 | HM364332 | KM232323 | *Vitis vinifera* | Lombard et al. (2015) |
| Hypocreomycetidae | Hypocreales | Nectriaceae | *Cosmospora cymosa* | CBS 762.69 | HQ897828 | KM231948 | KM231693 | KM232243 | HQ897778 | *Inonotus radiatus* | Lombard et al. (2015) |
| Hypocreomycetidae | Hypocreales | Nectriaceae | *Dialonectria episphaeria* | CBS 125494 | HQ897811 | KM231953 | KM231697 | KM232248 | HQ897756 | Unknown Ascomycete | Lombard et al. (2015) |
| Hypocreomycetidae | Hypocreales | Nectriaceae | *Fusarium pseudograminearum* | CBS 109956 | DQ459871 | AF212468 | DQ459871 | JX171524 | JX171637 | *Hordeum vulgare* | O’Donnell et al. (2013) |
| Hypocreomycetidae | Hypocreales | Nectriaceae | *Macroconia leptosphaeria* | CBS 717.74 | KM231827 | JF735695 | KM231707 | KM232257 | KM232390 | Stroma of Pyrenomycete | Lombard et al. (2015) |
| Hypocreomycetidae | Hypocreales | Nectriaceae | *Microcera rubra* | CBS 638.76 | HQ897820 | JF740696 | KM231702 | KM232253 | HQ897767 | *Quadrapidiotus perniciosus* | Lombard et al. (2015) |
| Hypocreomycetidae | Hypocreales | Nectriaceae | *Nalanthamala psidii* | CBS 116952 | AY864836 | KM231972 | AY864837 | KM232268 | KM232401 | *Psidium guajava* | Lombard et al. (2015) |
| Hypocreomycetidae | Hypocreales | Nectriaceae | *Nectria cinnabarina* | CBS 125165 | HM484548 | HM484527 | HM484562 | HM484577 | KM232402 | Aesculus sp. | Lombard et al. (2015) |
| Hypocreomycetidae | Hypocreales | Niessliaceae | *Eucasphaeria capensis* | CBS 120028 | MH863072 | OQ470896 | MH874626 | - | OQ453986 | Eucalyptus sp. | Hou et al. (2023) |
| Hypocreomycetidae | Hypocreales | Niessliaceae | *Eucasphaeria proteae* | CBS 146815 | MW175357 | MW173129 | MW175397 | - | MW173116 | *Protea neriifolia* | Crous et al. (2020) |
| Hypocreomycetidae | Hypocreales | Niessliaceae | *Niesslia minutispora* | CBS 246.82 | OQ429818 | MG896429 | MH878348 | - | OQ454216 | Agricultural soil | Hou et al. (2023) |
| Hypocreomycetidae | Hypocreales | Niessliaceae | *Niesslia trachycarpi* | GZCC21-0196 | PP578084 | PP761000 | PP621072 | - | PP780247 | *Trachycarpus fortunei* | Zhang et al. (2024a) |
| Hypocreomycetidae | Hypocreales | Niessliaceae | *Rosasphaeria moravica* | CBS 124270 | OQ429826 | JF440987 | OQ430087 | - | JF440986 | *Rosa canina* | Hou et al. (2023) |
| Hypocreomycetidae | Hypocreales | Niessliaceae | *Trichosphaerella ceratophora* | CBS 130.82 | KM231847 | KM231983 | KM231727 | KM232280 | KM232423 | *Carpinus betulus* | Hou et al. (2023) |
| Hypocreomycetidae | Hypocreales | Ophiocordycipitaceae | *Drechmeria gunni* | OSC 76404 | - | AY489616 | AF339522 | AY489650 | DQ522426 | Lepidoptera larva | Xu et al. (2025) |
| Hypocreomycetidae | Hypocreales | Ophiocordycipitaceae | *Drechmeria panacis* | SYPF 8335 | MF588878 | MF614144 | MF588897 | - | - | S root of Panax notoginseng | Yu et al. (2018) |
| Hypocreomycetidae | Hypocreales | Ophiocordycipitaceae | *Drechmeria sinensis* | CBS 567.95 | MH862540 | DQ522343 | MH874175 | DQ522389 | DQ522443 | Nematoda | Spatafora et al. (2007) |
| Hypocreomycetidae | Hypocreales | Ophiocordycipitaceae | *Drechmeria zeospora* | CBS 335.80 | MH861269 | EF469062 | AF339540 | EF469091 | EF469109 | *Panagroliamus subelongatus* | Sung et al. (2007) |
| Hypocreomycetidae | Hypocreales | Ophiocordycipitaceae | *Harposporium incensis* | ZBAH1472 | OQ170826 | OQ183327 | - | OQ186689 | OQ186691 | Larva of Trichophassus giganteus Lepidoptera: Hepialidae | Chen et al. (2025b) |
| Hypocreomycetidae | Hypocreales | Ophiocordycipitaceae | *Hirsutella cf. haptospora* | ARSEF 2228 | KM652166 | KM652001 | KM652118 | KM652041 | - | Diptera: Itonididae | Simmons et al. (2015) |
| Hypocreomycetidae | Hypocreales | Ophiocordycipitaceae | *Hirsutella citriformis* | ARSEF 1035 | KM652153 | KM651989 | KM652105 | KM652030 | - | Hemiptera: Cixiidae | Simmons et al. (2015) |
| Hypocreomycetidae | Hypocreales | Ophiocordycipitaceae | *Hirsutella cryptosclerotium* | ARSEF 4517 | KM652157 | KM651992 | KM652109 | KM652032 | - | Hemiptera: Pseudococcidae | Simmons et al. (2015) |
| Hypocreomycetidae | Hypocreales | Ophiocordycipitaceae | *Hirsutella flava* | GZUIFRhir1006271 | KY415598 | KY415601 | KY415599 | KY945366 | - | Lepidoptera: | Qu et al. (2021) |
| Hypocreomycetidae | Hypocreales | Ophiocordycipitaceae | *Hirsutella guyana* | ARSEF 878 | KM652158 | KM651994 | KM652111 | KM652035 | - | Hemiptera: Cicadellidae | Simmons et al. (2015) |
| Hypocreomycetidae | Hypocreales | Ophiocordycipitaceae | *Hirsutella illustris* | ARSEF 5539 | KM652160 | KM651996 | KM652112 | KM652037 | - | Hemiptera: Aphididae | Simmons et al. (2015) |
| Hypocreomycetidae | Hypocreales | Ophiocordycipitaceae | *Hirsutella kuankuoshuiensis* | GZUIFR 2012KKS31 | KY415575 | KY415590 | KY415582 | KY945360 | - | Lepidoptera | Qu et al. (2021) |
| Hypocreomycetidae | Hypocreales | Ophiocordycipitaceae | *Hirsutella lecaniicola* | ARSEF 8888 | KM652162 | KM651998 | KM652114 | KM652038 | - | Hemiptera: Coccidae | Simmons et al. (2015) |
| Hypocreomycetidae | Hypocreales | Ophiocordycipitaceae | *Hirsutella necatrix* | ARSEF 5549 | KM652164 | KM651999 | KM652116 | KM652039 | - | Acari | Simmons et al. (2015) |
| Hypocreomycetidae | Hypocreales | Ophiocordycipitaceae | *Hirsutella nodulosa* | ARSEF 5473 | KM652165 | KM652000 | KM652117 | KM652040 | - | Lepidoptera: Pyralidae | Simmons et al. (2015) |
| Hypocreomycetidae | Hypocreales | Ophiocordycipitaceae | *Hirsutella rhossiliensis* | ARSEF 3747 | KM652170 | KM652006 | KM652123 | KM652045 | - | Tylenchida: Criconematidae | Simmons et al. (2015) |
| Hypocreomycetidae | Hypocreales | Ophiocordycipitaceae | *Hirsutella satumaensis* | ARSEF 996 | KM652172 | KM652008 | KM652125 | KM652047 | - | Lepidoptera: Pyralidae | Simmons et al. (2015) |
| Hypocreomycetidae | Hypocreales | Ophiocordycipitaceae | *Hirsutella sinensis* | ARSEF 6282 | KM652173 | KM652009 | KM652126 | KM652048 | - | Lepidoptera: Hepialidae | Simmons et al. (2015) |
| Hypocreomycetidae | Hypocreales | Ophiocordycipitaceae | *Hirsutella strigosa* | ARSEF 2197 | KM652175 | KM652012 | KM652129 | KM652050 | - | Hemiptera: Cicadellidae | Sun et al. (2024) |
| Hypocreomycetidae | Hypocreales | Ophiocordycipitaceae | *Hirsutella subulata* | ARSEF 2227 | KM652176 | KM652013 | KM652130 | KM652051 | - | Lepidoptera: Microlepidoptera | Sun et al. (2024) |
| Hypocreomycetidae | Hypocreales | Ophiocordycipitaceae | *Hirsutella thompsonii* | ARSEF 3323 | KM652188 | KM652024 | KM652143 | KM652059 | - | Acari: Tenuipalpidae | Simmons et al. (2015) |
| Hypocreomycetidae | Hypocreales | Ophiocordycipitaceae | *Hirsutella thompsonii var. thompsonii* | ARSEF 137 | KM652177 | KM652014 | KM652131 | KM652052 | - | Acari: Eriophyidae | Simmons et al. (2015) |
| Hypocreomycetidae | Hypocreales | Ophiocordycipitaceae | *Ophiocordyceps acicularis* | ARSEF 5692 | JN049819 | DQ522322 | DQ518754 | DQ522368 | DQ522418 | Coleoptera | Tang et al. (2023) |
| Hypocreomycetidae | Hypocreales | Ophiocordycipitaceae | *Ophiocordyceps acroasca* | YFCC 9049 | - | ON567757 | ON555918 | ON568677 | ON568130 | Camponotus sp. | Tang et al. (2023) |
| Hypocreomycetidae | Hypocreales | Ophiocordycipitaceae | *Ophiocordyceps aphodii* | ARSEF 5498 | - | DQ522323 | DQ518755 | - | DQ522419 | Coleoptera | Guan et al. (2025) |
| Hypocreomycetidae | Hypocreales | Ophiocordycipitaceae | *Ophiocordyceps asiatica* | BCC 30516 | MH754722 | MK284263 | MH753675 | MK214105 | MK214091 | Termitidae adult termite | Tasanathai et al. (2019) |
| Hypocreomycetidae | Hypocreales | Ophiocordycipitaceae | *Ophiocordyceps basiasca* | YHH 20191 | - | ON567748 | ON555910 | ON568672 | ON568121 | Camponotus sp. | Tang et al. (2023) |
| Hypocreomycetidae | Hypocreales | Ophiocordycipitaceae | *Ophiocordyceps bifertilis* | YFCC 9013 | - | ON567768 | ON555926 | ON568148 | ON568138 | Polyrhachis sp. | Tang et al. (2023) |
| Hypocreomycetidae | Hypocreales | Ophiocordycipitaceae | *Ophiocordyceps blattae* | MFLU:220268 | OQ127355 | OQ186380 | OQ127389 | OQ186431 | OQ186405 | Cockroach nymphs lying on leaf litter | Wei et al. (2022) |
| Hypocreomycetidae | Hypocreales | Ophiocordycipitaceae | *Ophiocordyceps brunneipunctata* | OSC 128576 | - | DQ522324 | DQ518756 | DQ522369 | DQ522420 | Coleoptera | Tang et al. (2023) |
| Hypocreomycetidae | Hypocreales | Ophiocordycipitaceae | *Ophiocordyceps campes* | BCC36938 | MT783955 | MT118167 | MT118175 | MT118183 | MT118188 | Lepidoptera larva | Tasanathai et al. (2020) |
| Hypocreomycetidae | Hypocreales | Ophiocordycipitaceae | *Ophiocordyceps communis* | BCC 1842 | MH754726 | MK284266 | MH753680 | MK214110 | MK214096 | Termitidae | Mongkolsamrit et al. (2024) |
| Hypocreomycetidae | Hypocreales | Ophiocordycipitaceae | *Ophiocordyceps contiispora* | YFCC 9027 | - | ON567752 | ON555913 | ON568142 | ON568125 | Camponotus sp. | Tang et al. (2023) |
| Hypocreomycetidae | Hypocreales | Ophiocordycipitaceae | *Ophiocordyceps crinalis* | HKAS:102447 | OQ127357 | OQ186382 | OQ127391 | OQ186433 | OQ186406 | Lepidopteran larva | Wei et al. (2022) |
| Hypocreomycetidae | Hypocreales | Ophiocordycipitaceae | *Ophiocordyceps dipterigena* | OSC 151912 | - | KJ878967 | KJ878887 | KJ879001 | - | Diptera | Mongkolsamrit et al. (2025) |
| Hypocreomycetidae | Hypocreales | Ophiocordycipitaceae | *Ophiocordyceps formicarum* | TNSF 18565 | - | KJ878968 | KJ878888 | KJ879002 | KJ878946 | Hymenoptera | Tang et al. (2023) |
| Hypocreomycetidae | Hypocreales | Ophiocordycipitaceae | *Ophiocordyceps formosana* | TNMF 13893 | - | KJ878956 | - | KJ878988 | KJ878943 | Coleoptera | Sun et al. (2022) |
| Hypocreomycetidae | Hypocreales | Ophiocordycipitaceae | *Ophiocordyceps forquignonii* | OSC 151902 | - | - | KJ878876 | KJ878991 | KJ878945 | Diptera | Tang et al. (2023) |
| Hypocreomycetidae | Hypocreales | Ophiocordycipitaceae | *Ophiocordyceps fusiformis* | BCC 93025 | MZ676743 | MZ707849 | MZ675422 | MZ707855 | MZ707805 | Termites | Tasanathai et al. (2022) |
| Hypocreomycetidae | Hypocreales | Ophiocordycipitaceae | *Ophiocordyceps globosa* | BCC 93023 | MZ676740 | MZ707846 | MZ675419 | MZ707861 | - | Termites | Tasanathai et al. (2022) |
| Hypocreomycetidae | Hypocreales | Ophiocordycipitaceae | *Ophiocordyceps irangiensis* | OSC 128579 | - | EF469060 | EF469076 | EF469089 | EF469107 | Hymenoptera | Tang et al. (2023) |
| Hypocreomycetidae | Hypocreales | Ophiocordycipitaceae | *Ophiocordyceps isopterae* | MY12376 | MZ676741 | MZ707847 | MZ675420 | MZ707859 | MZ707803 | Termites | Tasanathai et al. (2022) |
| Hypocreomycetidae | Hypocreales | Ophiocordycipitaceae | *Ophiocordyceps kimflemingiae* | HUA 186148 | - | KC610739 | KF658679 | KF658667 | KC610717 | Hymenoptera | Tang et al. (2023) |
| Hypocreomycetidae | Hypocreales | Ophiocordycipitaceae | *Ophiocordyceps konnoana* | EFCC 7315 | - | EF468753 | - | EF468861 | EF468916 | Coleoptera | Tang et al. (2023) |
| Hypocreomycetidae | Hypocreales | Ophiocordycipitaceae | *Ophiocordyceps lloydii* | OSC 151913 | - | KJ878970 | KJ878891 | KJ879004 | KJ878948 | Hymenoptera | Tang et al. (2023) |
| Hypocreomycetidae | Hypocreales | Ophiocordycipitaceae | *Ophiocordyceps longissima* | NBRC 106965 | AB968406 | AB968584 | AB968420 | - | AB968546 | Hemiptera; cicada nymph | Sun et al. (2022) |
| Hypocreomycetidae | Hypocreales | Ophiocordycipitaceae | *Ophiocordyceps longistipes* | KUNCC 5224 | OR015962 | OR030530 | OR015967 | OR062224 | OR113082 | Termites | Fan et al. (2024) |
| Hypocreomycetidae | Hypocreales | Ophiocordycipitaceae | *Ophiocordyceps melolonthae* | OSC 110993 | - | DQ522331 | DQ518762 | DQ522376 | - | Coleoptera | Spatafora et al. (2007) |
| Hypocreomycetidae | Hypocreales | Ophiocordycipitaceae | *Ophiocordyceps mosingtoensis* | BCC 30904 | MH754732 | MK284273 | MH753686 | MK214115 | MK214100 | Termitidaeadult termite | Tasanathai et al. (2022) |
| Hypocreomycetidae | Hypocreales | Ophiocordycipitaceae | *Ophiocordyceps nigrella* | EFCC 9247 | JN049853 | EF468758 | EF468818 | EF468866 | EF468920 | Lepidopteran larva | Tang et al. (2023) |
| Hypocreomycetidae | Hypocreales | Ophiocordycipitaceae | *Ophiocordyceps nooreniae* | BRIP 55363 | - | KX673812 | NG059720 | - | KX673809 | Chariomyrma | Tang et al. (2023) |
| Hypocreomycetidae | Hypocreales | Ophiocordycipitaceae | *Ophiocordyceps nuozhaduensis* | YHH 20168 | - | ON567769 | ON555927 | ON568683 | - | Camponotus sp. | Tang et al. (2023) |
| Hypocreomycetidae | Hypocreales | Ophiocordycipitaceae | *Ophiocordyceps nutans* | OSC 110994 | - | DQ522333 | DQ518763 | DQ522378 | - | Hemiptera | Spatafora et al. (2007) |
| Hypocreomycetidae | Hypocreales | Ophiocordycipitaceae | *Ophiocordyceps ootakii* | J13 | - | KX713681 | KX713600 | KX713708 | - | *Polyrhachis moesta* | Tang et al. (2023) |
| Hypocreomycetidae | Hypocreales | Ophiocordycipitaceae | *Ophiocordyceps ovatospora* | YHH 2206001 | OP295105 | OP313801 | OP295113 | OP313803 | OP313805 | Termitidaeadult termite | Xu et al. (2025) |
| Hypocreomycetidae | Hypocreales | Ophiocordycipitaceae | *Ophiocordyceps phuwiangensis* | BCC 85351 | MT783958 | MT118174 | - | MT118187 | MT118195 | Lepidoptera | Xu et al. (2025) |
| Hypocreomycetidae | Hypocreales | Ophiocordycipitaceae | *Ophiocordyceps ponerinarum* | HUA 186140T | - | KC610740 | KC610767 | KF658668 | - | Paraponera clavata | Tang et al. (2023) |
| Hypocreomycetidae | Hypocreales | Ophiocordycipitaceae | *Ophiocordyceps pseudocommunis* | BCC 16757 | MH754733 | MK284274 | MH753687 | MK214117 | MK214101 | Termitidaeadult termite | Tasanathai et al. (2019) |
| Hypocreomycetidae | Hypocreales | Ophiocordycipitaceae | *Ophiocordyceps pseudorhizoidea* | BCC 48879 | MH754720 | MK284261 | MH753673 | MK214104 | MK214089 | Termitidaeadult termite | Tasanathai et al. (2019) |
| Hypocreomycetidae | Hypocreales | Ophiocordycipitaceae | *Ophiocordyceps purpureostromata* | TNSF 18430 | - | KJ878977 | KJ878897 | KJ879011 | - | Coleoptera | Tang et al. (2023) |
| Hypocreomycetidae | Hypocreales | Ophiocordycipitaceae | *Ophiocordyceps radiciformis* | BCC 93036 | MZ676746 | MZ707852 | MZ675425 | MZ707857 | MZ707808 | Termites | Tasanathai et al. (2022) |
| Hypocreomycetidae | Hypocreales | Ophiocordycipitaceae | *Ophiocordyceps ravenelii* | OSC 151914 | - | KJ878978 | - | KJ879012 | KJ878950 | Coleoptera | Tang et al. (2023) |
| Hypocreomycetidae | Hypocreales | Ophiocordycipitaceae | *Ophiocordyceps rhizoidea* | NHJ 12522 | JN049857 | EF468764 | EF468825 | EF468873 | EF468923 | Coleoptera | Tang et al. (2023) |
| Hypocreomycetidae | Hypocreales | Ophiocordycipitaceae | *Ophiocordyceps rubiginosiperitheciata* | NBRC 100946 | JN943344 | AB968582 | JN941437 | JN992438 | AB968544 | Coleoptera | Sun et al. (2024) |
| Hypocreomycetidae | Hypocreales | Ophiocordycipitaceae | *Ophiocordyceps satoi* | J19 | - | KX713684 | KX713601 | KX713710 | - | *Polyrhachis lamellidens* | Tang et al. (2023) |
| Hypocreomycetidae | Hypocreales | Ophiocordycipitaceae | *Ophiocordyceps sinensis* | EFCC 7287 | JN049854 | EF468767 | EF468827 | EF468874 | EF468924 | Lepidoptera | Tang et al. (2023) |
| Hypocreomycetidae | Hypocreales | Ophiocordycipitaceae | *Ophiocordyceps sobolifera* | KEW 78842 | JN049855 | - | EF468828 | EF468875 | EF468925 | Hemiptera | Tang et al. (2023) |
| Hypocreomycetidae | Hypocreales | Ophiocordycipitaceae | *Ophiocordyceps spataforae* | OSC 128575 | JN049845 | EF469064 | EF469079 | EF469093 | EF469110 | Hemipteran adult | Sun et al. (2024) |
| Hypocreomycetidae | Hypocreales | Ophiocordycipitaceae | *Ophiocordyceps sphecocephala* | NBRC 101752 | JN943351 | AB968591 | JN941445 | JN992430 | AB968552 | Hymenoptera | Sun et al. (2024) |
| Hypocreomycetidae | Hypocreales | Ophiocordycipitaceae | *Ophiocordyceps stylophora* | OSC 110999 | - | EF468777 | EF468837 | EF468882 | EF468931 | Coleoptera | Tang et al. (2023) |
| Hypocreomycetidae | Hypocreales | Ophiocordycipitaceae | *Ophiocordyceps subtiliphialida* | YFCC 8815 | - | ON567753 | ON555914 | ON568673 | ON568126 | Camponotus sp. | Tang et al. (2023) |
| Hypocreomycetidae | Hypocreales | Ophiocordycipitaceae | *Ophiocordyceps termiticola* | BCC 1920 | MH754724 | MK284265 | MH753678 | MK214108 | MK214094 | Termitidae adult termite | Mongkolsamrit et al. (2025) |
| Hypocreomycetidae | Hypocreales | Ophiocordycipitaceae | *Ophiocordyceps thanathonensis* | MFLU 16-2909 | MF850376 | MF872613 | MF850377 | MF872615 | - | Hymenotera | Xu et al. (2025) |
| Hypocreomycetidae | Hypocreales | Ophiocordycipitaceae | *Ophiocordyceps unilateralis* | VIC 44303 | - | KX713675 | KX713626 | KX713730 | - | Camponotus | Tang et al. (2023) |
| Hypocreomycetidae | Hypocreales | Ophiocordycipitaceae | *Ophiocordyceps yakusimensis* | HMAS 199604 | - | - | KJ878902 | KJ879018 | KJ878953 | Hemiptera | Tang et al. (2023) |
| Hypocreomycetidae | Hypocreales | Ophiocordycipitaceae | *Paraisaria alba* | HKAS 102484 | MN947219 | MN929085 | MN943839 | MN929078 | MN929082 | Orthoptera | Chen et al. (2025a) |
| Hypocreomycetidae | Hypocreales | Ophiocordycipitaceae | *Paraisaria amazonica* | HUA 186143 | - | KM411989 | KJ917571 | KP212902 | KM411982 | Orthoptera | Chen et al. (2025a) |
| Hypocreomycetidae | Hypocreales | Ophiocordycipitaceae | *Paraisaria arcta* | HKAS 102552 | MN947220 | MN929086 | MN943840 | MN929079 | MN929083 | Lepidoptera | Chen et al. (2025a) |
| Hypocreomycetidae | Hypocreales | Ophiocordycipitaceae | *Paraisaria cascadensis* | OSCM052010 | OQ709237 | OR199814 | OQ708931 | OR199828 | OR199838 | Orthoptera | Chen et al. (2025a) |
| Hypocreomycetidae | Hypocreales | Ophiocordycipitaceae | *Paraisaria coenomyia* | NBRC 106964 | AB968397 | AB968571 | AB968413 | - | AB968533 | Diptera | Chen et al. (2025a) |
| Hypocreomycetidae | Hypocreales | Ophiocordycipitaceae | *Paraisaria gracilis* | EFCC 8572 | JN049851 | EF468751 | EF468811 | EF468859 | EF468912 | Lepidoptera | Tehan et al. (2023) |
| Hypocreomycetidae | Hypocreales | Ophiocordycipitaceae | *Paraisaria heteropoda* | EFCC 10125 | JN049852 | EF468752 | EF468812 | EF468860 | EF468914 | Hemiptera | Tehan et al. (2023) |
| Hypocreomycetidae | Hypocreales | Ophiocordycipitaceae | *Paraisaria insignis* | OSC.164134 | OQ709231 | OR199807 | OQ708924 | OR199822 | - | Coleoptera | Tehan et al. (2023) |
| Hypocreomycetidae | Hypocreales | Ophiocordycipitaceae | *Paraisaria orthopterorum* | BBC 88305 | MH754742 | MK214080 | MK332583 | MK214084 | - | Orthoptera | Sun et al. (2024) |
| Hypocreomycetidae | Hypocreales | Ophiocordycipitaceae | *Paraisaria phuwiangensis* | TBRC 9709 | MK192015 | MK214082 | MK192057 | MK214086 | - | Coleoptera | Xu et al. (2025) |
| Hypocreomycetidae | Hypocreales | Ophiocordycipitaceae | *Paraisaria pseudoheteropoda* | OSCM052009 | OQ709241 | OR199818 | OQ708935 | OR199832 | OR199840 | Hemiptera | Tehan et al. (2023) |
| Hypocreomycetidae | Hypocreales | Ophiocordycipitaceae | *Paraisaria rosea* | HKAS 102546 | MN947222 | MN929088 | MN943842 | MN929081 | MN929084 | Coleoptera | Tehan et al. (2023) |
| Hypocreomycetidae | Hypocreales | Ophiocordycipitaceae | *Paraisaria sp.* | OSCM052011 | OQ709238 | OR199815 | OQ708932 | OR199829 | OR199839 | Insecta | Tehan et al. (2023) |
| Hypocreomycetidae | Hypocreales | Ophiocordycipitaceae | *Paraisaria yodhathaii* | TBRC 8502 | MH188540 | MH211354 | MH201168 | MH211350 | - | Coleoptera | Tehan et al. (2023) |
| Hypocreomycetidae | Hypocreales | Ophiocordycipitaceae | *Purpureocillium araneicola* | RCEF7731 | PV134503 | PV166502 | PV134562 | PV166552 | PV166597 | Araneae: spider | This study |
| Hypocreomycetidae | Hypocreales | Ophiocordycipitaceae | *Purpureocillium atypicola* | KUNCC23-13355 | OR910610 | OR920384 | OR910613 | OR920387 | - | Araneae: spider | Chang et al. (2024) |
| Hypocreomycetidae | Hypocreales | Ophiocordycipitaceae | *Purpureocillium atypicola* | RCEF7274 | PV134469 | PV166468 | PV134527 | - | - | Araneae: spider | This study |
| Hypocreomycetidae | Hypocreales | Ophiocordycipitaceae | *Purpureocillium jiangxiense* | JX17D04 | PP555636 | PP658209 | PP555645 | - | - | Soil | Chen et al. (2024) |
| Hypocreomycetidae | Hypocreales | Ophiocordycipitaceae | *Purpureocillium lavendulum* | FMR 10376 | FR734106 | FR775516 | FR775489 | FR775512 | FR775538 | Soil | Perdomo et al. (2013) |
| Hypocreomycetidae | Hypocreales | Ophiocordycipitaceae | *Purpureocillium lilacinum* | JCM 8438 | FR734105 | FR775515 | FR775488 | FR775511 | FR775537 | *Heterodera zeae* | Perdomo et al. (2013) |
| Hypocreomycetidae | Hypocreales | Ophiocordycipitaceae | *Purpureocillium lilacinum* | FMR 10380 | FR734102 | FR734157 | FR775485 | FR775508 | FR775534 | Soil | Perdomo et al. (2013) |
| Hypocreomycetidae | Hypocreales | Ophiocordycipitaceae | *Purpureocillium lilacinum* | RCEF7800 | PV134508 | PV166507 | PV134567 | PV166556 | PV166600 | Araneae: spider | This study |
| Hypocreomycetidae | Hypocreales | Ophiocordycipitaceae | *Purpureocillium roseum* | IOM 325363.1 | MT560195 | - | MT560197 | - | - | Human | Chen et al. (2024) |
| Hypocreomycetidae | Hypocreales | Ophiocordycipitaceae | *Purpureocillium sodanum* | SD19B01 | PP385438 | - | PP381492 | - | - | Soil | Unpublished |
| Hypocreomycetidae | Hypocreales | Ophiocordycipitaceae | *Purpureocillium takamizusanense* | RCEF4811 | MT568626 | MT583722 | MW718253 | MW723137 | MW723158 | Hemiptera: cicada | Lin et al. (2023b) |
| Hypocreomycetidae | Hypocreales | Ophiocordycipitaceae | *Purpureocillium zongqii* | TK042 | PQ211281 | PQ223682 | PQ211285 | - | - | Soil | Chen et al. (2024) |
| Hypocreomycetidae | Hypocreales | Ophiocordycipitaceae | *Tolypocladium amazonense* | MS308 | JQ905653 | KF747099 | KF747134 | KF747214 | - | *Hevea brasiliensis* | Gazis et al. (2014) |
| Hypocreomycetidae | Hypocreales | Ophiocordycipitaceae | *Tolypocladium capitatum* | NBRC 100997 | JN943313 | AB968597 | JN941401 | JN992474 | AB968558 | Elaphomyces sp. | Sun et al. (2024) |
| Hypocreomycetidae | Hypocreales | Ophiocordycipitaceae | *Tolypocladium cylindrosporum* | YFCC 1805001 | MK984581 | MK984569 | MK984577 | MK984584 | MK984573 | *Ophiocordyceps sinensis* | Chen et al. (2025a) |
| Hypocreomycetidae | Hypocreales | Ophiocordycipitaceae | *Tolypocladium endophyticum* | MX486 | KF747245 | KF747116 | KF747152 | KF747232 | - | *Hevea brasiliensis* | Gazis et al. (2014) |
| Hypocreomycetidae | Hypocreales | Ophiocordycipitaceae | *Tolypocladium fractum* | OSC 110990 | - | DQ522328 | DQ518759 | DQ522373 | DQ522425 | Elaphomyces | Spatafora et al. (2007) |
| Hypocreomycetidae | Hypocreales | Ophiocordycipitaceae | *Tolypocladium inegoense* | SU-15 | - | DQ118752 | DQ118741 | DQ127243 | - | Insect | Chaverri et al. (2005) |
| Hypocreomycetidae | Hypocreales | Ophiocordycipitaceae | *Tolypocladium inflatum* | OSC 71235 | JN049844 | EF469061 | EF469077 | EF469090 | EF469108 | Insect | Tehan et al. (2023) |
| Hypocreomycetidae | Hypocreales | Ophiocordycipitaceae | *Tolypocladium inusitaticapitatum* | HKAS 112153 | MW537736 | MW507528 | MW537719 | - | MW507530 | Elaphomyces sp. | Yu et al. (2021) |
| Hypocreomycetidae | Hypocreales | Ophiocordycipitaceae | *Tolypocladium japonicum* | NBRC 9647 | OP207722 | OP223146 | OP207732 | OP223124 | OP223134 | Elaphomyces sp. | Dong et al. (2022a) |
| Hypocreomycetidae | Hypocreales | Ophiocordycipitaceae | *Tolypocladium jezoense* | NBRC 106328 | OP207723 | OP223147 | OP207733 | OP223125 | OP223135 | Elaphomyces sp. | Dong et al. (2022a) |
| Hypocreomycetidae | Hypocreales | Ophiocordycipitaceae | *Tolypocladium ophioglossoides* | CBS 100239 | - | KJ878958 | KJ878874 | KJ878990 | KJ878944 | Elaphomyces sp. | Sun et al. (2024) |
| Hypocreomycetidae | Hypocreales | Ophiocordycipitaceae | *Tolypocladium paradoxum* | YFCC 882 | OP207724 | OP223148 | OP207734 | OP223126 | OP223136 | Insect | Dong et al. (2022a) |
| Hypocreomycetidae | Hypocreales | Ophiocordycipitaceae | *Tolypocladium pseudoalbum* | YFCC 875 | OP207725 | OP223151 | OP207737 | OP223129 | OP223139 | Soil | Chen et al. (2025a) |
| Hypocreomycetidae | Hypocreales | Ophiocordycipitaceae | *Tolypocladium reniformisporum* | YFCC 1805002 | MK984582 | MK984570 | MK984578 | MK984585 | MK984574 | *Ophiocordyceps sinensis* | Wang et al. (2022b) |
| Hypocreomycetidae | Hypocreales | Ophiocordycipitaceae | *Tolypocladium tropicale* | CBS 136897 | KF747254 | KF747090 | KF747125 | KF747204 | - | *Hevea brasiliensis* | Gazis et al. (2014) |
| Hypocreomycetidae | Hypocreales | Ophiocordycipitaceae | *Tolypocladium yunnanense* | YFCC 877 | OP207730 | OP223153 | OP207739 | OP223131 | - | Soil | Dong et al. (2022a) |
| Hypocreomycetidae | Hypocreales | Ophiocordycipitaceae | *Torrubiellomyces zombiae* | NY4434801 | - | ON513396 | - | ON513398 | - | *Ophiocordyceps camponoti-flordani* | Tehan et al. (2023) |
| Hypocreomycetidae | Hypocreales | Polycephalomycetaceae | *Dingleyomyces lloydii* | PDD1212154 | OR602634 | OR588853 | OR602640 | OR588860 | OR588858 | *Ophiocordyceps hauturu* | Wang et al. (2024b) |
| Hypocreomycetidae | Hypocreales | Polycephalomycetaceae | *Paradingleyomyces lepidopterorum* | HKAS 131927 | OR878364 | OR880679 | OR828239 | - | - | Stromata of Perennicordyceps cf.elaphomyceticola | Wang et al. (2024b) |
| Hypocreomycetidae | Hypocreales | Polycephalomycetaceae | *Perennicordyceps cuboidea* | NBRC 103836 | JN943332 | AB972951 | JN941420 | JN992455 | AB972955 | Larva of beetle | Chen et al. (2023) |
| Hypocreomycetidae | Hypocreales | Polycephalomycetaceae | *Perennicordyceps elaphomyceticola* | MFLU 21-0262 | OQ172064 | OQ459718 | OQ172032 | OQ459747 | OQ459792 | Elaphomyces sp. | Chen et al. (2023) |
| Hypocreomycetidae | Hypocreales | Polycephalomycetaceae | *Perennicordyceps paracuboidea* | NBRC 101742 | JN943338 | KF049685 | JN941431 | JN992444 | KF049669 | Larva of beetle | Chen et al. (2023) |
| Hypocreomycetidae | Hypocreales | Polycephalomycetaceae | *Perennicordyceps prolifica* | NBRC 101750 | JN943340 | AB972953 | JN941433 | JN992442 | AB972957 | Larva of Tanna  japonensis | Chen et al. (2023) |
| Hypocreomycetidae | Hypocreales | Polycephalomycetaceae | *Perennicordyceps ryogamiensis* | NBRC 101751 | JN943343 | KF049688 | JN941438 | JN992437 | - | Larva of beetle | Chen et al. (2023) |
| Hypocreomycetidae | Hypocreales | Polycephalomycetaceae | *Perennicordyceps zongqii* | DY05421 | PQ211278 | PQ223679 | PQ211282 | - | PQ223677 | Larva of moth | Chen et al. (2023) |
| Hypocreomycetidae | Hypocreales | Polycephalomycetaceae | *Pleurocordyceps aurantiacus* | MFLUCC 17-2113 | MG136916 | MG136874 | MG136910 | MG136866 | MG136870 | *Ophiocordyceps barnesii* | Xiao et al. (2018) |
| Hypocreomycetidae | Hypocreales | Polycephalomycetaceae | *Pleurocordyceps formosus* | ARSEF 1424 | KF049661 | KF049689 | KF049634 | KF049651 | KF049671 | Coleoptera | Wang et al. (2015) |
| Hypocreomycetidae | Hypocreales | Polycephalomycetaceae | *Pleurocordyceps heilongtanensis* | KUMCC 3008 | OQ172091 | OQ459731 | OQ172063 | OQ459759 | OQ459805 | Lepidoptera | Xiao et al. (2023) |
| Hypocreomycetidae | Hypocreales | Polycephalomycetaceae | *Pleurocordyceps litangensis* | YFCC 06109296 | PP410599 | PP550105 | PP410595 | PP697753 | PP550108 | *Ophiocordyceps sinensis* | Liu et al. (2024) |
| Hypocreomycetidae | Hypocreales | Polycephalomycetaceae | *Pleurocordyceps marginaliradians* | MFLU 17-1582 | MG136920 | MG136878 | MG136914 | MG136869 | MG271931 | Cossidae | Xiao et al. (2018) |
| Hypocreomycetidae | Hypocreales | Polycephalomycetaceae | *Pleurocordyceps nipponicus* | BCC 18108 | KF049657 | MF416517 | MF416569 | MF416676 | MF416462 | Neuroptera | Wang et al. (2015) |
| Hypocreomycetidae | Hypocreales | Polycephalomycetaceae | *Pleurocordyceps nutantis* | MFLU 21-0275 | OQ172073 | OQ459739 | OQ172048 | OQ459765 | OQ459811 | *Ophiocordyceps nutans* | Xiao et al. (2023) |
| Hypocreomycetidae | Hypocreales | Polycephalomycetaceae | *Pleurocordyceps parvicapitata* | MFLU 21-0271 | OQ172083 | OQ459723 | OQ172055 | OQ459752 | OQ459797 | Elaphomyces sp. | Xiao et al. (2023) |
| Hypocreomycetidae | Hypocreales | Polycephalomycetaceae | *Pleurocordyceps sinensis* | GACP 20-2305 | OQ172075 | OQ459725 | OQ172045 | OQ459753 | OQ459799 | *Ophiocordyceps barnesii* | Xiao et al. (2023) |
| Hypocreomycetidae | Hypocreales | Polycephalomycetaceae | *Pleurocordyceps vitellina* | KUMCC 3007 | OQ172090 | OQ459730 | OQ172062 | OQ459758 | OQ459804 | *Ophiocordyceps nigrella* | Xiao et al. (2023) |
| Hypocreomycetidae | Hypocreales | Polycephalomycetaceae | *Pleurocordyceps yunnanensis* | YHC PY1005 | KF977848 | KF977850 | KF977848 | KF977852 | KF977854 | *Ophiocordyceps nutans* | Wang et al. (2015) |
| Hypocreomycetidae | Hypocreales | Polycephalomycetaceae | *Polycephalomyces albiramus* | GACP 21-XS08 | OQ172092 | OQ459735 | OQ172037 | OQ459761 | OQ459807 | *Ophiocordyceps barnesii* | Xiao et al. (2023) |
| Hypocreomycetidae | Hypocreales | Polycephalomycetaceae | *Polycephalomyces formosus* | NBRC 109993 | MN586833 | MN598057 | MN586842 | MN598048 | MN598064 | Larvae of Coleoptera | Chen et al. (2023) |
| Hypocreomycetidae | Hypocreales | Polycephalomycetaceae | *Polycephalomyces jinghongensis* | YFCC 02959283 | PP274089 | PP581803 | PP274109 | PP697747 | PP581819 | Ophiocordyceps sp. | Liu et al. (2024) |
| Hypocreomycetidae | Hypocreales | Polycephalomycetaceae | *Polycephalomyces multiperitheciatae* | YFCC 06149288 | PP274098 | PP581798 | PP274114 | PP697743 | PP581815 | *Ophiocordyceps multiperitheciata* | Liu et al. (2024) |
| Hypocreomycetidae | Hypocreales | Polycephalomycetaceae | *Polycephalomyces myrmecophilus* | YFCC 09289443 | PP410602 | PP581795 | PP410605 | PP697740 | PP581812 | *Ophiocordyceps acroasca* | Liu et al. (2024) |
| Hypocreomycetidae | Hypocreales | Pseudodiploösporeaceae | *Pseudodiploospora longispora* | CGMCC 3.23769 | OP231758 | OP265138 | OP231750 | - | OP243572 | Fruiting body of cultivated Morchella spp. | Sun et al. (2023) |
| Hypocreomycetidae | Hypocreales | Pseudodiploösporeaceae | *Zelopaecilomyces penicillatus* | CBS 448.69 | JX012226 | - | - | MF416674 | - | Peridia of Arcyria cinerea | Kepler et al. (2017) |
| Hypocreomycetidae | Hypocreales | Sarocladiaceae | *Parasarocladium breve* | CBS 150.62 | OQ429781 | OQ471107 | OQ055677 | - | OQ454192 | Soil | Hou et al. (2023) |
| Hypocreomycetidae | Hypocreales | Sarocladiaceae | *Parasarocladium chondroidum* | CBS 652.93 | OQ429785 | OQ471111 | OQ055681 | - | OQ454196 | Gramineae | Hou et al. (2023) |
| Hypocreomycetidae | Hypocreales | Sarocladiaceae | *Parasarocladium sinense* | CGMCC 3.25521 | OR680550 | OR865900 | OR680617 | - | OR842965 | Soil | Zhang et al. (2024a) |
| Hypocreomycetidae | Hypocreales | Sarocladiaceae | *Sarocladium citri* | CBS 145044 | OQ429834 | OQ471164 | OQ430093 | - | OQ454233 | *Citrus sinensis* | Hou et al. (2023) |
| Hypocreomycetidae | Hypocreales | Sarocladiaceae | *Sarocladium fuscum* | CBS 334.80 | OQ429838 | OQ471168 | OQ430097 | - | OQ454237 | Bambusa sp. | Hou et al. (2023) |
| Hypocreomycetidae | Hypocreales | Sarocladiaceae | *Sarocladium ochraceum* | CBS 428.67 | OQ429846 | OQ471176 | HQ232070 | - | OQ454245 | *Zea mays* | Hou et al. (2023) |
| Hypocreomycetidae | Hypocreales | Stachybotryaceae | *Achroiostachys betulicola* | CBS 136397 | KU845792 | KU845848 | KU845812 | - | KU845831 | Root of Betula lutea | Lombard et al. (2016) |
| Hypocreomycetidae | Hypocreales | Stachybotryaceae | *Achroiostachys levigata* | CBS 185.79 | KU845805 | KU845860 | KU845825 | - | KU845841 | Soil in citrus field | Lombard et al. (2016) |
| Hypocreomycetidae | Hypocreales | Stachybotryaceae | *Alfaria elegiae* | CPC 45984 | PP791425 | PP780618 | PP791453 | - | PP780611 | *Elegia ebracteata* | Crous et al. (2024) |
| Hypocreomycetidae | Hypocreales | Stachybotryaceae | *Alfaria thymi* | CBS 447.83 | KU845990 | KU846013 | KU845999 | - | KU846006 | *Thymus serpyllum* | Lombard et al. (2016) |
| Hypocreomycetidae | Hypocreales | Stachybotryaceae | *Cymostachys coffeicola* | CBS 252.76 | KU846052 | KU846097 | MH872746 | - | KU846081 | *Coffea arabica* | Lombard et al. (2016) |
| Hypocreomycetidae | Hypocreales | Stachybotryaceae | *Peethambara sundara* | CBS 646.77 | KU846471 | KU846531 | MH872865 | - | KU846509 | *Macaranga indica* | Lombard et al. (2016) |
| Hypocreomycetidae | Hypocreales | Stachybotryaceae | *Stachybotrys subsylvatica* | CBS 126205 | KU846741 | KU847064 | KU846854 | - | KU846964 | Soil | Lombard et al. (2016) |
| Hypocreomycetidae | Hypocreales | Stachybotryaceae | *Striatibotrys yuccae* | CBS 390.68 | KU846770 | KU847093 | KU846884 | - | KU846989 | *Yucca flaccida* | Lombard et al. (2016) |
| Hypocreomycetidae | Hypocreales | Stromatonectriaceae | *Stromatonectria caraganae* | CBS 125579 | MH863716 | *HQ112286* | MH875179 | - | HQ112290 | Branches of Colutea arborescens | Vu et al. (2019) |
| Hypocreomycetidae | Hypocreales | Tilachlidiaceae | *Psychronectria hyperantarctica* | WA52045 | MF162269 | MF278999 | MF162270 | - | - | Bryum sp. | Pawłowska et al. (2017) |
| Hypocreomycetidae | Hypocreales | Tilachlidiaceae | *Septofusidium herbarum* | CBS 265.58 | KM231842 | KM231979 | KM231723 | KM232275 | KM232418 | *Urtica dioica* | Lombard et al. (2015) |
| Hypocreomycetidae | Hypocreales | Tilachlidiaceae | *Septofusidium stevensiae* | BRIP 72951a | OP599634 | - | OP598069 | - | - | Insecta | Tan and Shivas (2022) |
| Hypocreomycetidae | Hypocreales | Tilachlidiaceae | *Tilachlidium brachiatum* | CBS 505.67 | KM231839 | KM231976 | KM231720 | KM232272 | KM232415 | *Hypholoma fasciculare* | Lombard et al. (2015) |
| Hypocreomycetidae | Hypocreales | Tilachlidiaceae | *Tilachlidium brachiatum* | CBS 363.97 | KM231838 | KM231975 | KM231719 | KM232271 | KM232414 | Agaricus sp. | Lombard et al. (2015) |
| Hypocreomycetidae | Hypocreales | Xanthonectriaceae | *Bullanockia australis* | CPC 28976 | KY173415 | OQ470804 | KY173506 | - | OQ451835 | Leaves of Kingia australis | Hou et al. (2023) |
| Hypocreomycetidae | Hypocreales | Xanthonectriaceae | *Xanthonectria pseudopeziza* | CBS 140160 | KU593584 | OQ471291 | KU593583 | - | OQ454358 | *Suaeda vera* | Crous et al. (2018) |
| Sordariomycetidae | Amphisphaeriales | Apiosporaceae | *Apiospora marianiae* | AP18219 | ON692406 | ON677180 | ON692422 | - | ON677176 | *Phleum pratense* | Pintos and Alvarado, (2022) |
| Sordariomycetidae | Conioscyphales | Conioscyphaceae | *Conioscypha tenebrosa* | MFLU 19-0688 | MK804506 | - | MK804508 | - | MK828514 | Decaying wood | Liu et al. (2019) |
| Sordariomycetidae | Diaporthales | Diaporthaceae | *Ophiodiaporthe cyatheae* | HAST 1364 | JX570889 | KC465406 | JX570891 | - | JX570893 | *Cyathea lepifera* | Fu et al. (2013) |
| Sordariomycetidae | Savoryellales | Savoryellaceae | *Ascotaiwania lignicola* | NIL00005 | HQ446341 | HQ446307 | HQ446364 | - | HQ446419 | Dead wood | Boonyuen et al. (2011) |
| Sordariomycetidae | Sordariales | Bombardiaceae | *Ramophialophora petraea* | CGMCC:3.17952 | KU746702 | KX855247 | KU746748 | KY883222 | KY883253 | Plant debris | Zhang et al. (2017) |
| Sordariomycetidae | Sordariales | Lasiosphaeriaceae | *Bombardia bombarda* | AFTOL-ID 967 | - | DQ471095 | DQ470970 | DQ842035 | DQ470923 | Plant | Spatafora et al. (2006) |
| Sordariomycetidae | Sordariales | Lasiosphaeriaceae | *Immersiella caudata* | HKAS 92795 | MK828598 | MN194094 | MK835795 | - | MN156525 | Submerged wood | Luo et al. (2019) |
| Xylariomycetidae | Amphisphaeriales | Oxydothidaceae | *Oxydothis metroxylonicola* | MFLUCC 15-0281 | KY206774 | KY206778 | KY206763 | - | KY206781 | *Metroxylon sagu* | Konta et al. (2016) |
| Xylariomycetidae | Amphisphaeriales | Pestalotiopsidaceae | *Ciliochorella mangiferae* | MFLUCC 12-0310 | KF827444 | KF827477 | KF827445 | - | KF827479 | Dead leaf | Jiang et al. (2022) |
| Xylariomycetidae | Amphisphaeriales | Sporocadaceae | *Discosia brasiliensis* | MFLUCC 12-0431 | KF827433 | KF827466 | KF827437 | - | KF827474 | Dead leaf | Razaghi et al. (2024) |
| Xylariomycetidae | Diaporthales | Diaporthaceae | *Mazzantia napelli* | AR 3498 | - | EU222017 | AF408368 | - | EU219345 | *Aconitum lycoctonum* | Sogonov et al. (2008) |
| Xylariomycetidae | Xylariales | Diatrypaceae | *Allocryptovalsa sichuanensis* | HKAS 107017 | MW240633 | MW759517 | MW240563 | - | MW658624 | Dead wood | Samarakoon et al. (2022) |
| Xylariomycetidae | Xylariales | Diatrypaceae | *Diatrype disciformis* | AFTOL-ID 927 | - | DQ471085 | DQ470964 | DQ471158 | DQ470915 | Alnus sp. | Spatafora et al. (2006) |
| Xylariomycetidae | Xylariales | Lopadostomataceae | *Lopadostoma turgidum* | CBS 133207 | KC774618 | - | KC774618 | MK523270 | KC774563 | *Fagus sylvatica* | Jaklitsch et al. (2014) |
| Xylariomycetidae | Xylariales | Microdochiaceae | *Microdochium nivale* | CBS 116205 | KP859008 | - | KP858944 | - | KP859117 | Wheat Triticum aestivum roots | Hernández-Restrepo et al. (2016) |
| Xylariomycetidae | Xylariales | Xylariaceae | *Nemania thailandensis* | MFLU 19-2117 | MW240611 | MW759494 | MW240540 | - | MW342615 | Dead stem | Samarakoon et al. (2022) |
| Xylariomycetidae | Xylariales | Xylariaceae | *Xylaria bambusicola* | MFLUCC 11-0606 | KU940160 | - | KU863148 | - | KU940183 | Bamboo | Dai et al. (2017) |
