# Supplementary material for: Taxonomy, phylogeny, and evolutionary diversification of spider-pathogenic fungi from China (Hypocreales, Ascomycota)
Source: IMA Fungus. 2026 Apr 6;17:e171548. doi: 10.3897/imafungus.17.171548 (PMC13077317; doi:10.3897/imafungus.17.171548)
Supplement: Supplementary material 3 — Single-gene phylogenetic tree of the new species [file imafungus-17-e171548-s003.docx]

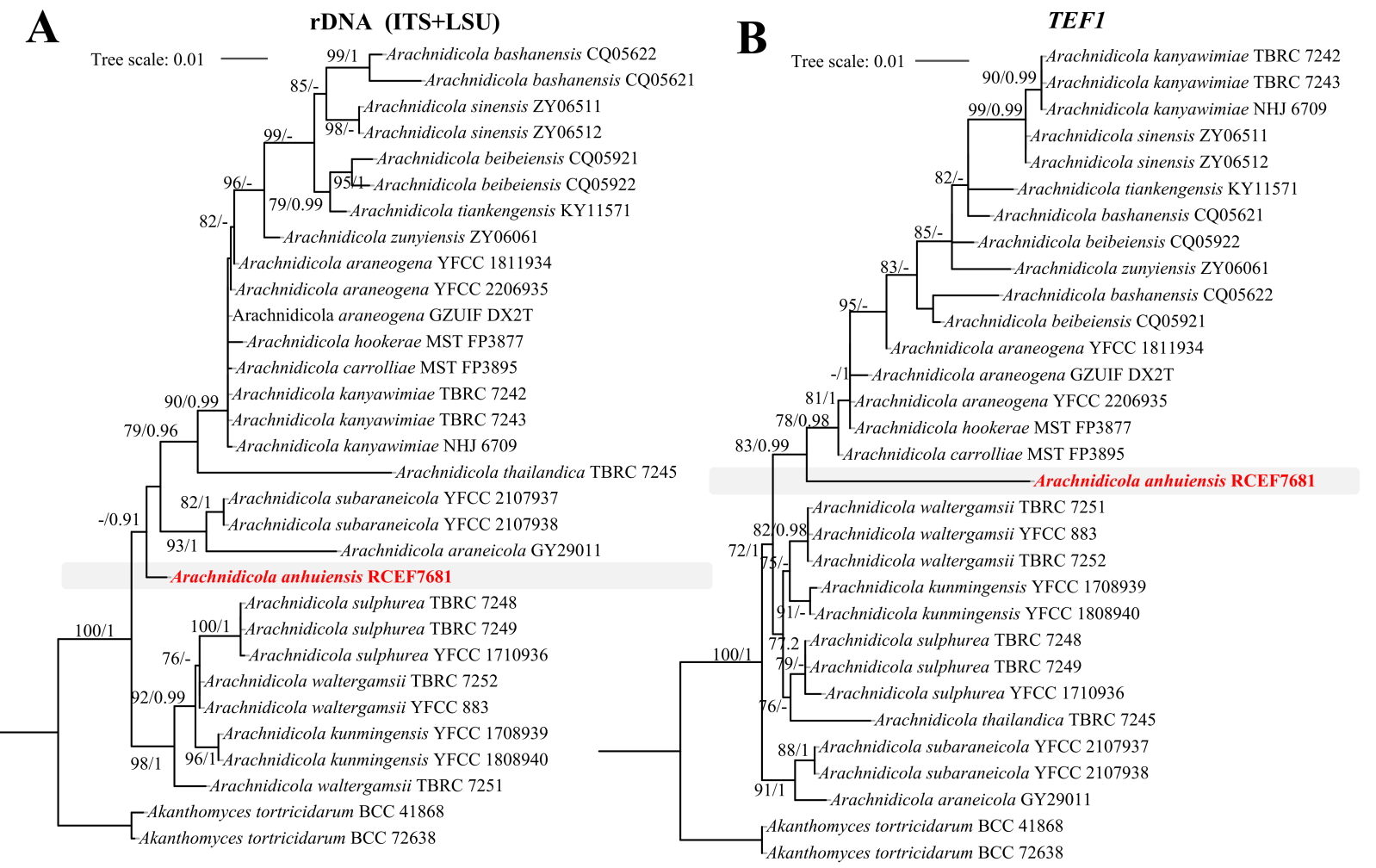


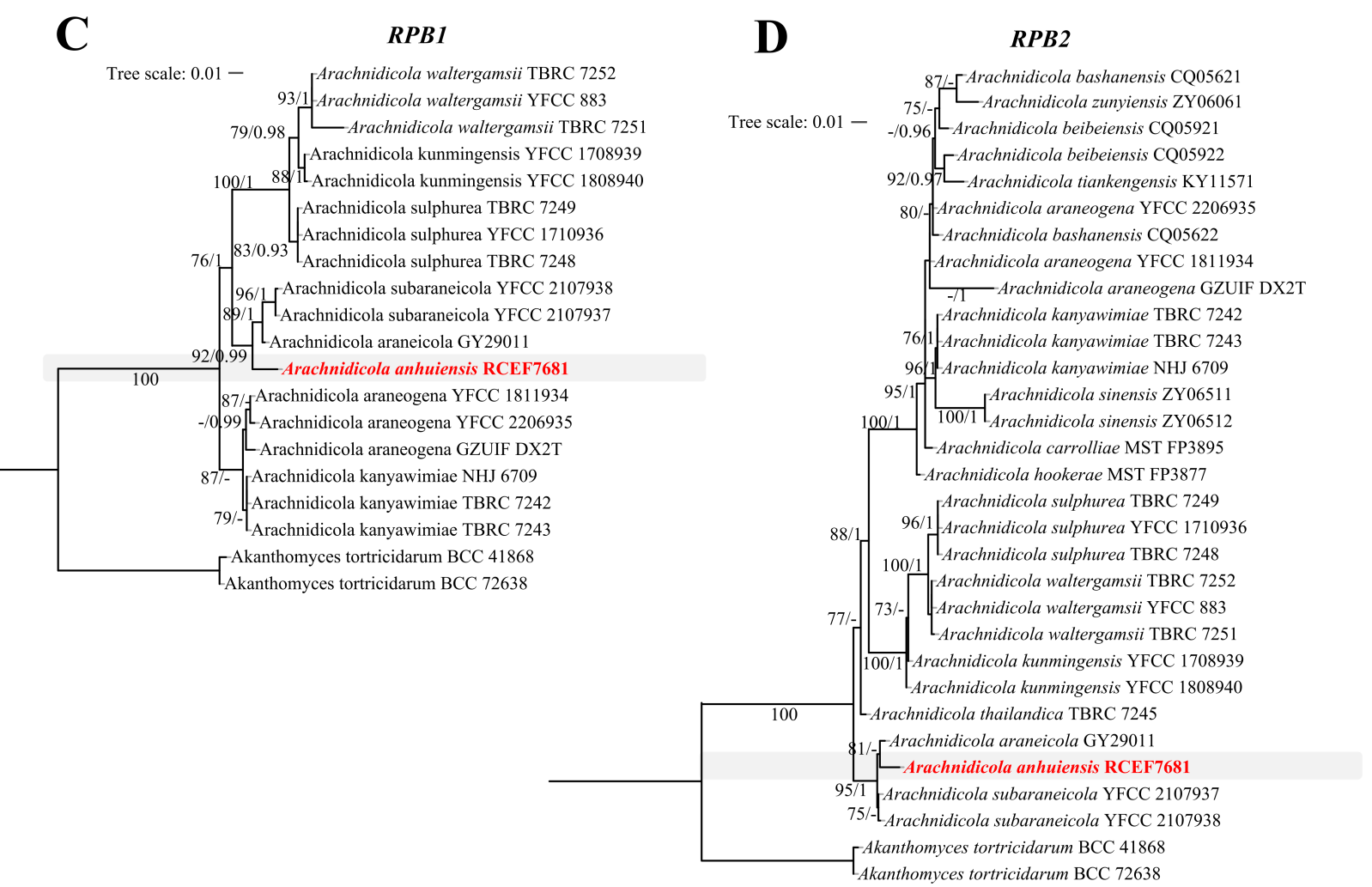


**Figure S2.** Single-gene phylogenetic trees illustrating the phylogenetic relationships among species within the genus *Arachnidicola*. (A) Phylogenetic tree constructed using the rDNA (ITS+LSU) gene region. (B) Phylogenetic tree constructed using the *TEF1* gene region. (C) Phylogenetic tree constructed using the *RPB1* gene region. (D) Phylogenetic tree constructed using the *RPB2* gene region.


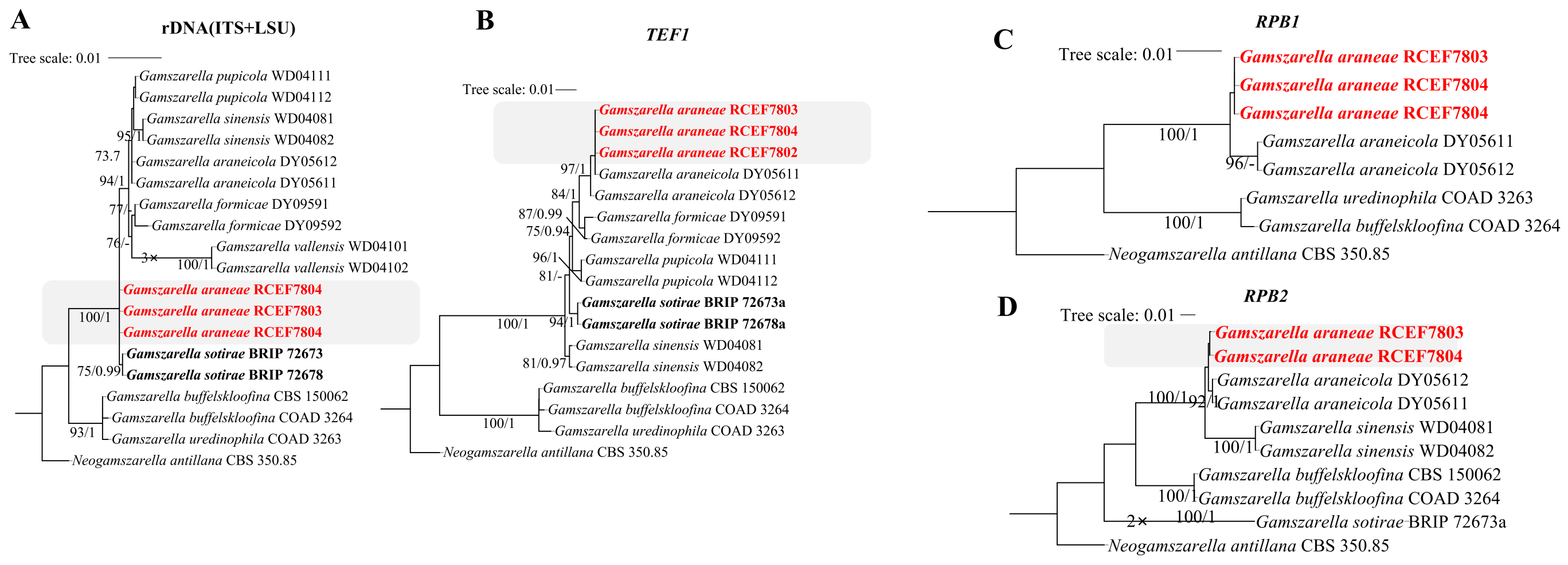


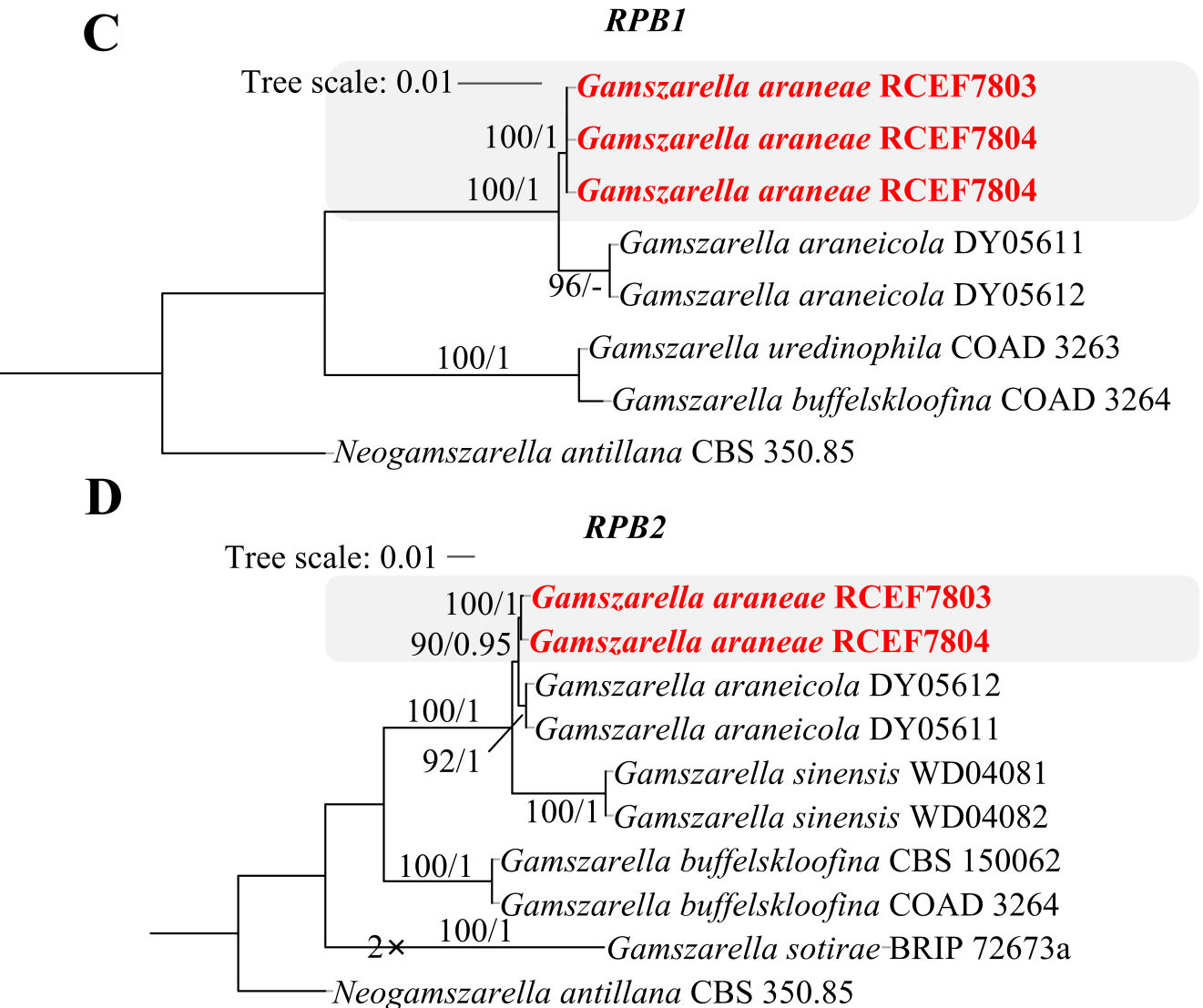


**Figure S3.** Single-gene phylogenetic trees illustrating the phylogenetic relationships among species within the genus *Gamszarella*. (A) Phylogenetic tree constructed using the rDNA (ITS+LSU) gene region. (B) Phylogenetic tree constructed using the *TEF1* gene region. (C) Phylogenetic tree constructed using the *RPB1* gene region. (D) Phylogenetic tree constructed using the *RPB2* gene region.


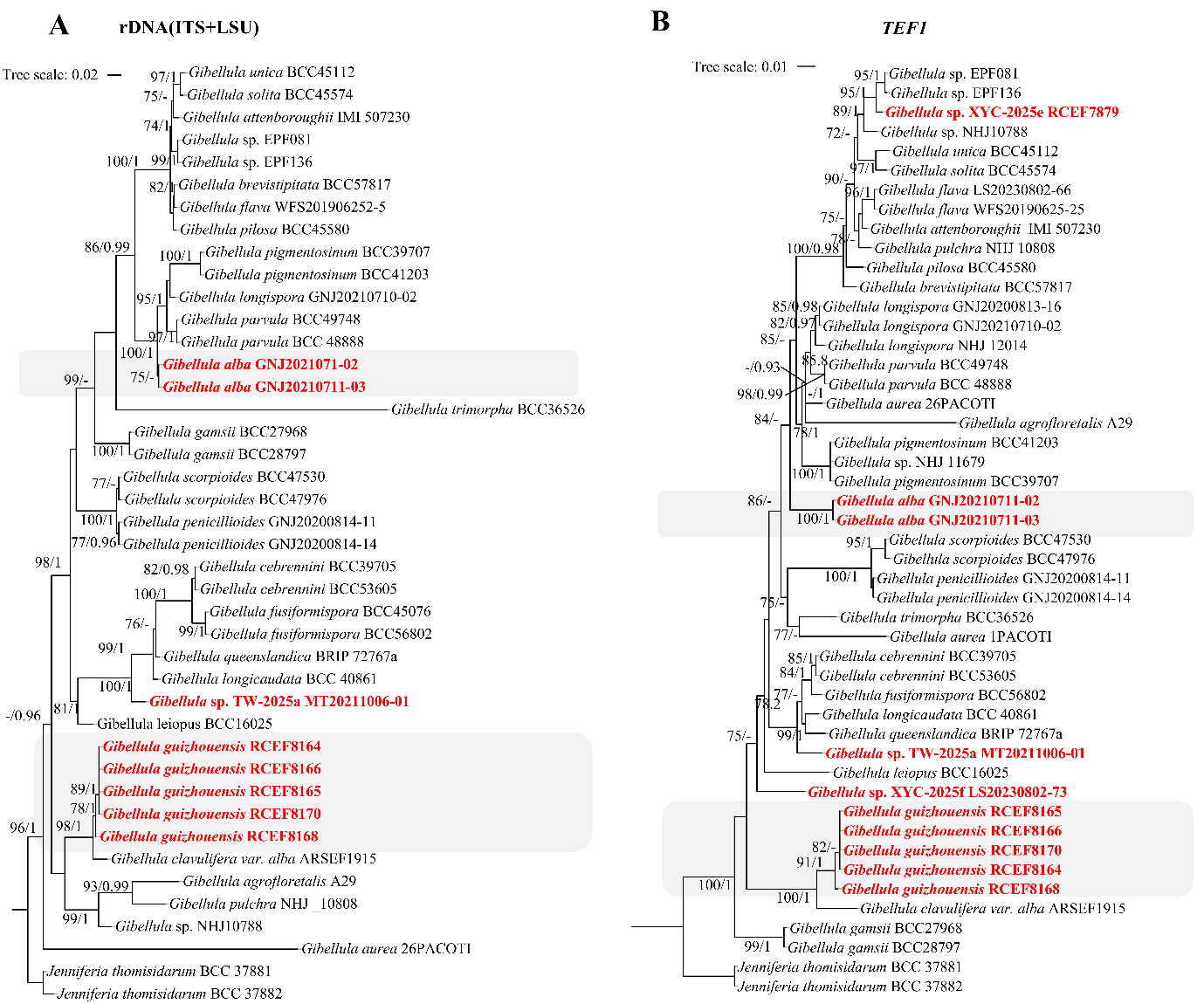

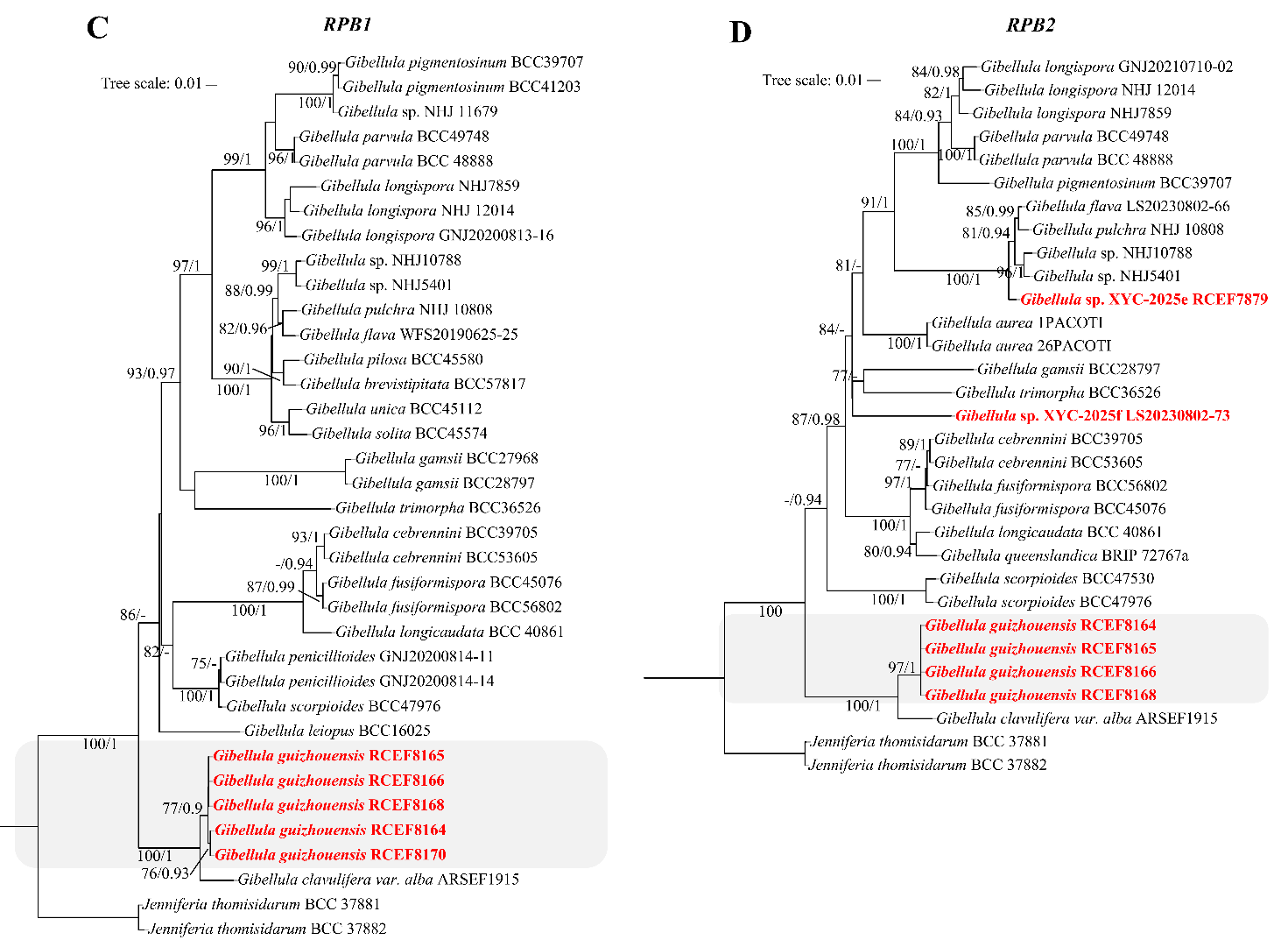


**Figure S4.** Single-gene phylogenetic trees illustrating the phylogenetic relationships among species within the genus Gibellula . (A) Phylogenetic tree constructed using the rDNA (ITS+LSU) gene region. (B) Phylogenetic tree constructed using the *TEF1* gene region. (C) Phylogenetic tree constructed using the *RPB1* gene region. (D) Phylogenetic tree constructed using the *RPB2* gene region.


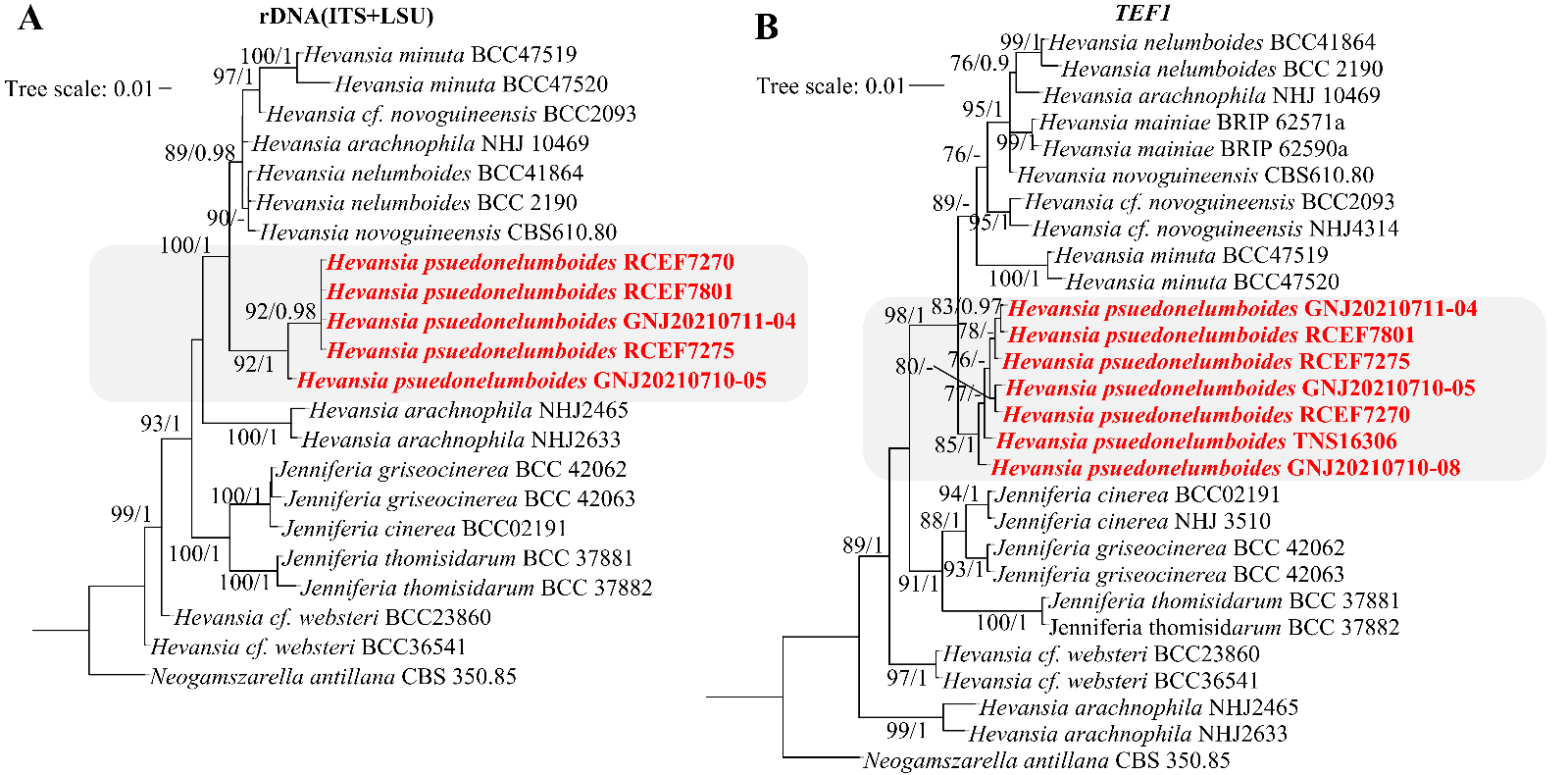

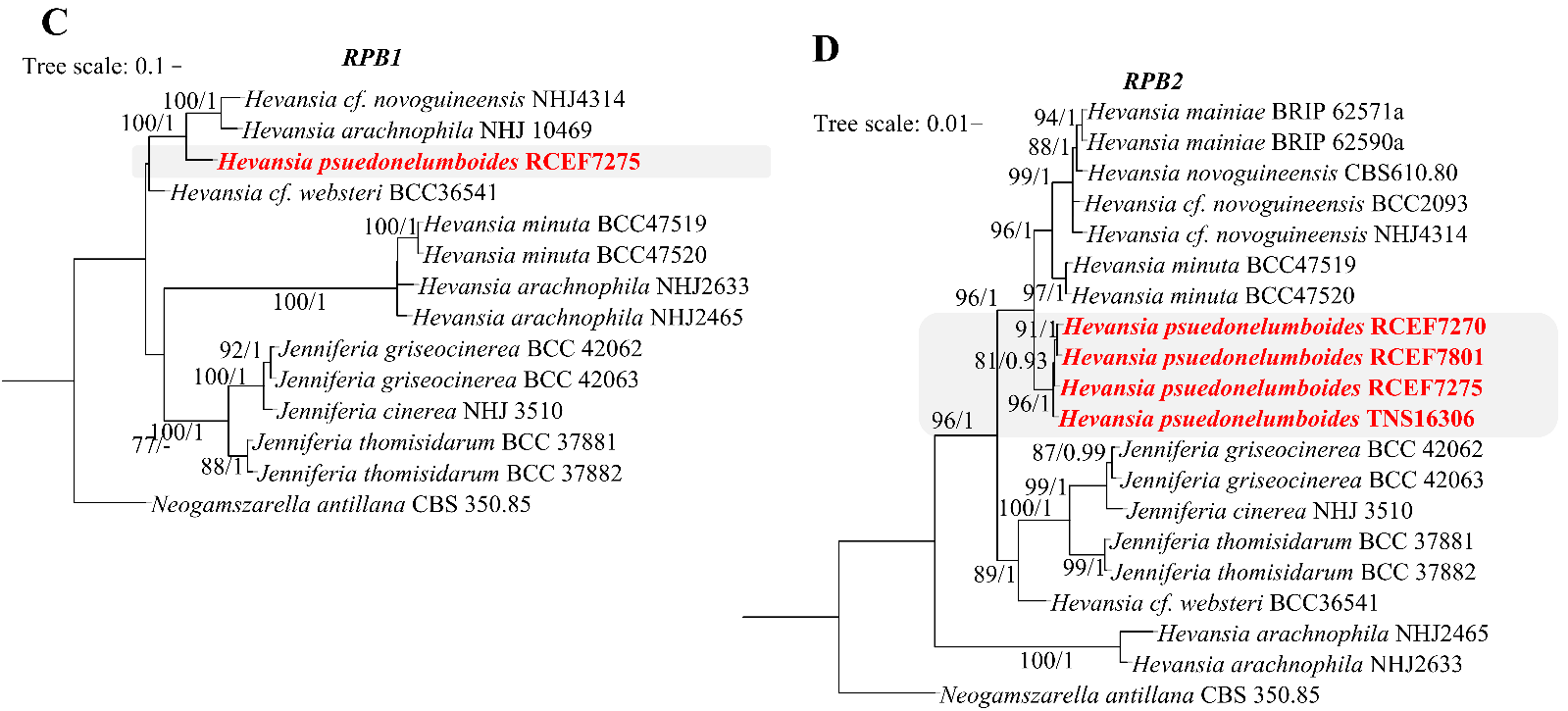


**Figure S5.** Single-gene phylogenetic trees illustrating the phylogenetic relationships among species within the genus *Hevansia*. (A) Phylogenetic tree constructed using the rDNA (ITS+LSU) gene region. (B) Phylogenetic tree constructed using the *TEF1* gene region. (C) Phylogenetic tree constructed using the *RPB1* gene region. (D) Phylogenetic tree constructed using the *RPB2* gene region.

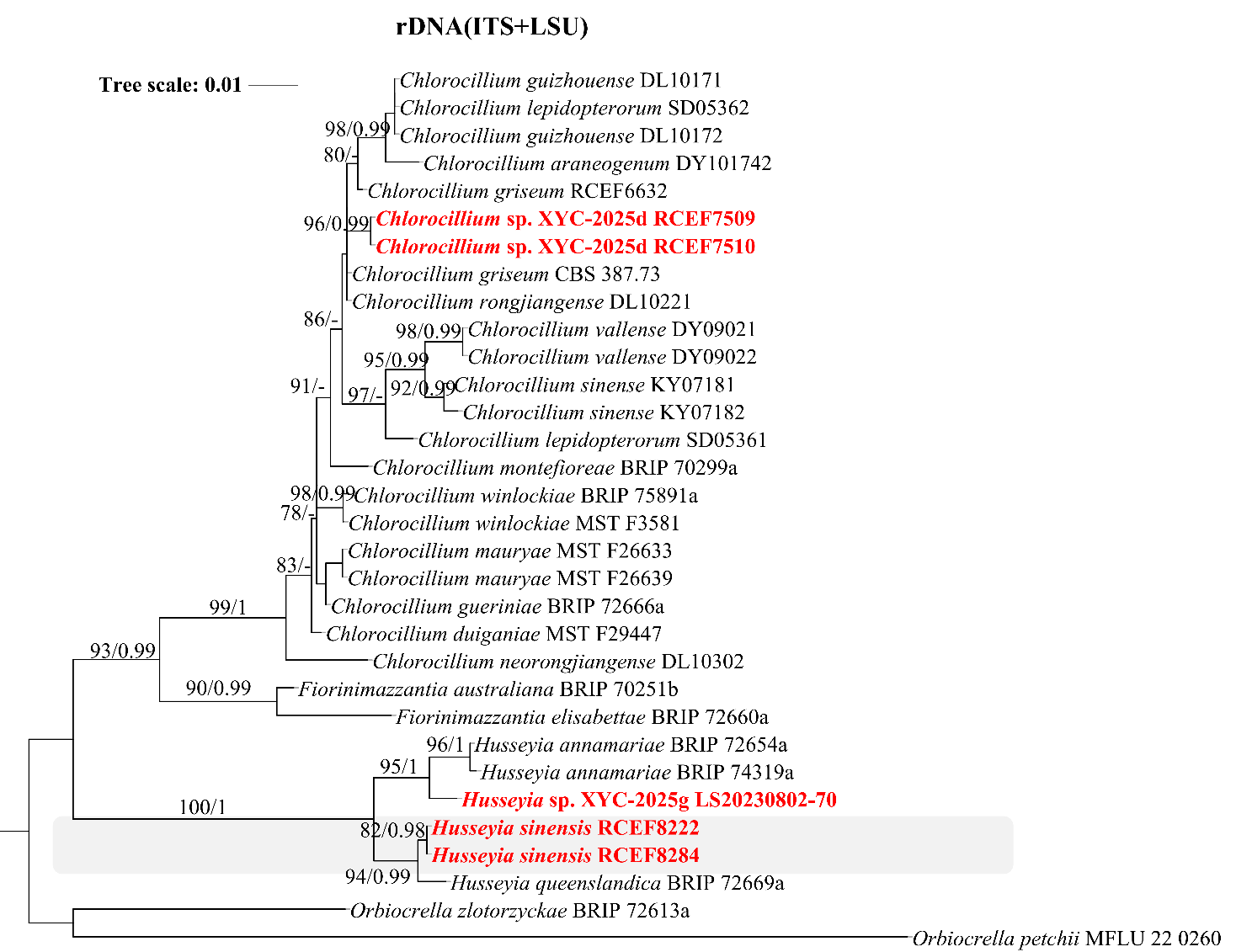


A


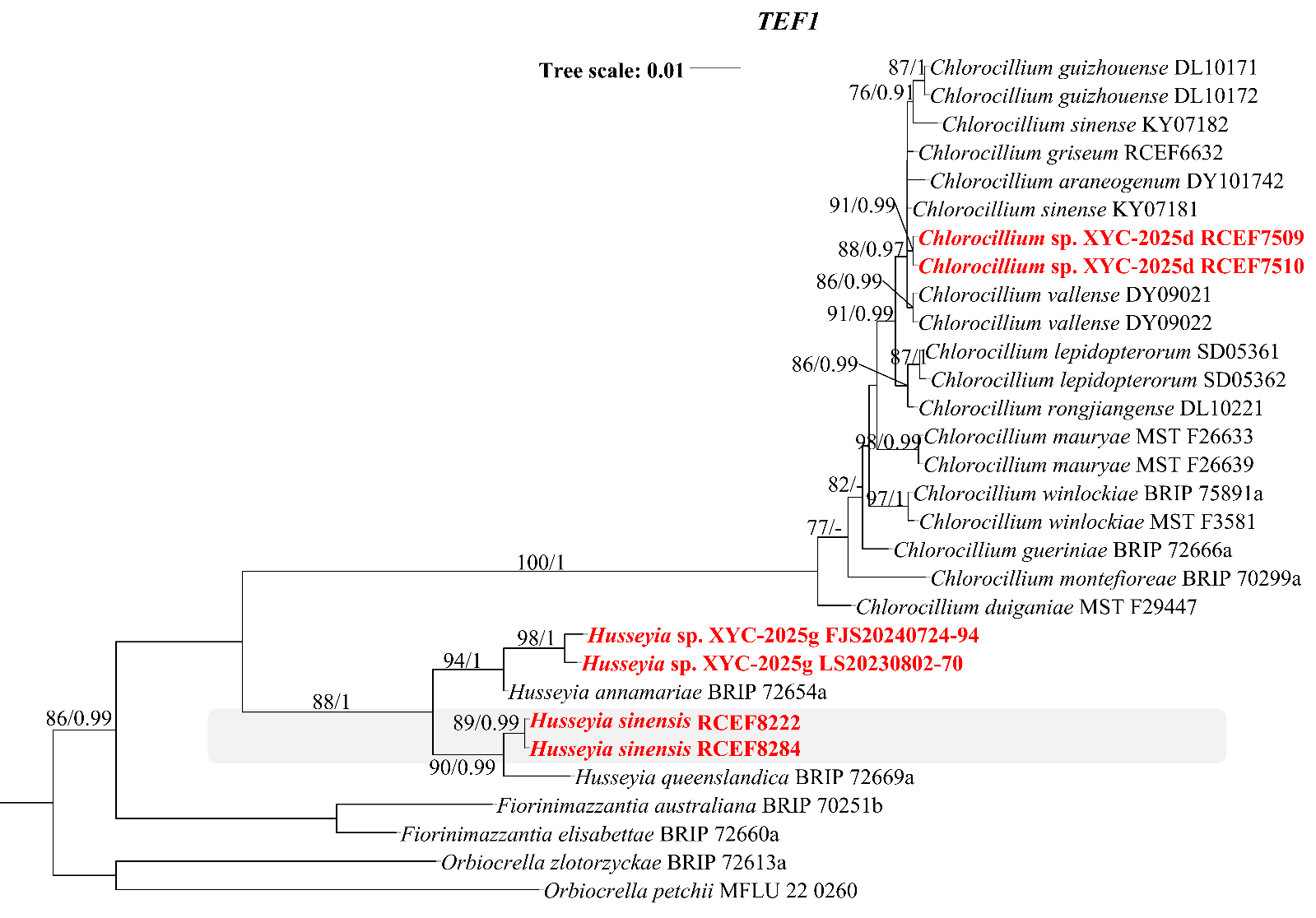

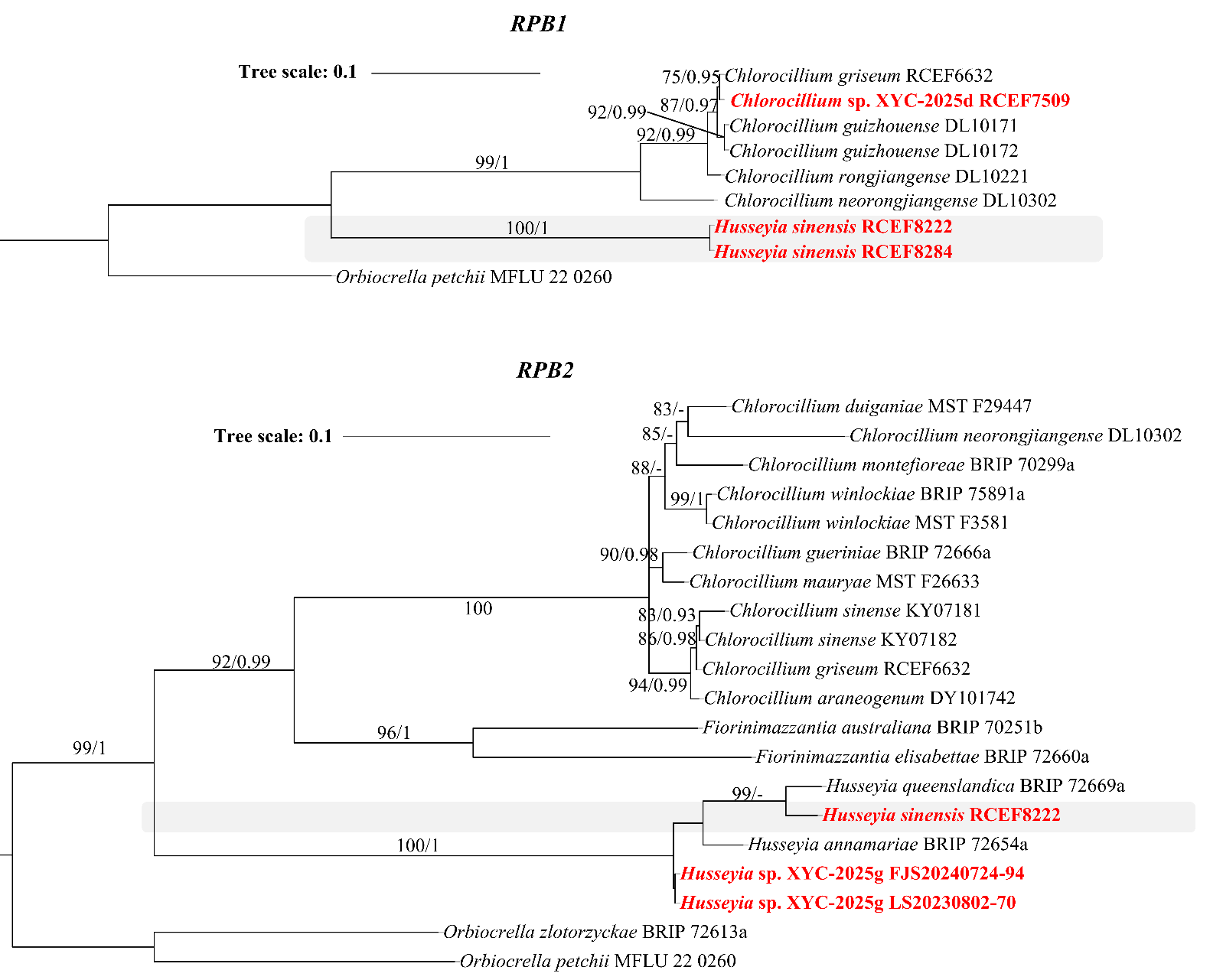


B

D

C

**Figure S6.** Single-gene phylogenetic trees illustrating the phylogenetic relationships among species within the genus *Chlorocillium* and *Husseyia*. (A) Phylogenetic tree constructed using the rDNA (ITS+LSU) gene region. (B) Phylogenetic tree constructed using the *TEF1* gene region. (C) Phylogenetic tree constructed using the *RPB1* gene region. (D) Phylogenetic tree constructed using the *RPB2* gene region.


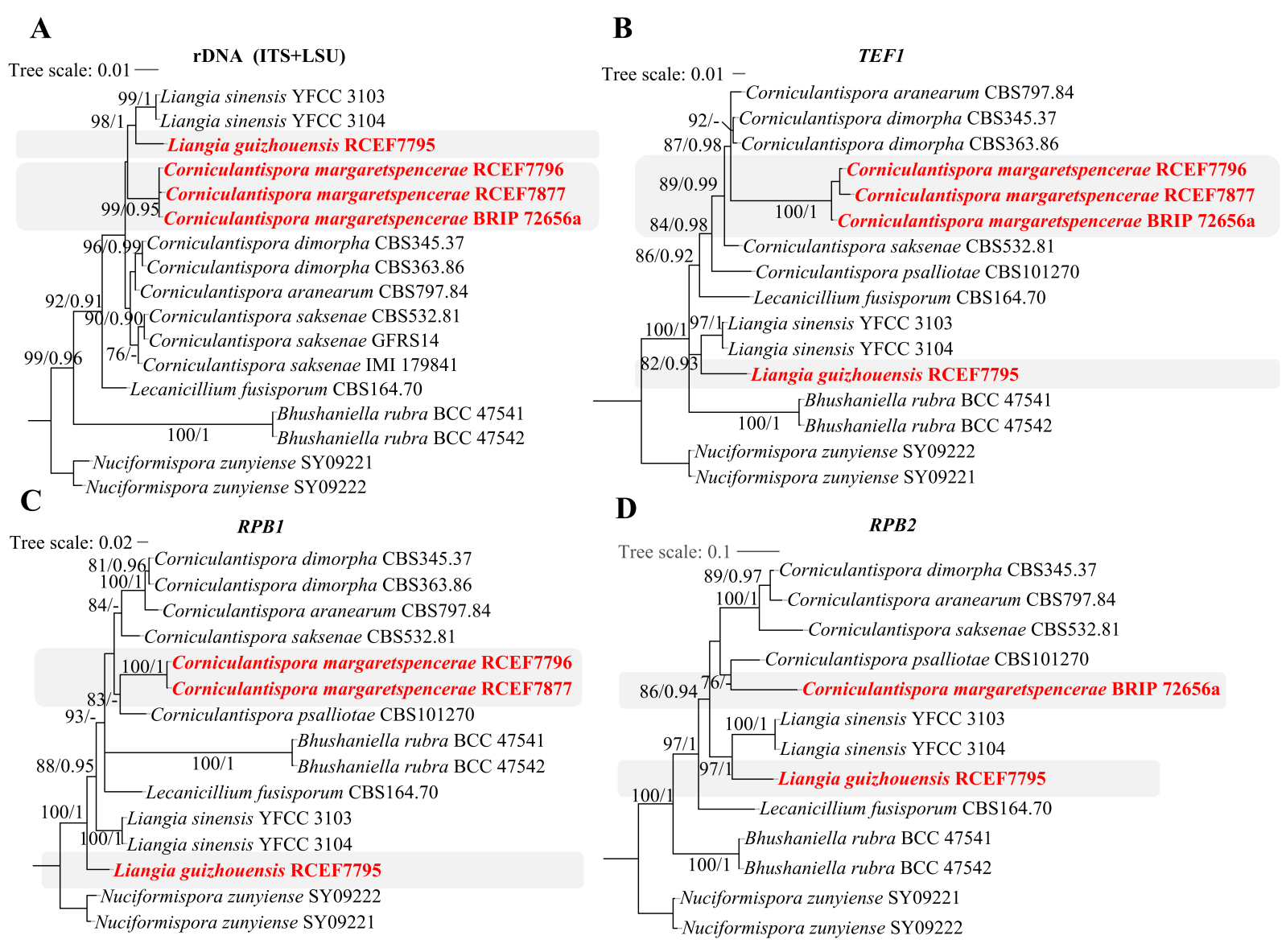


**Figure S7.** Single-gene phylogenetic trees illustrating the phylogenetic relationships among species within the genus *Liangia*. (A) Phylogenetic tree constructed using the rDNA (ITS+LSU) gene region. (B) Phylogenetic tree constructed using the *TEF1* gene region. (C) Phylogenetic tree constructed using the *RPB1* gene region. (D) Phylogenetic tree constructed using the *RPB2* gene region.


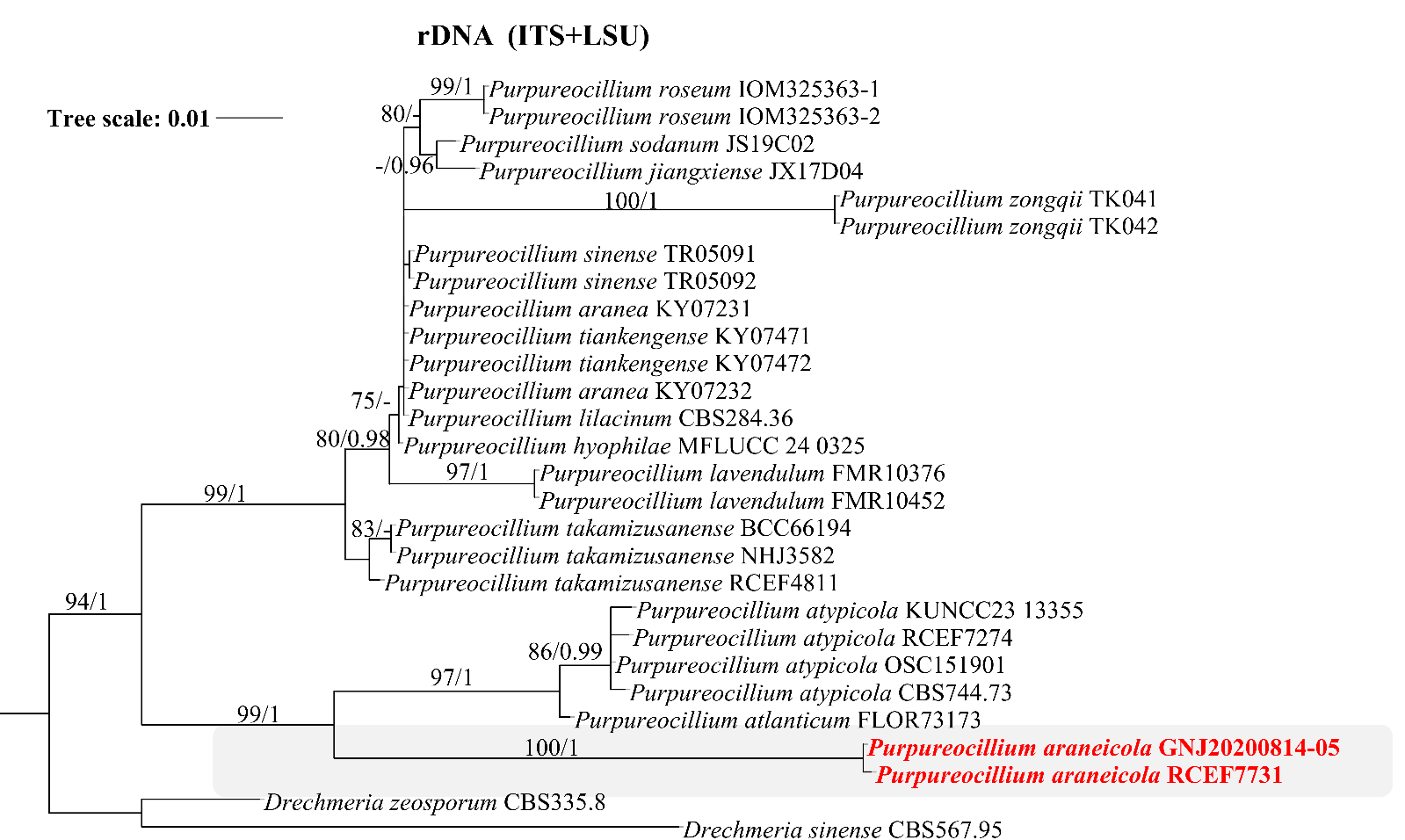


**A**


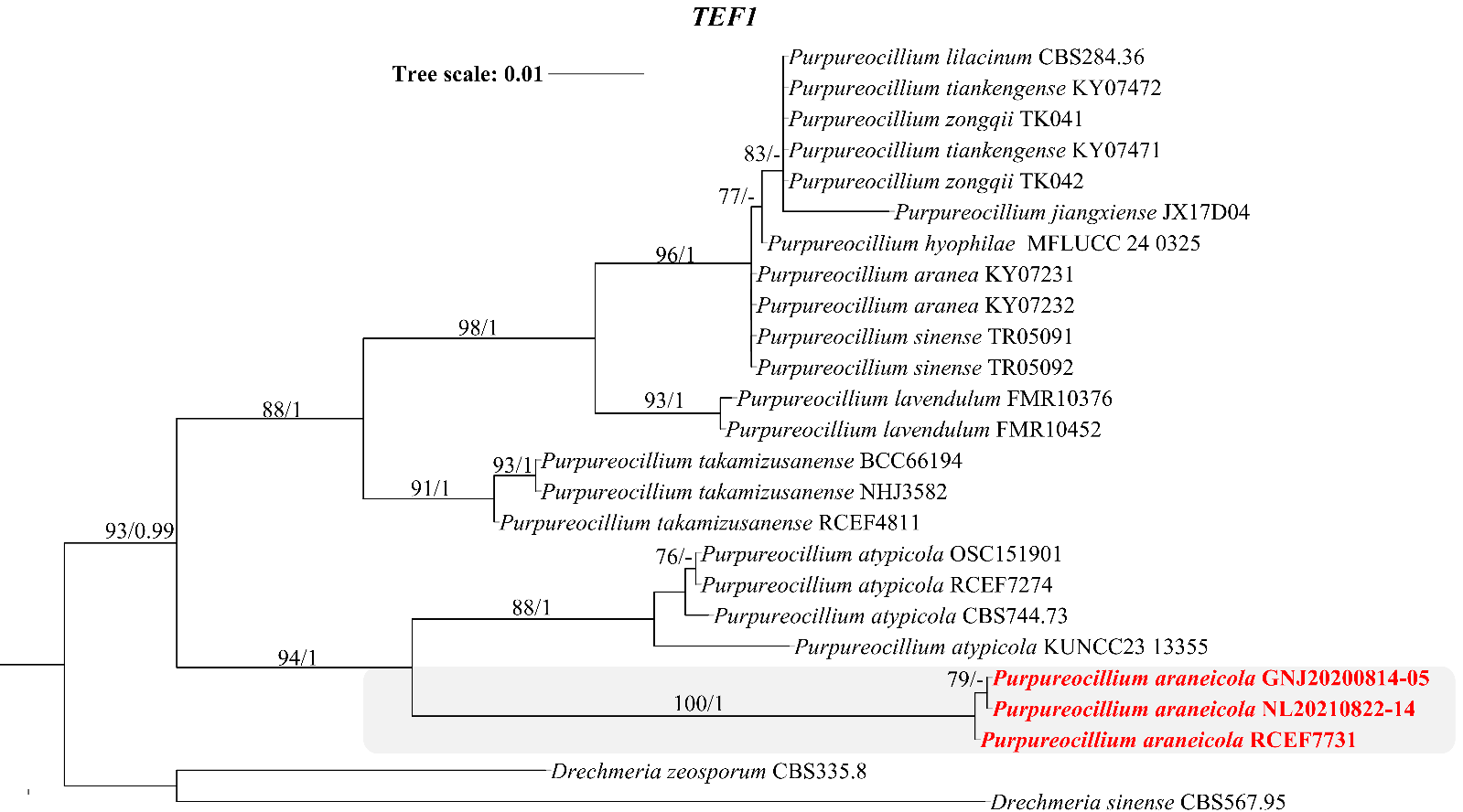

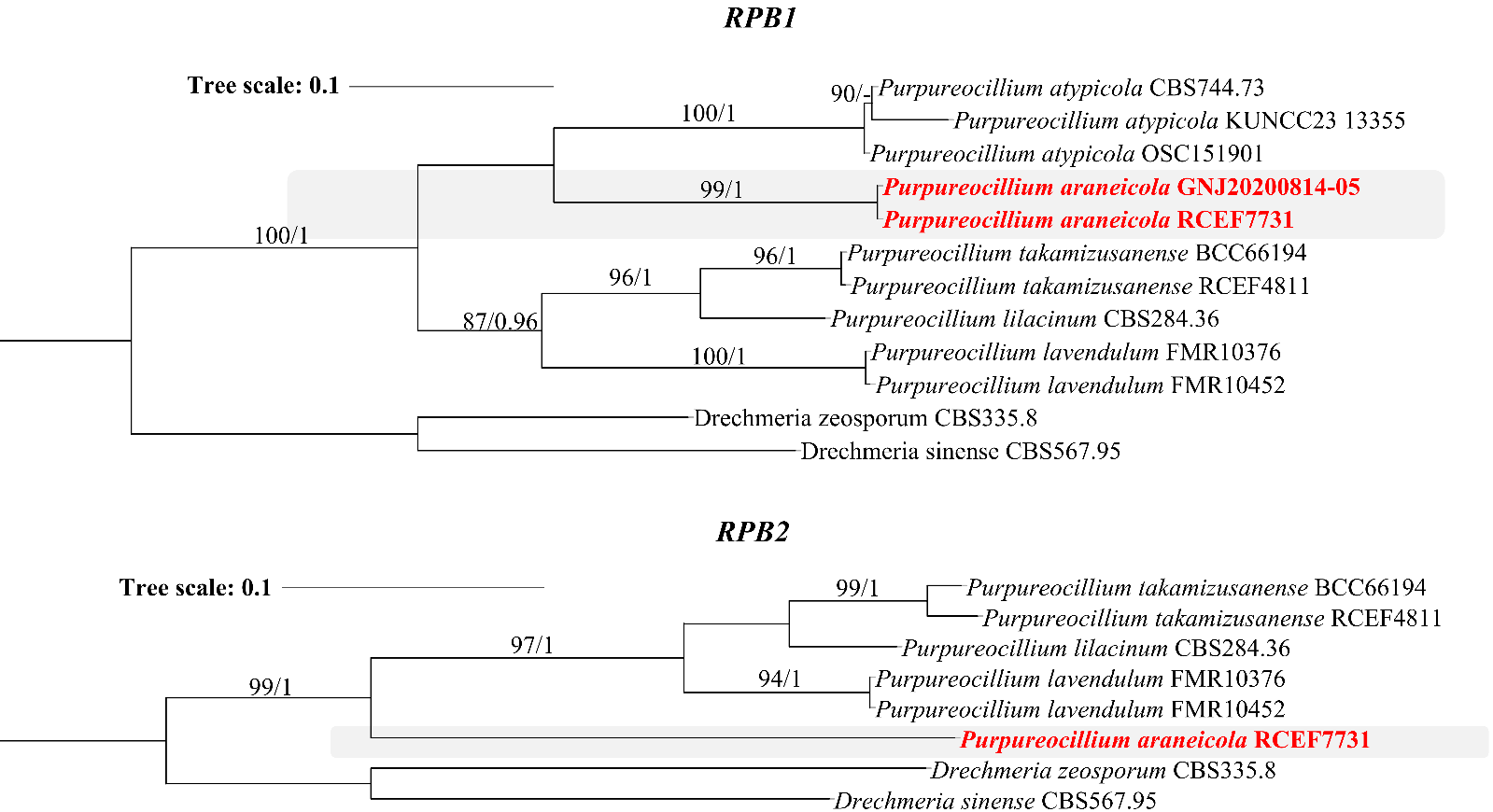


**B**

**D**

**C**

**Figure S8.** Single-gene phylogenetic trees illustrating the phylogenetic relationships among species within the genus *Purpureocillium*. (A) Phylogenetic tree constructed using the rDNA (ITS+LSU) gene region. (B) Phylogenetic tree constructed using the *TEF1* gene region. (C) Phylogenetic tree constructed using the *RPB1* gene region. (D) Phylogenetic tree constructed using the *RPB2* gene region.

**Table S4.** Genealogical concordance among single-locus phylogenies supporting species delimitation under GCPSR

| Species | Maximum likelihood bootstrap support (MLBS) / Bayesian posterior probability (BPP) | | | | |
| --- | --- | --- | --- | --- | --- |
|  | ITS+nrLSU (rDNA) | *TEF1* | *RPB1* | *RPB2* | Concordant loci |
| *Arachnidicola anhuiensis* | - / 0.91 | 83 / 0.99 | 92 / 0.99 | 81 / - | ITS+LSU (rDNA), *TEF1*, *RPB1*, *RPB2* |
| *Gamszarella araneae* | 100 / 1 | Conflict | 100 / 1 | 90 / 0.95 | ITS+LSU (rDNA), *RPB1*, *RPB2* |
| *Gibellula alba* | 75 / 1 | 100 / 1 | Sequence missing | Sequence missing | ITS+LSU (rDNA), *TEF1* |
| *Gibellula guizhouensis* | 98 / 1 | 91 / 1 | 77 / 0.90 | 97 / 1 | ITS+LSU (rDNA), *TEF1*, *RPB1*, *RPB2* |
| *Hevansia psuedonelumboides* | 92 / 1 | 85 / 1 | 100 / 1 | 96 / 1 | ITS+LSU (rDNA), *TEF1*, *RPB1*, *RPB2* |
| *Husseyia sinensis* | 94 / 0.99 | 89 / 0.99 | 100 / 1 | 100 / 1 | ITS+LSU (rDNA), *TEF1*, *RPB1*, *RPB2* |
| *Liangia guizhouensis* | 98 / 1 | 82 / 0.93 | 100 / 1 | 97 / 1 | ITS+LSU (rDNA), *TEF1*, *RPB1*, *RPB2* |
| *Purpureocillium araneicola* | 100 / 1 | 100 / 1 | 99 / 1 | 99 / 1 | ITS+LSU (rDNA), *TEF1*, *RPB1*, *RPB2* |

Note: Only MLBS ≥ 75% and BPP values ≥ 0.90 are shown.
